# Prolidase Stimulates Proliferation and Migration through Activation of the PI3K/Akt/mTOR Signaling Pathway in Human Keratinocytes

**DOI:** 10.3390/ijms21239243

**Published:** 2020-12-03

**Authors:** Magdalena Misiura, Weronika Baszanowska, Ilona Ościłowska, Jerzy Pałka, Wojciech Miltyk

**Affiliations:** 1 Department of Analysis and Bioanalysis of Medicines, Medical University of Bialystok, Jana Kilińskiego 1, 15-089 Bialystok, Poland; magdalena.misiura@umb.edu.pl; 2 Department of Medicinal Chemistry, Medical University of Bialystok, Jana Kilińskiego 1, 15-089 Bialystok, Poland; w.baszanowska22@wp.pl (W.B.); ilona.zareba@gmail.com (I.O.); pal@umb.edu.pl (J.P.)

**Keywords:** prolidase, PEPD, EGFR, keratinocytes, wound healing

## Abstract

Recent reports have indicated prolidase (PEPD) as a ligand of the epidermal growth factor receptor (EGFR). Since this receptor is involved in the promotion of cell proliferation, growth, and migration, we aimed to investigate whether prolidase may participate in wound healing in vitro. All experiments were performed in prolidase-treated human keratinocytes assessing cell vitality, proliferation, and migration. The expression of downstream signaling proteins induced by EGFR, insulin-like growth factor 1 (IGF-1), transforming growth factor β_1_ (TGF-β_1_), and β_1_-integrin receptors were evaluated by Western immunoblotting and immunocytochemical staining. To determine collagen biosynthesis and prolidase activity radiometric and colorimetric methods were used, respectively. Proline content was determined by applying the liquid chromatography coupled with mass spectrometry. We found that prolidase promoted the proliferation and migration of keratinocytes through stimulation of EGFR-downstream signaling pathways in which the PI3K/Akt/mTOR axis was involved. Moreover, PEPD upregulated the expression of β_1_-integrin and IGF-1 receptors and their downstream proteins. Proline concentration and collagen biosynthesis were increased in HaCaT cells under prolidase treatment. Since extracellular prolidase as a ligand of EGFR induced cell growth, migration, and collagen biosynthesis in keratinocytes, it may represent a potential therapeutic approach for the treatment of skin wounds.

## 1. Introduction

Wound healing is a physiological process restoring skin functionality. It consists of four phases occurring in proper time and order. This process starts from hemostasis, then inflammation followed by proliferation and eventually tissue remodeling. The sequence of events during wound healing is strictly programmed and any disturbances may impair normal tissue repair. In the microenvironment of the damaged tissue, there are numerous biological factors and cell types involved in this process. One of them is keratinocytes, which proliferate and migrate to the wounded area during the proliferative phase induced by growth factors [1].

Stimulated epidermal growth factor receptor (EGFR) and its downstream signaling proteins are responsible for cell proliferation, differentiation, growth, and migration [2]. Recent reports have demonstrated that prolidase (PEPD) is a ligand of EGFR [3]. PEPD is a protein that evokes intracellular and extracellular functions. Intracellularly it has an enzymatic activity [EC.3.4.13.9] splitting imidodipeptides with C-terminal proline or hydroxyproline [4,5] supplying proline for protein biosynthesis, mainly collagen. Moreover, this enzyme is involved in the regulation of cell growth at transcriptional (e.g., nuclear factor kappa beta (NF-κβ)) and post-transcriptional (e.g., hypoxia-inducible factor 1 alpha (HIF-1α)) levels [6,7,8,9]. Extracellularly, prolidase as a ligand of EGFR induces growth-promoting signaling [3]. Both functions, intracellular and extracellular contribute to the upregulation of anabolic processes. Therefore, prolidase expression and the enzyme activity could be of great importance in tissue regeneration processes. Although the intracellular function of prolidase in collagen biosynthesis is well recognized [10], its role in the activation of EGFR-dependent signaling in an experimental model of wound healing has not been established.

EGFR, known also as ErbB, is a member of four related tyrosine kinase receptors that upon ligand binding undergo dimerization leading to receptor autophosphorylation. This process initiates a cascade of phosphorylation of many signaling proteins inducing different cellular events [11]. The most specific signaling proteins downstream of EGFR are phosphoinositide 3-kinase (PI3K)/protein kinase B (Akt)/mammalian target of rapamycin (mTOR), Ras/Raf/extracellular signal-regulated kinase (ERK), and Janus kinase (JAK)/signal transducer and activator of transcription (STAT) pathways [12] that represent progrowth and proproliferation signaling. However, the signaling pathway could not function without cross-talk with adhesion receptors. In keratinocytes and fibroblasts, the most abundant are integrin receptors. In the context of the anabolic and growth-promoting activity, the signaling induced by the β_1_-integrin receptor is well characterized. This signaling pathway is known to induce prolidase activity [13] and collagen biosynthesis [14]. Upon stimulation, the β_1_-integrin receptor induces autophosphorylation of non-receptor focal adhesion kinase pp125FAK (FAK) that is specific for adhesion receptors. Then, the kinase can interact with growth factor receptor-bound protein 2 (Grb2), through proto-oncogene Src and Shc proteins. The further cascade of signaling pathway involves son of sevenless 1 (Sos1), Ras and Raf proteins, and subsequently two mitogen-activated protein (MAP) kinases: ERK1 and ERK2 [15,16]. This signaling pathway is amplified by EGFR signaling, leading to the induction of transcription factors that stimulate the expression of genes for several proteins involved in the regulation of cell growth, differentiation, and metabolism [17]. The evidence for the coordinate regulation of prolidase activity and collagen biosynthesis by β_1_-integrin signaling was documented [18]. Although the functional significance of EGFR and integrin signaling in anabolic and growth-promoting processes is well established, the mechanism for regulation of the processes in tissue regeneration is not well understood. Since prolidase is a ligand of EGFR and EGFR signaling activates cell proliferation, we hypothesized that extracellular prolidase may accelerate the experimental wound healing.

## 2. Results

### 2.1. Prolidase Promotes Proliferation and Migration of HaCaT Keratinocytes in a Model of Wound Healing

**Figure 1:**
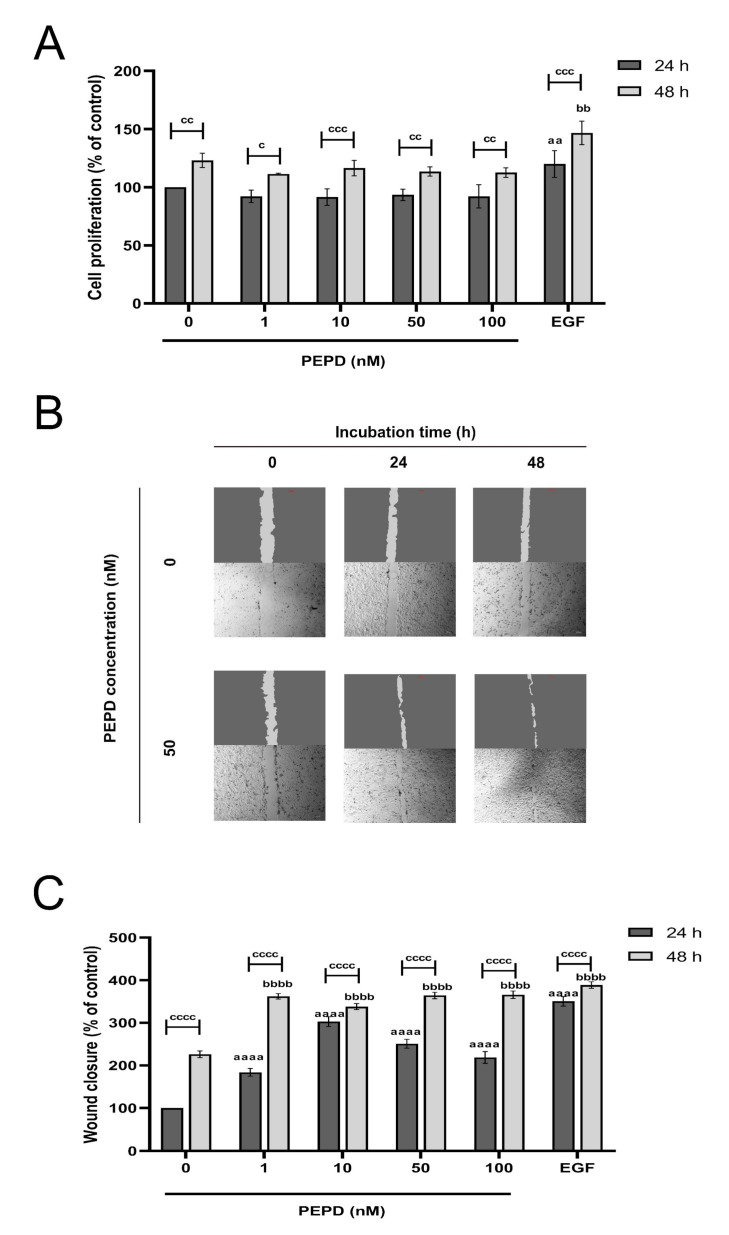
Prolidase promotes proliferation and migration of HaCaT cell lines. (**A**) Cell proliferation was evaluated by CyQuant Proliferation assay after 24 and 48 h upon prolidase supplementation. (**B**) Microscopic images of wound closure in scratched assay monitored using an inverted microscope (40× magnification) after 24 and 48 h incubation with prolidase (50 nM) compared to control cells. Supplementary Materials contain microscopic images in triplicates (Supplementary Figure S1). (**C**) The wound closure rate was evaluated by ImageJ software in HaCaT cells treated for 24 and 48 h with prolidase at concentrations of 1–100 nM. EGF was used as a positive control. A mean ± SEM of three replicates is presented. The results are significant at *c* < 0.05, *aa*, *bb*, or *cc* < 0.01, *ccc* < 0.001, and *aaaa*, *bbbb*, or *cccc* < 0.0001 and are marked as *a* vs. control (0 nM of PEPD) of 24 h incubation, *b* vs. control (0 nM of PEPD) of 48 h incubation, and *c* marked 24 h incubation cells vs. 48 h incubation cells at the same concentration of PEPD or EGF, respectively.

During the re-epithelization phase of wound healing keratinocytes proliferate and migrate closing the wound and restoring the epithelial layer [19]. To test whether keratinocytes proliferate under conditions of mechanical damage we scratched the cell monolayer. DNA fluorescence assay was employed to study the effect of prolidase on the proliferation of keratinocytes. The cells were treated with various concentrations of prolidase (1–100 nM) for 24 and 48 h. As a positive control for cell proliferation, the epidermal growth factor (EGF) at a concentration of 10 nM was used. As shown in Figure 1A, prolidase at all studied concentrations similarly stimulated cell proliferation, however, exhibited lower potency than EGF. It was found that the proliferation of keratinocytes was noticeably increased in a time-dependent manner.

Subsequently, we investigated whether prolidase stimulates the migration of HaCaT cells followed by a scratch of the cell monolayer using an in vitro wound closure/scratch assay. EGF (10 nM), as a factor promoting cell proliferation, was used as a positive control in a model of wound closure. The results demonstrated that prolidase contributed to progressive wound closure in a dose- and time-dependent manner. As presented in Figure 1B prolidase treatment of the cells for 48 h resulted in almost complete closure of the wound, whereas the wounded area of control cells (without prolidase) remained unchanged. Quantification of the wounded area revealed that HaCaT cells subjected to prolidase treatment migrated faster to the wounded area than those in control. After 48 h incubation, the rate of wound closure was similar for both EGF and prolidase (Figure 1C).

### 2.2. Prolidase Does not Change the Vitality of HaCaT Keratinocytes

**Figure 2:**
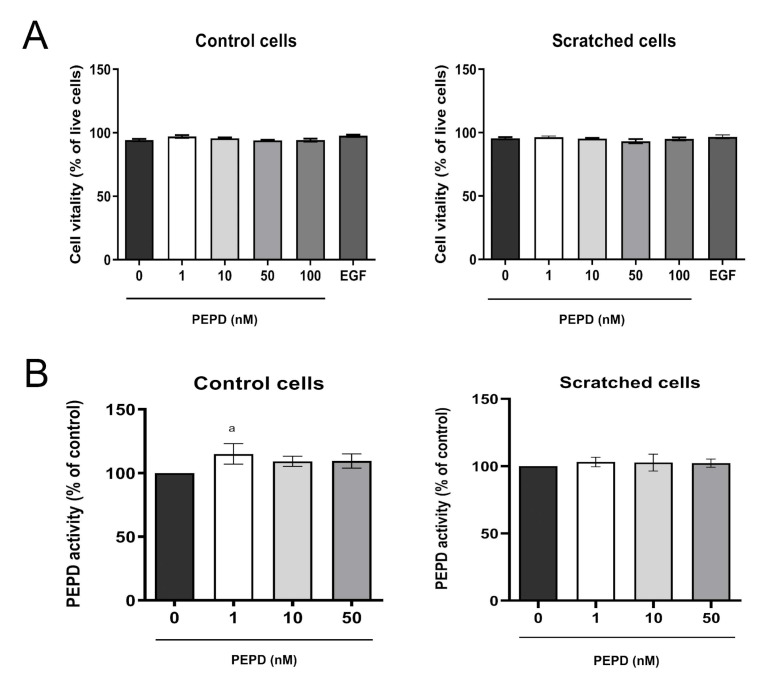
Prolidase does not affect the vitality and prolidase activity of keratinocytes. (**A**) Cell vitality and (**B**) PEPD activity in control and scratched HaCaT cells, treated with different concentrations of prolidase (1–100 nM) and EGF (10 nM) for 24 h. A mean ± SEM of three replicates is presented. The results are significant at *a* < 0.05. Statistically significant differences were calculated vs. control (0 nM of PEPD).

The effect of prolidase on cell vitality was determined by the measurement of intracellular thiols concentration. As demonstrated in Figure 2A, prolidase treatment (1–100 nM, 24 h) did not affect cell vitality in control and scratched cell models.

### 2.3. Extracellular Prolidase does not Affect Intracellular Prolidase Activity in Keratinocytes

Prolidase exhibits dual biological activity as an enzyme and an EGFR ligand [20]. To exclude the possibility that extracellular prolidase affects intracellular prolidase activity, the enzyme activity was measured in prolidase treated keratinocytes. There was no difference in prolidase activity upon prolidase treatment (1–50 nM, 24 h) of HaCaT cells in both control and scratched cell models (Figure 2B).

### 2.4. Prolidase Activates EGFR and Downstream Signaling Proteins in HaCaT Cells

**Figure 3:**
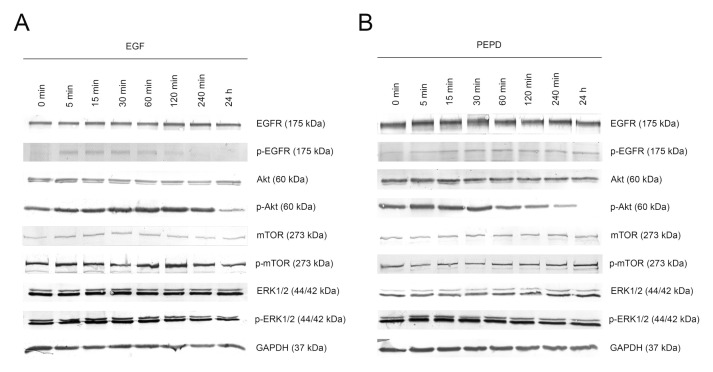
Kinetic analysis of phosphorylation of the epidermal growth factor receptor (EGFR) and its downstream signaling proteins induced by EGF (**A**) and PEPD (**B**) at concentration of 10 nM after 0, 5, 15, 30, 60, 120, and 240 min and 24 h upon supplementation. Representative blot images are shown (densitometric analysis of presented protein is included in Supplementary Materials (Supplementary Figure S2)).

The potency of PEPD to phosphorylate EGFR and downstream signaling proteins was compared to the activity of EGF (10 nM), used as a positive control. Kinetic analysis of the activation of the EGFR signaling pathway in a time course of 0, 5, 15, 30, 60, 120, and 240 min and 24 h was performed using Western immunoblotting. The time course of EGFR activation by these ligands was similar indicating that PEPD is a potent inducer of this receptor. Treatment of HaCaT cells with 10 nM PEPD resulted in significant phosphorylation of EGFR and its key signaling proteins such as mTOR, Akt, and ERK1/2. PEPD present in cell culture medium activated these molecules through phosphorylation of p-EGFR (Tyr1068), p-mTOR (Ser2448), p-Akt (Ser473), and p-ERK1/2 (Thr202/Tyr204; Figure 3A,B). In PEPD-treated HaCaT cells, EGFR was activated within 5 min upon treatment and then gradually decreased up to 4 h, similarly to the activation by EGF. We found that phosphorylation of EGFR instantly entailed phosphorylation of Akt and ERK in response to PEPD treatment. Whereas these signaling proteins were activated rapidly, activation of mTOR was slower, as p-mTOR reached a maximal level at 1 h after treatment with PEPD. These results demonstrated that prolidase could elicit EGFR transactivation leading to sustained phosphorylation of Akt/mTOR and MAPK signaling pathways in keratinocytes.

### 2.5. Prolidase Elicits Biological Effects in HaCaT Cells through EGFR

**Figure 4:**
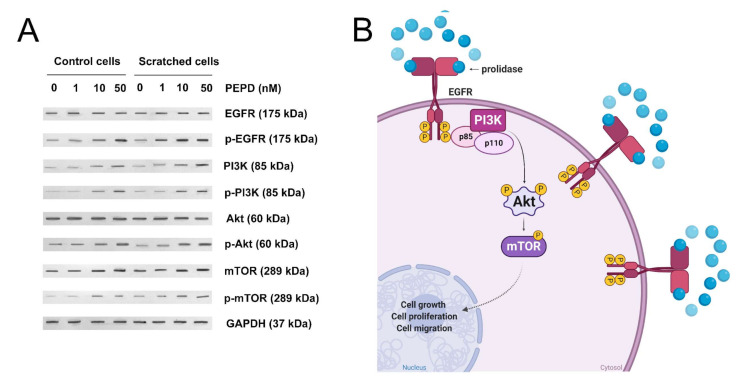
Prolidase induces EGFR-downstream signaling pathway. (**A**) The proteins of the EGFR-downstream signaling pathway analyzed by Western immunoblotting in lysates of control and scratched prolidase-stimulated HaCaT cells. GAPDH was used as a loading control. Representative blot images are shown (densitometric analysis of presented protein is included in Supplementary Materials (Supplementary Figure S3)). (**B**) An illustration of the prolidase-dependent EGFR-downstream signaling pathway activation. Created with BioRender.com.

To establish whether prolidase-dependent functions undergo through EGFR, the expression of downstream signaling proteins was measured in prolidase-treated keratinocytes. As shown in Figure 4A, in prolidase-treated cells (1–50 nM, 24 h) the expressions of the total forms of EGFR, PI3K (p85), Akt, mTOR proteins were increased in a dose-dependent manner. To prove that prolidase activates EGFR in the HaCaT cell line under normal and scratch conditions, Western immunoblotting was applied to evaluate the phosphorylated forms of all aforementioned signaling proteins. As can be seen in Figure 4A, HaCaT cells treated with prolidase (1–50 nM, 30 min) caused phosphorylation of EGFR (Tyr1068) and downstream molecules including PI3K p85 (Tyr458)/p55 (Tyr199), Akt (Ser473), and mTOR (Ser2448). Figure 4B presents a schematic illustration of the PEPD-dependent stimulation of the EGFR-downstream signaling pathway.

**Figure 5:**
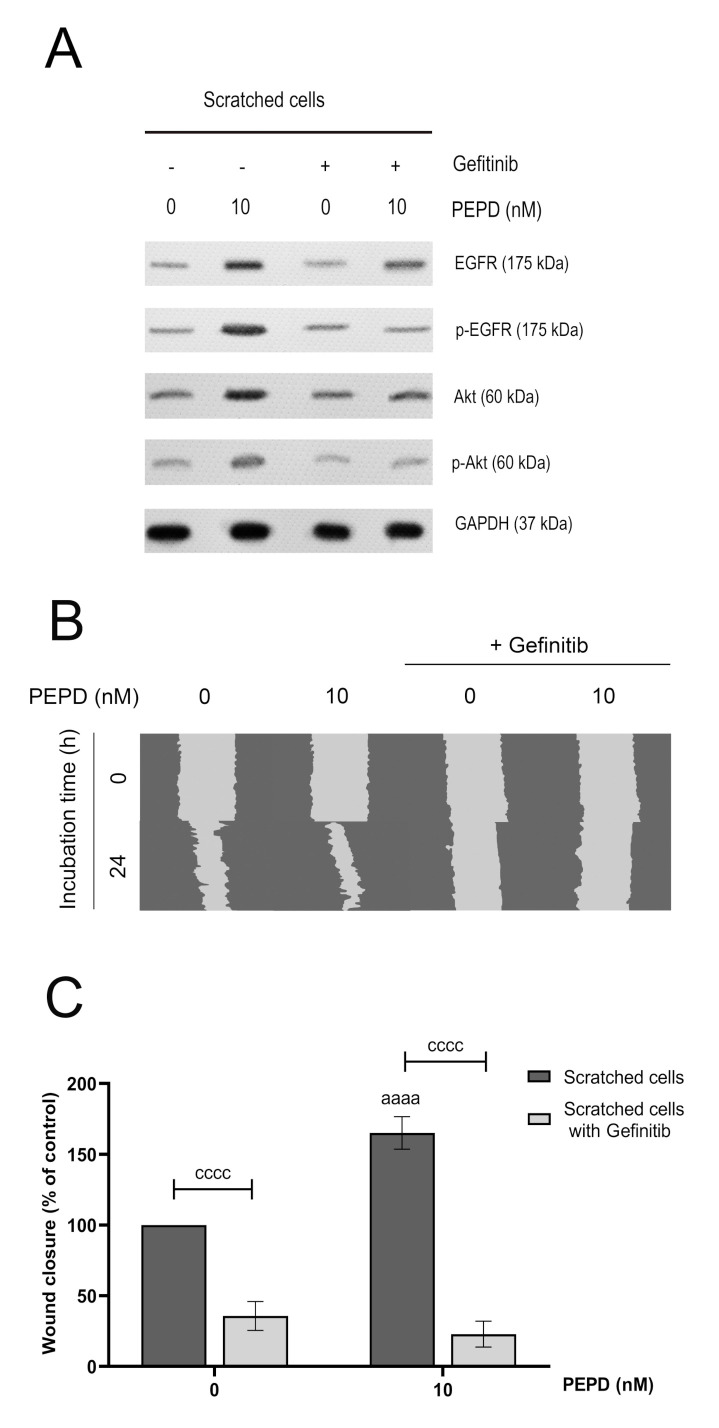
Prolidase elicits migration in HaCaT cells through EGFR. (**A**) The level of expression of EGFR and Akt proteins analyzed by Western immunoblotting in lysates of scratched HaCaT cells incubated with PEPD (10 nM) and an inhibitor of EGFR (Gefitinib, 2 µM) for 30 min and 24 h. Representative blot images are shown (densitometric analysis of presented protein is included in Supplementary Materials (Supplementary Figure S4)). (**B**) Prolidase- and Gefitinib-treated keratinocytes were scratched and monitored using an inverted microscope at 0 and 24 h of incubation. Supplementary Materials contain microscopic images in triplicates (Supplementary Figure S5). (**C**) The wound closure rate of scratched HaCaT cells was evaluated by ImageJ software. The results represent mean ± SEM of three replicates and are significant *aaaa*, or *cccc* < 0.0001 and are marked as *a* vs. control (0 nM of PEPD) of scratched cells, *b* vs. control (0 nM of PEPD) of scratched cells after treated with Gefitinib, and *c* marked scratched cells vs. scratched cells after treated with Gefitinib in the same concentration of PEPD, respectively.

Prolidase-induced EGFR signaling was confirmed by an experiment showing that blocking EGFR abolished PEPD-dependent effects. The cells pretreated with Gefitinib (2 µM, 2 h), a specific EGFR inhibitor, suppressed PEPD-induced EGFR-downstream signaling. The inhibitor strongly diminished PEPD-related EGFR and Akt phosphorylation and the number of total proteins of EGFR and Akt (Figure 5A). Subsequently, we tested cell migration in the presence of prolidase and Gefitinib to investigate the effect of prolidase on migratory ability upon blockade of EGFR. We observed that HaCaT cells pretreated with the EGFR inhibitor and then supplemented with prolidase (10 nM) lost their capacity to migrate (Figure 5B). Quantification of the scratch area demonstrated that prolidase-treated HaCaT cells remained unchanged compared to control after 24 h (Figure 5C).

### 2.6. PI3K/Akt/mTOR Signaling Pathway is Involved in Prolidase-related Proliferation and Migration of HaCaT Keratinocytes

**Figure 6:**
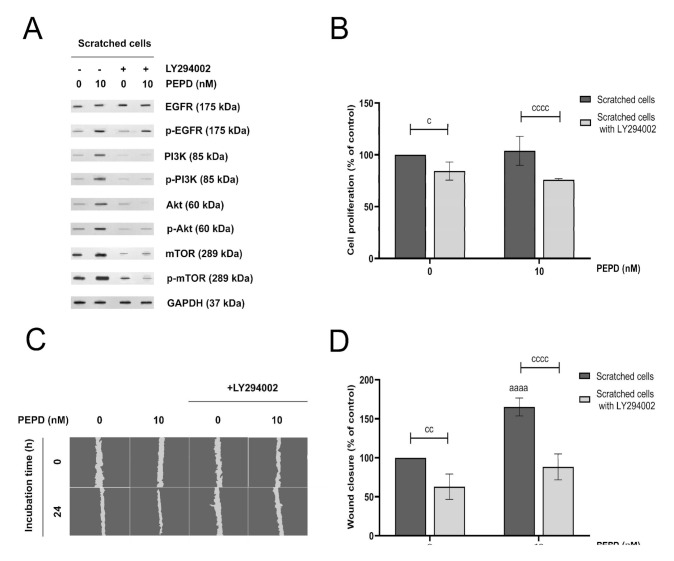
PI3K/Akt/mTOR signaling pathway is involved in prolidase-mediated proliferation and migration of HaCaT cells. (**A**) EGFR-downstream signaling pathway proteins analyzed by Western immunoblotting in lysates of scratched HaCaT cells incubated with prolidase (10 nM) and an inhibitor of PI3K (LY294002) for 30 min and 24 h. Representative blot images are shown (densitometric analysis of presented protein is included in Supplementary Materials (Supplementary Figure S6)). (**B**) Cell proliferation of scratched HaCaT cells incubated with prolidase and LY294002 for 24 h. (**C**) Prolidase- and LY294002-treated keratinocytes were scratched and monitored using an inverted microscope at 0 and 24 h of incubation. Supplementary Materials contain microscopic images in triplicates (Supplementary Figure S7). (**D**) Prolidase and LY294002-stimulated HaCaT cell migration was calculated using ImageJ software and presented as a percent of scratched control cells. A mean ± SEM of three replicates is presented. The results are significant at *cc* < 0.01, and *aaaa*, or *cccc* < 0.0001 and are marked as *a* vs. control (0 nM of PEPD) of scratched cells, *b* vs. control (0 nM of PEPD) of scratched cells pretreated with LY294002, and *c* marked scratched cells vs. scratched cells pretreated with LY294002 at the same concentration of PEPD, respectively.

The PI3K/Akt/mTOR signaling pathway is involved in the proliferation and migration of keratinocytes [21]. To demonstrate the role of prolidase-induced PI3K/Akt/mTOR signaling pathway in these processes during wound healing the HaCaT cells were pretreated with the specific PI3K inhibitor, LY294002 (50 μM, 2 h). The expression of both total and phosphorylated forms of EGFR, PI3K, Akt, and mTOR was evaluated. Figure 6A shows that the inhibition of this pathway by LY294002 noticeably abolished prolidase-induced intracellular signaling through PI3K, Akt, and mTOR. To test the functional impact of the blockade of this pathway, proliferation and migration of keratinocytes were measured. Although in prolidase-treated keratinocytes proliferation of the cells was not much stimulated (Figure 6B), the migration of the cells was increased as it is demonstrated in Figure 6C,D.

### 2.7. Prolidase Induces Expression of the β_1_-integrin Receptor, IGF-1R, and EGFR in Keratinocytes

**Figure 7:**
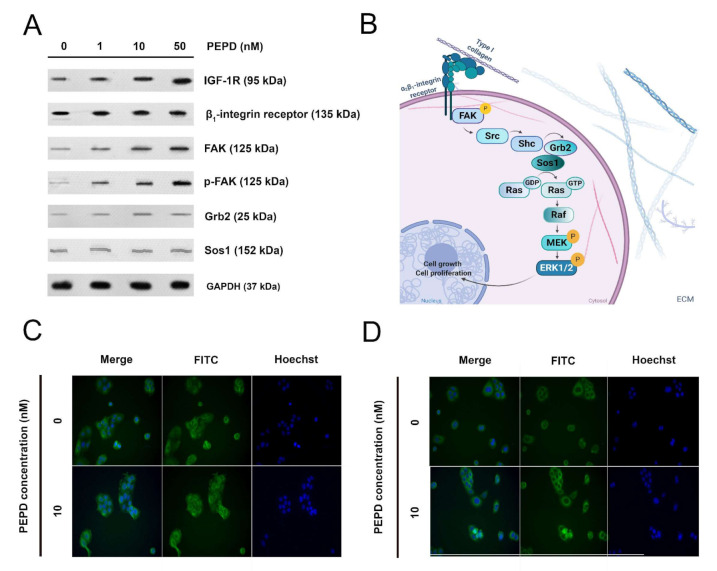
Prolidase stimulates expression of the β_1_-Integrin receptor, IGF-1R, and EGFR signaling proteins. (**A**) The proteins of the β_1_-integrin receptor, IGF-1R, and EGFR-downstream signaling pathways were analyzed by Western immunoblotting in lysates of PEPD-stimulated HaCaT cells for 30 min and 24 h. Representative blot images are shown (densitometric analysis of presented protein is included in Supplementary Materials (Supplementary Figure S8)). (**B**) The scheme for activation of the β_1_-integrin receptor-downstream signaling pathway. Created with BioRender.com. (**C**) Immunostaining of EGFR in PEPD-stimulated HaCaT cells for 24 h. (**D**) Immunostaining of FAK in PEPD-stimulated HaCaT cells for 24 h (magnification 200×).

Prolidase stimulated HaCaT cells to express the β_1_-integrin receptor and insulin-like growth factor 1 receptor (IGF-1R). Activation of the β_1_-integrin receptor and IGF-1R was accompanied by an increase in the expression of downstream proteins such as FAK, Grb2, and Sos1 (Figure 7A). Phosphorylation of FAK (Tyr397) was detected under prolidase treatment. The potential signaling pathway, which mediates Ras/Raf/ERK signaling is illustrated in Figure 7B. Immunofluorescence analysis confirmed an increase in the expression of EGFR upon prolidase stimulation in a model of scratched cells (Figure 7C). Moreover, it proved that FAK was stimulated in mechanically damaged keratinocytes subjected to prolidase treatment (Figure 7D).

### 2.8. Extracellular Prolidase Affects Epithelial-to-Mesenchymal Transition in HaCaT Cells

**Figure 8:**
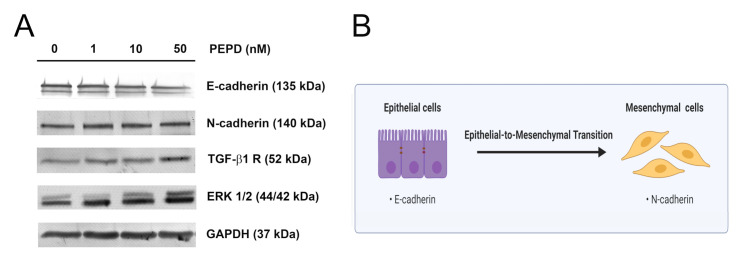
Extracellular prolidase affects epithelial-to-mesenchymal transition in HaCaT cells. (**A**) The proteins of the EMT process were analyzed by Western immunoblotting in lysates of PEPD-stimulated HaCaT cells for 24 h. Representative blot images are shown (densitometric analysis of presented protein is included in Supplementary Materials (Supplementary Figure S9)). (**B**) An illustration of the idea for the epithelial-to-mesenchymal transition process. Created with BioRender.com.

In a scratching experiment, we observed that prolidase enhanced the ability of keratinocytes to migrate. Thus, we tested whether PEPD is involved in the promotion of the epithelial-to-mesenchymal transition (EMT) process. We evaluated the effect of prolidase on this process at the protein level under conditions of mechanical damage in cultured HaCaT cells. Using Western immunoblotting, we found that the levels of expression of key EMT markers such as E-cadherin and N-cadherin were slightly downregulated and upregulated, respectively (Figure 8A). It is known that non-canonical activation of the TGF-β_1_ receptor also mediates EMT through MAPK signaling including ERK1/2 and p38 [22]. To examine whether this pathway was affected by prolidase, we performed Western immunoblotting for the TGF-β_1_ receptor (TGF-β_1_R) and ERK1/2. We found that the levels of expression of TGF-β_1_R and ERK1/2 were upregulated, compared to control cells indicating that EMT occurred. As can be seen in Figure 8B, loss of E-cadherin and the gain of N-cadherin facilitates the migratory phenotype of cells promoting their migration. E-cadherin is involved in the formation of tight cell–cell junctions, thus their lost may result in increased keratinocyte migration.

### 2.9. Extracellular Prolidase Stimulates Collagen Biosynthesis in HaCaT Cells

**Figure 9:**
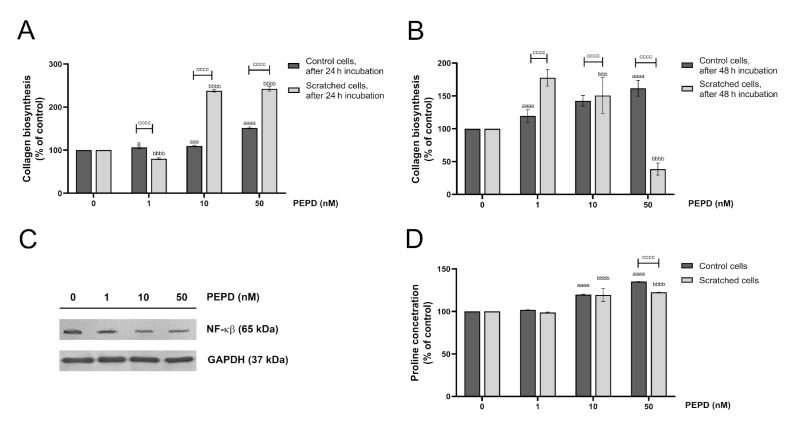
Extracellular prolidase stimulates collagen biosynthesis in HaCaT cells via an increase in proline synthesis and a downregulation of the level of NF-ĸβ. (**A**,**B**) Collagen biosynthesis was measured in prolidase-treated HaCaT cells (1–50 nM) after 24 and 48 h. (**C**) Analysis of NF-ĸβ in the cell lysate of keratinocytes upon PEPD supplementation after 24 h using Western immunoblotting. Representative blot images are shown (densitometric analysis of presented protein is included in Supplementary Materials (Supplementary Figure S10). (**D**) LC–MS-based analysis of proline concentration in prolidase-treated HaCaT cells after 24 h incubation. The results represent a mean ± SEM of three replicates are significant at *a* = < 0.05, *aaa*, or *bbb*, < 0.001, and *aaaa*, *bbbb,* or *cccc* < 0.0001 and are marked as *a* vs. control (0 nM of PEPD) of unscratched (control) cells, *b* vs. control (0 nM of PEPD) of scratched cells, and *c* marked unscratched (control) cells vs. scratched cells in the same concentration of PEPD, respectively.

The last step of the wound healing process comprises extracellular matrix (ECM) remodeling in which collagen is required to restore connective tissue [19]. 5-[3H]-proline incorporation assay was employed to study the effect of prolidase on collagen biosynthesis in HaCaT keratinocytes. The cells were treated with different concentrations of prolidase (1–50 nM) for 24 and 48 h. As shown in Figure 9A,B, prolidase stimulated collagen biosynthesis in control and wounded keratinocytes in a dose-dependent manner. When prolidase was added to cultured cells for 24 h, the rate of collagen biosynthesis was 2-fold higher in a model of wounded cells than in normal HaCaT cells. Complementary Western immunoblotting shows that prolidase inhibited expression of NF-ĸβ suggesting the mechanism for prolidase-dependent collagen generation (Figure 9C). In the collagen molecule, proline constitutes about 10% of total amino acids [23]. The LC–MS-based analysis revealed that prolidase treatment of HaCaT in a dose-dependent manner increased proline concentration both in control and scratched cells (Figure 9D).

## 3. Discussion

To the best of our knowledge, this study represents the first investigation of the functional significance of prolidase-dependent stimulation of EGFR signaling in a model of wounded keratinocytes. Based on the original observation by Yang et al. [3] showing that prolidase directly binds to and activates EGFR, we hypothesized that extracellular prolidase may promote cell migration and proliferation facilitating wound repair. Impaired wound healing is observed in numerous conditions such as acute and chronic diseases, aging, or after surgery [24] thus searching for new boosters of tissue regeneration is crucial.

In our study, we found that prolidase in a dose-and time-dependent manner induced keratinocyte proliferation and migration both in control and “wounded” cells. However, these processes were more pronounced in “wounded” cells. The difference cannot be attributed to the vitality of the cells since no changes in this parameter were found. We observed that prolidase, added to the culture medium of human keratinocytes, activated EGFR downstream signaling through PI3K/Akt/mTOR proteins. Activated EGFR induces PI3K/Akt/mTOR and Ras/Raf/ERK pathways [12]. As PEPD does not share an EGF motif with other EGFR ligands [3], we investigated the potency of this protein to phosphorylate EGFR. EGF is known as the most potent inducer of EGFR. Kinetic analysis showed that the prolidase in a time-dependent manner phosphorylated EGFR and its downstream molecules, similarly to that of EGF suggesting that prolidase can elicit sustained phosphorylation of EGFR-downstream signaling proteins.

We found that PEPD significantly improved cell migration to close the scratch area in a model of wound healing in vitro. Under normal conditions, during re-epithelialization, keratinocytes are attracted to the injury site to restore tissue continuity [19]. Given the fact that they evoke epithelial characteristics such as low migratory ability, the epithelial-to-mesenchymal transition has to occur. Here, we found that PEPD promoted the migration of HaCaT cells suggesting that EMT happened. We observed the upregulation of key EMT markers such as N-cadherin and downregulation of E-cadherin. As induction of TGF-β_1_ receptor through canonical (Smad signaling) and non-canonical (MAPK signaling) pathways also mediate EMT [22], we found that the amount of TGF-β_1_R and ERK1/2 were increased which indicates EMT process in HaCaT cells. Activation of non-canonical TGF- β_1_R signaling during EMT in keratinocytes was also presented in previous studies [22,25]. Our study demonstrated that PEPD could activate keratinocytes and stimulate cell migration through the EMT process facilitating wound healing.

Further, we applied an EGFR inhibitor, Gefitinib, to prove that prolidase binds to EGFR on the surface of keratinocytes. Inhibition of prolidase-dependent EGFR activation by Gefitinib led to a decrease in the amount of phospho- and total forms of EGFR and Akt confirming that prolidase is a ligand of this receptor. Then, we found that prolidase-mediated wound closure rate was abolished in the presence of Gefitinib supporting that activation of PEPD driven EGFR is a crucial event in HaCaT cell migration. Similar results presented previously showing that EGFR inhibition caused a reduction of keratinocyte migration [26]. Subsequently, to establish whether PI3K/Akt/mTOR is functionally linked to the proliferation and migration of HaCaT cells, we used a pharmacological inhibitor of PI3K, LY294002. Blocking this signaling pathway resulted in noticeable inhibition of the amount of phospho- and total forms of EGFR-downstream proteins. HaCaT cells were pretreated with LY294002 and then subjected to prolidase treatment followed by cell proliferation and migration assays. LY294002-pretreated cells exhibited decreased cell proliferation and migration. The results showed that the prolidase-stimulated PI3K/Akt/mTOR pathway is functionally required for the proliferation and migration of human keratinocytes. Diminished proliferation and migration of keratinocytes after inhibition of the PI3K/Akt/mTOR pathway were also observed previously by Lee et al. [27].

Moreover, prolidase was found to activate the expressions of the β_1_-integrin receptor and insulin-like growth factor 1 receptor (IGF-1R). It is of great importance in wound healing since the role of both receptors in anabolic processes is well established [28]. Their role is particularly important in collagen biosynthesis. Both, the β_1_-integrin and IGF-1 receptors transmit signals that induce collagen biosynthesis [10,13]. This process is of critical importance in the last step of wound healing and scar formation [28]. Upon stimulation, the β_1_-integrin receptor induces autophosphorylation of FAK, which is then capable of interaction with Grb2, through Src and Shc proteins and then further cascade of signaling pathway through Sos, Ras, and Raf proteins and subsequently two MAP kinases: ERK1 and ERK2 [15,16]. The end-point of the signaling cascade is the induction of transcription factors that stimulate the expression of genes for several proteins involved in the regulation of cell growth, differentiation, and metabolism [17]. Performing analysis of the expression of the selected proteins from this pathway we confirmed prolidase-dependent stimulation of the β_1_-integrin receptor and its downstream proteins. We demonstrated that prolidase-stimulated HaCaT cells enhanced collagen biosynthesis as a result of increased expression of β_1_-integrin and IGF-1R receptors. Our results are supported by the observation made by Pappas et al. [29] who established that the ERK1/2 level reflects the rate of collagen synthesis. Moreover, we demonstrated that NF-ĸβ expression was decreased under prolidase treatment in keratinocytes. Since NF-ĸβ is known as an inhibitor of expression of α1 and α2 subunits of type I collagen [6,7,30]. It suggests that a drop in NF-ĸβ expression facilitated collagen biosynthesis. We found that proline content increased in the cells treated with prolidase, providing a substrate for the synthesis of collagen [31]. Prolidase as an enzyme participates in recycling proline from imidodipeptides (mostly derived from degradation products of collagen) for resynthesis of collagen and other proline-containing proteins. However, we found that the intracellular activity of prolidase did not change in prolidase-treated cells. Similar results were reported by Yang’s study [3]. It is likely that in the experimental conditions proline came from the enzymatic reduction of pyrroline-5-carboxylic acid derived from glutamate or ornithine [32].

Given the activation of EGFR and β_1_-integrin receptor signaling by prolidase, it seems that both receptors cooperate by cross-talk mechanism in tissue regeneration processes. Furthermore, overexpression of prolidase resulted in increased nuclear HIF-1α level and elevated expression of HIF-1α-dependent gene products, vascular endothelial growth factor (VEGF), and glucose transporter-1 (Glut-1) [5,21,23]. Since HIF-1α, EGFR, and Glut-1 are involved in angiogenesis (as one of the tissues regeneration steps), regulation of prolidase expression and its activity could also play a major role in wound healing [33,34]. It is well established that platelet-rich plasma (PRP) facilitates the tissue repair process. Due to the high content of prolidase and growth factors contained in PRP, it has been widely used in regenerative medicine, especially in acute and chronic soft tissue injuries [28,35,36,37,38]. Poor wound healing is usually accompanied by impaired clot formation that limits access of all constituents of blood to the injured cells.

The functional significance of prolidase was found in prolidase deficiency (PD). Mutations in the prolidase gene are the molecular basis for PD resulting in decreased enzymatic activity of PEPD. It has been found several mutated alleles of the PEPD gene [39,40,41]. PD is a rare autosomal recessive disorder characterized by massive imidodipeptiduria, skin lesions, and elevated proline-containing dipeptides in plasma [42,43,44,45,46,47,48]. The most specific symptom of PD is reflected by defects in connective tissue metabolism. All symptomatic cases had skin lesions as diffuse telangiectasia, purpuric rash, crusting erythematous dermatitis, or progressive ulcerative dermatitis, particularly on the lower legs. To date, it is believed that PD results from low or a lack of PEPD activity, however, it cannot be excluded that the symptoms result mainly from deficiency of extracellular function of prolidase since supplementation of PD patients with proline or proline-convertible amino acids was unsuccessful in the treatment of the disease [49]. The biological activity of prolidase is of emerging research interest. So far, it is known that prolidase serves as a regulator of p53 function, affects interferon-α/β receptor maturation, and is a ligand of EGFR and epidermal growth factor receptor 2 (HER2) [20]. As our study demonstrated promising effects of prolidase in cell proliferation, migration, and collagen biosynthesis, further investigations are necessary to explore its role in PD.

## 4. Materials and Methods

### 4.1. HaCaT Cell Cultures

HaCaT cells were purchased from Cell Line Service (Eppelheim, Germany) and cultured in DMEM cell culture medium (PanBiotech, Germany) supplemented with 10% fetal bovine serum (FBS; Gibco, Carlsbad, CA, USA) and 1% penicillin/streptomycin (Gibco, Carlsbad, CA, USA) at 37 °C in a humidified atmosphere of 5% CO_2_. The medium was replaced every 3 days until 80% of confluency. For different applications, cells were seeded on various culture dishes (Sarstedt, Nümbrecht, Germany). For Western immunoblotting, cells were cultured on 100-mm dishes at a density of 2 × 10^6^ cells/plate. To evaluate cell vitality, collagen biosynthesis, prolidase activity, and proline concentration HaCaT cells were cultured on 6-well plates at 2 × 10^5^ cells/well. For wound healing assay, cells were plated at a density of 1 × 10^5^ cells/well in 12-well dishes. For proliferation assay and immunocytochemistry, cells were seeded at 5 × 10^3^ cells/well in a 96-well plate.

### 4.2. HaCaT Treatment

The cells (5–8th passages) were treated with prolidase from the porcine kidney (Sigma Aldrich, Saint Louis, MO, USA) at the concentrations of 1–50 nM. The enzyme, delivered as a lyophilized powder, was reconstituted in sterile phosphate buffer (PBS; pH 7.4, PanBiotech, Germany). For wound healing, proliferation, and Western immunoblotting HaCaT cells were pretreated with LY294002, selective PI3K inhibitor (Cell Signaling Technology, Danvers, MA, USA) at the working concentration of 50 μM for 2 h before treatment with prolidase (1–50 nM, 30 min and 24 h). Keratinocytes were subjected to pretreatment with Gefitinib (Sigma Aldrich, Saint Louis, MO, USA), an EGFR inhibitor at the working concentration of 2 µM for 2 h before treatment with prolidase (1–50 nM, 3 h and 24 h). The cells pretreated with Gefitinib were analyzed by Western immunoblotting and wound healing assay.

### 4.3. Cell Proliferation Assay

The proliferation of HaCaT cells was evaluated using commercially available CyQUANT^®^ Cell Proliferation Assay (Thermo Fisher Scientific, Waltham, MA, USA). HaCaT cells were submitted to prolidase treatment at concentrations of 1–50 nM, and EGF (10 nM, Gibco, Carlsbad, CA, USA) as a positive control for 24–48 h. After incubation, cells were rinsed twice with PBS (pH 7.4) and frozen at −80 °C until analysis. Before analysis, samples were thawed at RT, and 200 μL of the mixture consisted of CyQUANT^®^ GR dye/cell-lysis buffer was added and incubated for 5 min at RT. The plate was protected from light. Fluorescence was read on Victor X4 Multilabel Reader (PerkinElmer, Waltham, MA, USA) at 480 nm and 520 nm as excitation and emission wavelengths, respectively. The results were presented as the percent of the control value.

### 4.4. Cell Vitality Assay

The cell vitality was assessed by measurement of the level of intracellular thiols. After incubation with prolidase (1–50 nM, 24 h) or EGF (10 nM) as a positive control, cells were washed twice with warm PBS (pH 7.4) and trypsinized (0.25%). Then, cells were centrifuged (5 min, 500× *g*), the pellet was washed with PBS and stained with Solution 5 (ChemoMetec, Lillerød, Denmark) containing VitaBright-48, acridine orange, and propidium iodide and analyzed using Nucleo Counter NC-3000 (ChemoMetec, Lillerød, Denmark). The results were presented as the percent of the control value.

### 4.5. Preparation of Lysates

The cells were cultured with prolidase (1–50 nM) for 30 min and 24 h. Before harvesting cells were rinsed twice with cold PBS (pH 7.4). Then, cells were scraped in RIPA lysis buffer (Thermo Fisher Scientific, Waltham, MA, USA) with protease inhibitor (cOmplete™ Protease Inhibitor Cocktail, Roche, Basel, Switzerland) and phosphatase inhibitor cocktail (PhosSTOP, Roche, Basel, Switzerland) and incubated on ice for 15 min. Lysates were sonicated three times (15 sec on and 5 sec off) followed by centrifugation at 4 °C (10 min, 12,000× *g*). The supernatant was transferred to a fresh tube and stored at −80 °C until Western immunoblotting. Protein concentration was measured using the Pierce BCA assay kit (Thermo Fisher Scientific, Waltham, MA, USA).

### 4.6. Western Immunoblotting

For Western immunoblotting, equal amounts (5–20 µg/lane) of protein were diluted in RIPA lysis buffer (Thermo Fisher Scientific, Waltham, MA, USA) and mixed with Laemmli buffer (120 mM Tris-HCl, 20% glycerol, 0.4% SDS, and 0.02% bromophenol blue, pH 6.8) containing fresh 5% β-mercaptoethanol (Sigma Aldrich, Saint Louis, MO, USA). The samples were denatured at 95 °C for 7 min. The proteins were separated on 7.5–10% SDS-PAGE gels and then blotted onto polyvinylidene difluoride (PVDF; BioRad Laboratories, Hercules, CA, USA) membranes. The membranes were blocked with either 5% non-fat dried milk (Santa Cruz Biotechnology, Dallas, TX, USA) or BSA (Sigma Aldrich, Saint Louis, MO, USA) in TBS-T (20 mM Tris, 150 mM NaCl, 0.1% Tween-20, pH 7.6) for 1 h at room temperature with agitation. The membranes were incubated with primary antibodies overnight at 4 °C, followed by incubation with alkaline phosphatase-linked goat antirabbit or antimouse antibodies for 1 h at RT. The membranes were washed three times in TBS-T for 5 min. The bands were visualized using 1-Step™ NBT/BCIP Substrate Solution (Thermo Fisher Scientific, Waltham, MA, USA) and their intensities were semiquantitatively measured with ImageJ software (https://imagej.nih.gov/ij/). All experiments were run in triplicates.

### 4.7. Antibodies

The membranes were incubated with the following primary antibodies purchased from Cell Signaling Technology (Danvers, MA, USA): p44/42 MAPK (ERK1/2) Rabbit mAb (1:1000), mTOR Rabbit mAb (1:1000), EGF Receptor Rabbit mAb (1:1000), IGF-1 Receptor β Rabbit mAb (1:1000), phospho-EGF Receptor (Tyr1068) Rabbit mAb (1:1000), phospho-p44/42 MAPK (Thr202/Tyr204) Rabbit mAb (1:1000), phospho-mTOR (Ser2448) Rabbit mAb (1:1000), FAK Rabbit mAb (1:1000), phospho-FAK (Tyr397) Rabbit mAb (1:1000), Integrin β_1_ Receptor Rabbit mAb (1:2000), Akt Rabbit mAb (1:2000), phospho-Akt (Ser473) Rabbit mAb (1:2000), PI3 Kinase p85 Rabbit mAb (1:1000), phospho-PI3 Kinase p85 (Tyr458)/p55 (Tyr199) Antibody (1:1000), NF-κβ p65 Rabbit Antibody (1:1000), TGF-β Receptor I Rabbit Antibody (1:1000), E-Cadherin Rabbit mAb (1:1000), N-Cadherin Rabbit mAb (1:1000), and GAPDH Rabbit mAb (1:1000). Mouse anti-Grb2 (1:500) and mouse anti-Sos1 (1:1000) were purchased from Becton Dickinson (Franklin Lakes, NJ, USA). Secondary alkaline phosphatase-conjugated antimouse or antirabbit antibodies diluted 1:10 000 were from Sigma Aldrich (Saint Louis, MO, USA).

### 4.8. Evaluation of Collagen Biosynthesis

Collagen biosynthesis was determined by the incorporation of radioactive 5-[3H]-proline (5 μCi/mL; Hartmann Analytic, Germany) into proteins susceptible to bacterial collagenase according to the Peterkofsky’s method [50]. After 24 and 48 h incubation, cells were washed with PBS (pH 7.4), harvested in PBS containing 10 mM proline, and frozen at −80 °C until the day of analysis. For collagen digestion, we used purified *Clostridium histolyticum* collagenase (Sigma Aldrich, Saint Louis, MO, USA). The radiometric analysis was performed applying Liquid Scintillation Analyzer Tri-Carb 2810 TR (PerkinElmer, Waltham, MA, USA). The results were normalized to total protein biosynthesis and were presented as a percent of the control value.

### 4.9. Determination of Prolidase Activity

The activity of prolidase was determined according to the method published by Besio et al. [51]. Protein concentration was measured using the Pierce™ BCA Protein Assay Kit (Thermo Fisher Scientific, Waltham, MA, USA). An equal amount (50 µg) of proteins was mixed with 50 mM Tris–HCl (pH 7.8) containing 1 mM MnCl2 and 0.75 mM glutathione and incubated for 1 h at 50 °C. Then, we added 100 mM glycyl-proline (substrate for prolidase) and incubated for 30 min at 50 °C. The reaction was stopped by 0.45 M ice-cold TCA. After centrifugation (15 min, 12,000× *g*), the supernatant was transferred to fresh tubes and incubated with Chinard’s reagent (12 min, 90 °C) followed by incubation on ice for 15 min. Absorbance was read at 515 nm on TECAN Infinite^®^ M200 PRO (Männedorf, Switzerland). The results were reported as a percent of the control value.

### 4.10. In Vitro Wound Healing Assay

At confluence, HaCaT cells were scratched using a sterile 200 μL pipette tip, washed twice with PBS, and incubated with different concentrations of prolidase in 0.5% FBS-containing DMEM for 24 and 48 h. Every 24 h cells were photographed using an inverted optical microscope (Nikon; Minato, Tokyo, Japan) with a 40× magnification for monitoring the wound closure area. The wound closure was measured by ImageJ software (https://imagej.nih.gov/ij/) and its rate was calculated according to the following formula.

$$\mathrm{wound}\;\mathrm{healing}\;\mathrm{rate}=\frac{\mathrm{original}\;\mathrm{wound}\;\mathrm{area}-\mathrm{unhealed}\;\mathrm{wound}\;\mathrm{area}}{\mathrm{original}\;\mathrm{wound}\;\mathrm{area}}$$

All the experiments were performed in triplicates.

### 4.11. Immunofluorescence Staining and Confocal Microscopy

After 24 incubation with prolidase (10 nM), cells were rinsed with prewarmed PBS twice. For fixation, 3.7% paraformaldehyde was used (10 min). Permeabilization with 0.1% Triton for 5 min was performed before FAK and NF-κβ visualization in contrast to that of EGFR where no permeabilization step was conducted. The next step included blocking with 3% fetal horse serum for 60 min at room temperature (RT). Then, cells were incubated with target primary antibodies (anti-EGFR, anti-FAK, and anti-NF-κβ) overnight at 4 °C at dilutions 1:500 and 1:100, 1:400, respectively. As a secondary antibody were used antirabbit FITC-linked antibody (Becton Dickinson, Franklin Lakes, NJ, USA) at a concentration of 5 µg/mL for 1 h in the dark at RT. The cell nuclei were stained with Hoechst (1 ng/mL). Immunofluorescence staining was visualized using a confocal laser scanning microscope (BD Pathway 855 Bioimager, Becton Dickinson, Franklin Lakes, NJ, USA) supported with AttoVision software.

### 4.12. LC–MS-Based Quantitative Analysis

LC–MS analysis of proline concentration in HaCaT cells was conducted according to the method of Klupczynska et al. [52]. Briefly, cells were harvested by scraping in ice-cold methanol with stable isotopically labeled proline (25 µM, d3-proline; Sigma Aldrich, Saint Louis, MO, USA) as an internal standard. Cell lysates were stored at −80 °C until analysis. Samples were analyzed using Agilent 1260 Infinity HPLC system coupled to Agilent 6530 Q-TOF mass spectrometry detector with electrospray ionization (Agilent Technologies, Santa Clara, CA, USA) as an ion source in positive ionization mode. Samples were injected onto a HILIC column (Luna HILIC, 2 mm × 100 mm, 3 µm, Phenomenex, Torrance, CA, USA) thermostated at 30 °C. All samples were randomized before analysis. The results were normalized to protein concentration and presented as a percent of the control value.

### 4.13. Statistical Analysis

All experiments were carried out at least three replicates and the experiments were repeated at least three times. Data are shown as a mean ± standard error (SEM). For statistical calculations, a one-way analysis of variance (ANOVA) with Dunnett’s correction and *t*-test were used. Statistical analysis was performed using GraphPad Prism 5.01 (GraphPad Software, San Diego, USA). Statistically significant differences were marked as *p* < 0.05, *pp* < 0.01, *ppp* < 0.001, and *pppp* < 0.0001 and presented by using letters showing the differences between appropriate controls described in the legend of figures.

## 5. Conclusions

**Figure 10:**
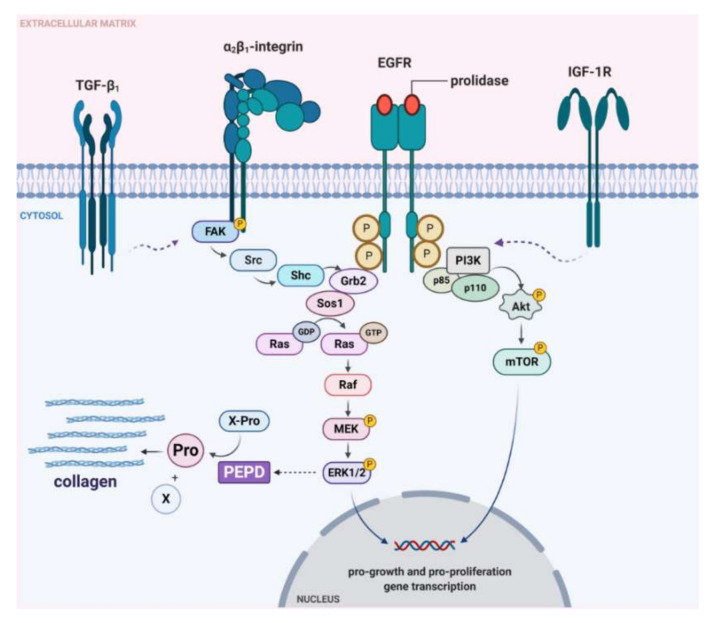
Graphical illustration of prolidase-dependent EGFR, β_1_-integrin receptor, TGF-β_1_, and IGF-1R-downstream signaling. Under experimental conditions of mechanically damaged HaCaT cells, prolidase causes activation of the ERK1/2 and PI3K/Akt/mTOR pathways resulting in increased collagen biosynthesis and cell proliferation and migration. Created with BioRender.com.

The data presented in this report suggest that extracellular prolidase acting through EGFR induced growth, migration, and collagen biosynthesis in cultured keratinocytes (Figure 10). Therefore, prolidase may represent a therapeutic approach to treat skin wounds.

## Supplementary Materials

Supplementary materials can be found at https://www.mdpi.com/1422-0067/21/23/9243/s1.

## Abbreviations

Akt	protein kinase B
EGF	epidermal growth factor
EGFR	epidermal growth factor receptor
ERK1/2	extracellular signal-regulated kinase 1/2
FAK	focal adhesion kinase pp125^FAK^
GAPDH	glyceraldehyde 3-phosphate dehydrogenase
Glut-1	glucose transporter-1
Grb2	growth factor receptor-bound protein 2
HER 2	epidermal growth factor receptor 2
HIF-1α	hypoxia-inducible factor 1 alpha
IGF-1R	insulin-like growth factor 1 receptor
JAK	Janus kinase
LY294002	phosphoinositide 3 kinase inhibitor
MAPK	mitogen-activated protein kinase
MEK	mitogen-activated protein kinase kinase
mTOR	mammalian target of rapamycin
NF-κβ	nuclear factor kappa beta
PD	prolidase deficiency
PEPD	prolidase
PI3K	phosphoinositide 3 kinase
Pro	proline
PRP	platelet-rich plasma
Shc	SHC adaptor protein 1
Sos1	son of sevenless 1
STAT	signal transducer and activator of transcription
Src	proto-oncogene Src
TGF-β_1_R	transforming growth factor-beta 1 receptor
VEGF	vascular endothelial growth factor
