# Supplementary material for: Prolidase Stimulates Proliferation and Migration through Activation of the PI3K/Akt/mTOR Signaling Pathway in Human Keratinocytes
Source: Int J Mol Sci. 2020 Dec 3;21(23):9243. doi: 10.3390/ijms21239243 (PMC7730528; doi:10.3390/ijms21239243)
Supplement: Supplementary file 1 [file ijms-21-09243-s001.pdf]

Supplementary Files

Prolidase Stimulates Proliferation and Migration Through Activation of the PI3K/Akt/mTOR Signaling Pathway in Human Keratinocytes

Magdalena Misiura [1], Weronika Baszanowska [2], Ilona Ościłowska [2], Jerzy Pałka [2] and Wojciech Miltyk [1,*]

[1] Department of Analysis and Bioanalysis of Medicines, Medical University of Bialystok, Jana Kilińskiego 1, 15-089 Bialystok; Poland; magdalena.misiura@umb.edu.pl (M.M.); wojciech.miltyk@umb.edu.pl (W.M.)

[2] Department of Medicinal Chemistry, Medical University of Bialystok, Jana Kilińskiego 1, 15-089 Bialystok, Poland; w.baszanowska22@wp.pl (W.B.); ilona.zareba@gmail.com (I.O.); pal@umb.edu.pl (J.P.)

* Correspondence: wojciech.miltyk@umb.edu.pl; Tel.: +48-85-748-5845

Incubation time – 0 h

PEPD concentration – 0 nM

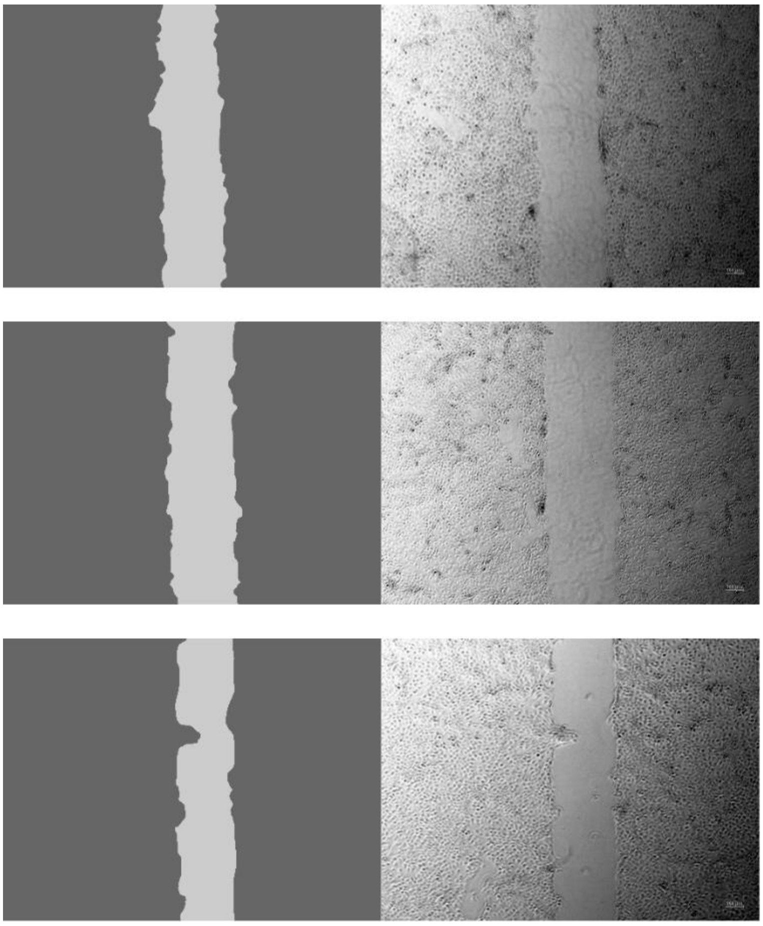

PEPD concentration – 50 nM

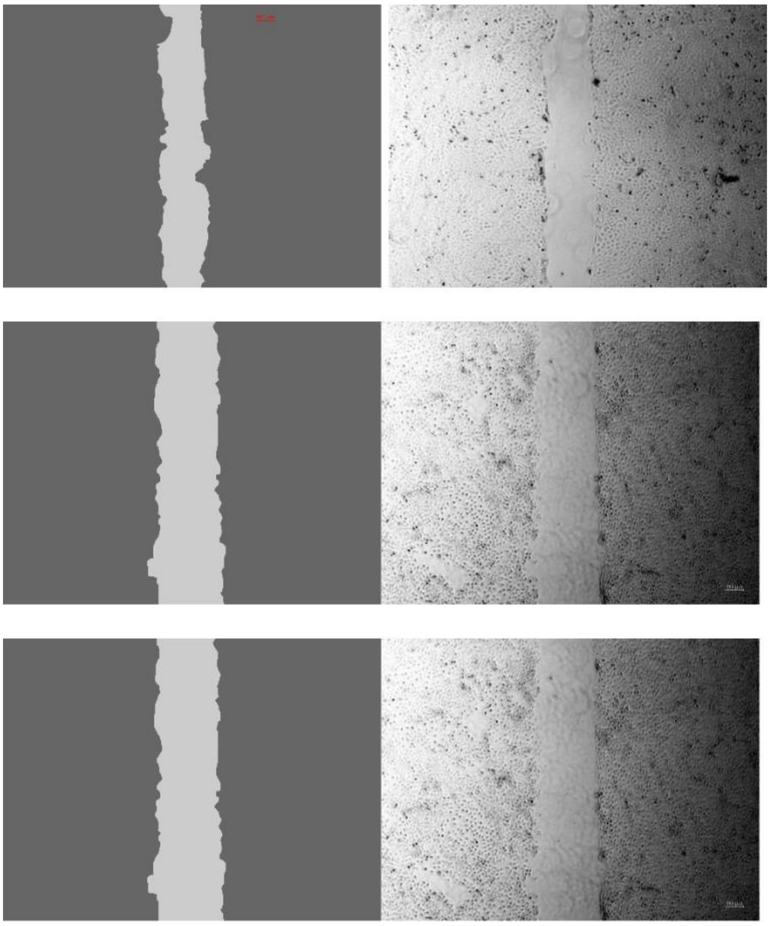

Incubation time – 24 h

PEPD concentration – 0 nM

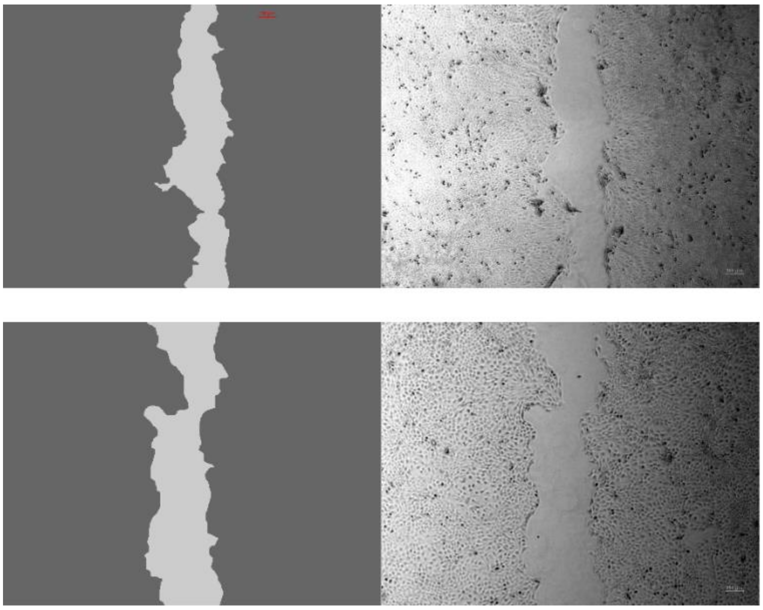

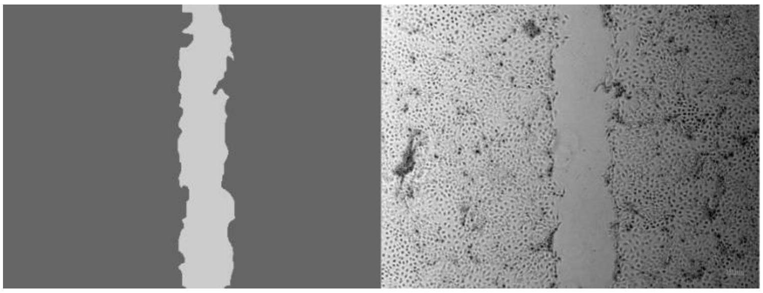

PEPD concentration – 50 nM

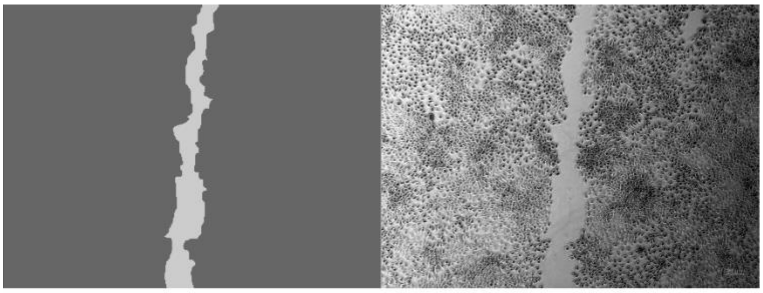

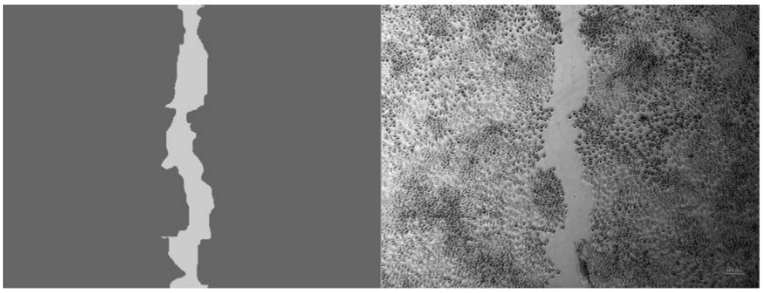

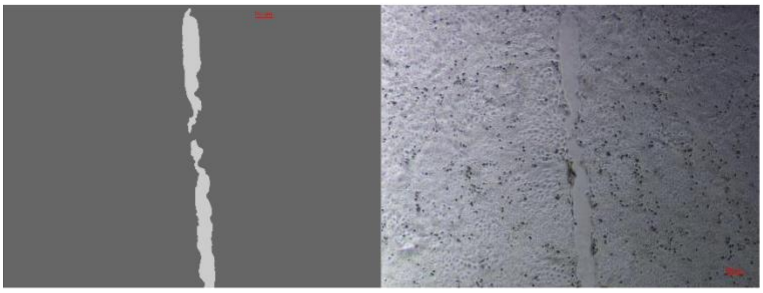

Incubation time – 48 h

PEPD concentration – 0 nM

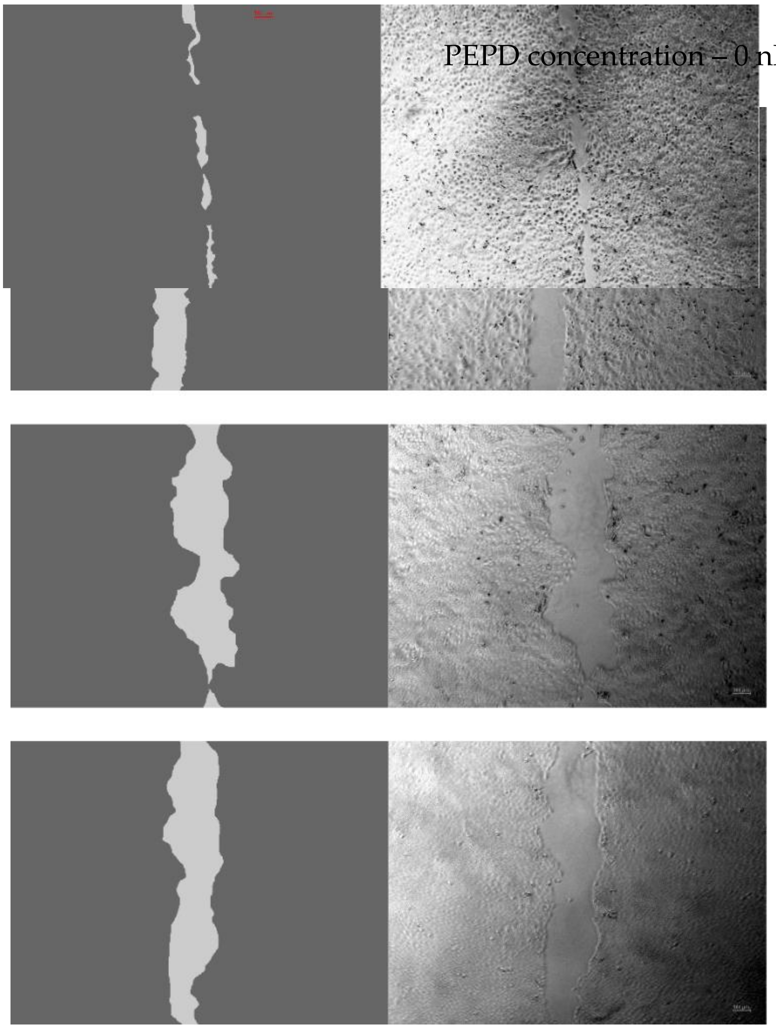

PEPD concentration – 50 nM

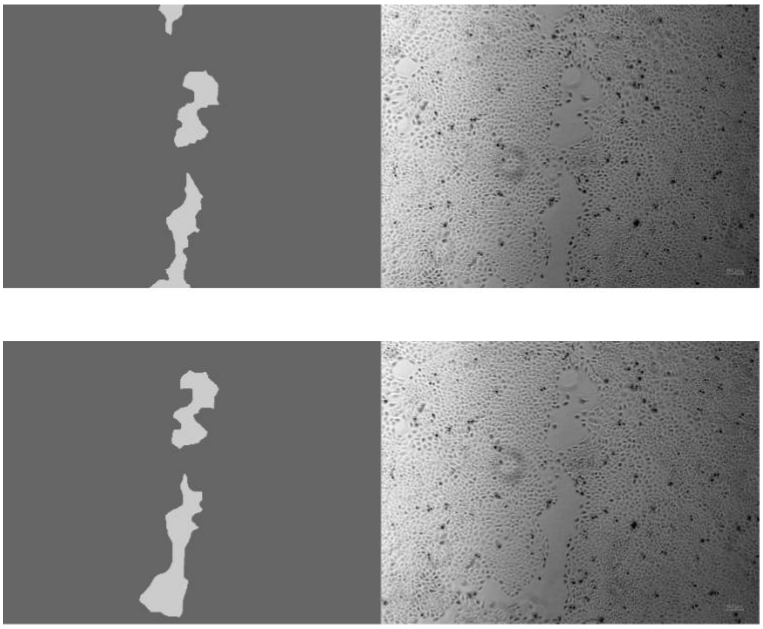

Figure S1. Microscopic images of migration of HaCaT cells presented in Fig. 1B.

EGFR expression and its intensity in EGF-treated HaCaT cells

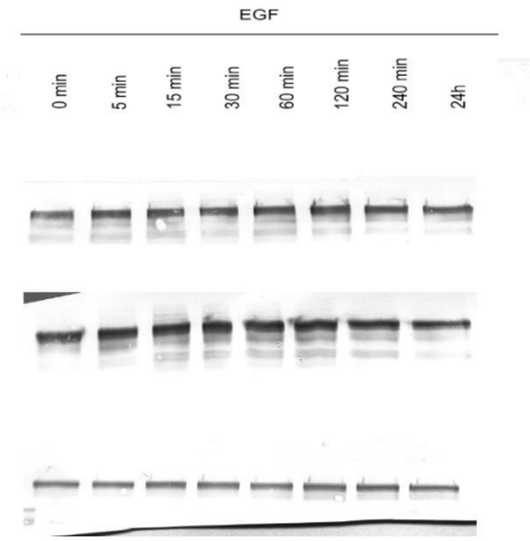

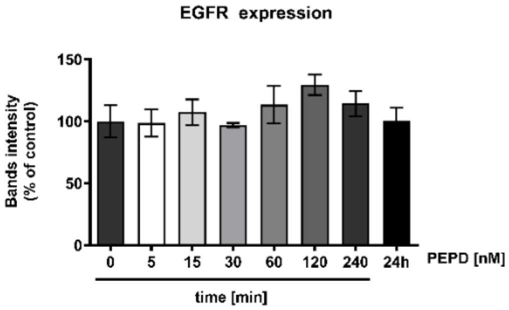

p-EGFR expression and its intensity in EGF-treated HaCaT cells

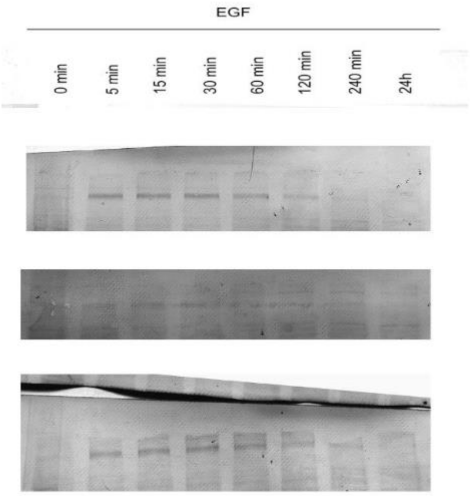

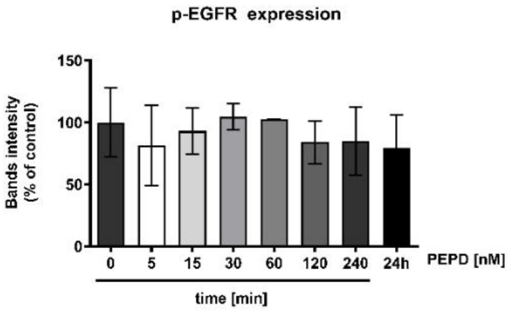

Akt expression and its intensity in EGF-treated HaCaT cells

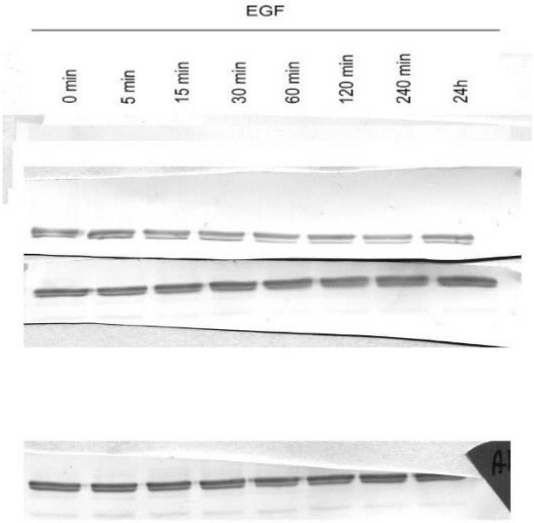

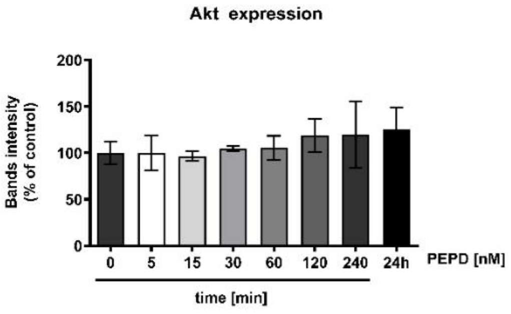

p-Akt expression and its intensity in EGF-treated HaCaT cells

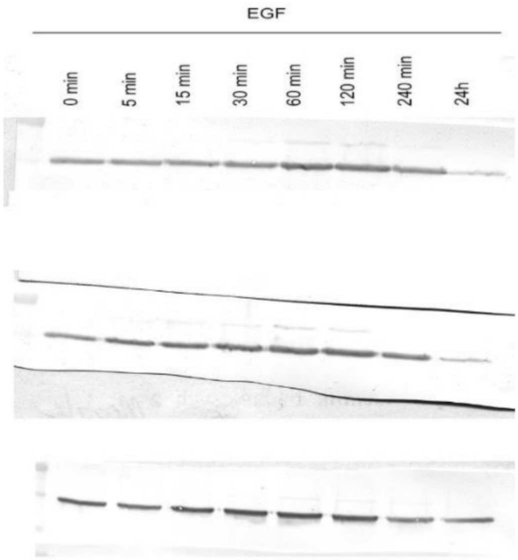

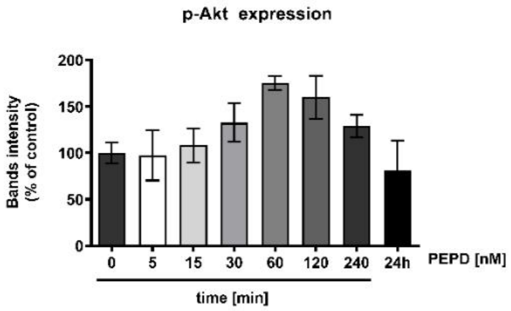

mTOR expression and its intensity in EGF-treated HaCaT cells

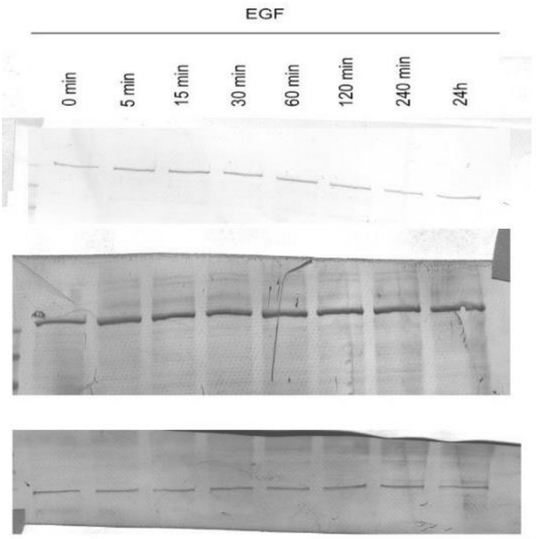

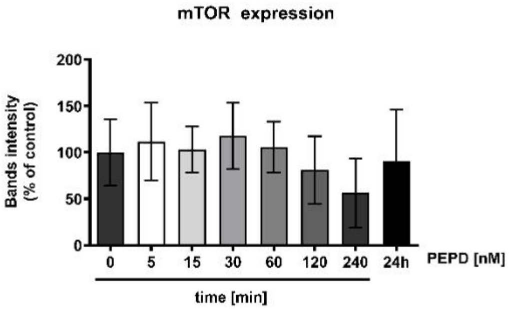

p-mTOR expression and its intensity in EGF-treated HaCaT cells

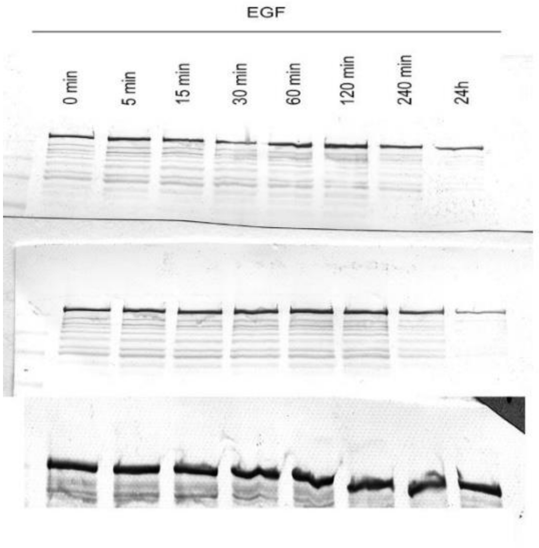

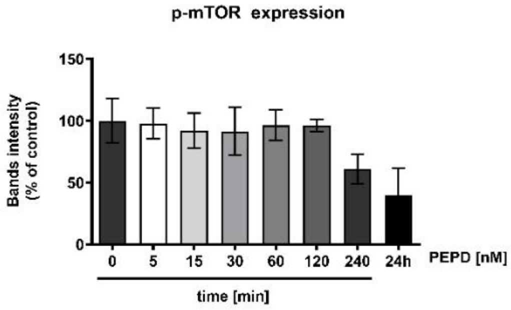

ERK1/2 expression and its intensity in EGF-treated HaCaT cells

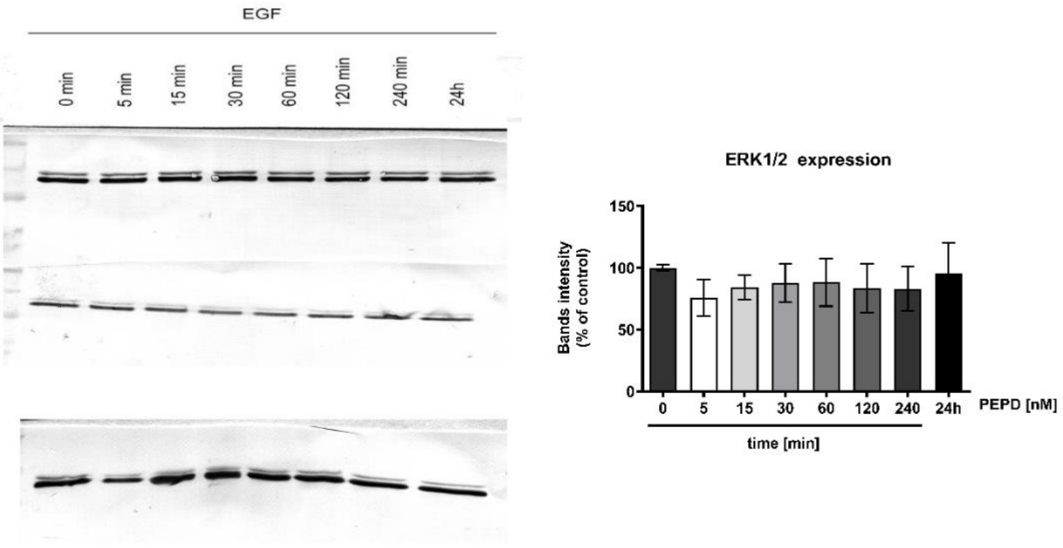

p-ERK1/2 expression and its intensity in EGF-treated HaCaT cells

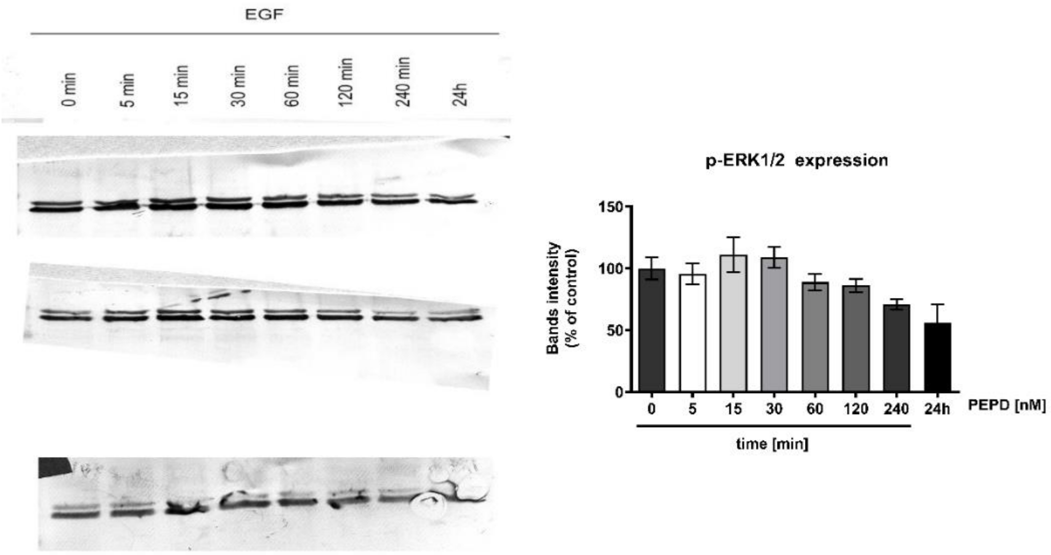

GAPDH expression and its intensity in EGF-treated HaCaT cells

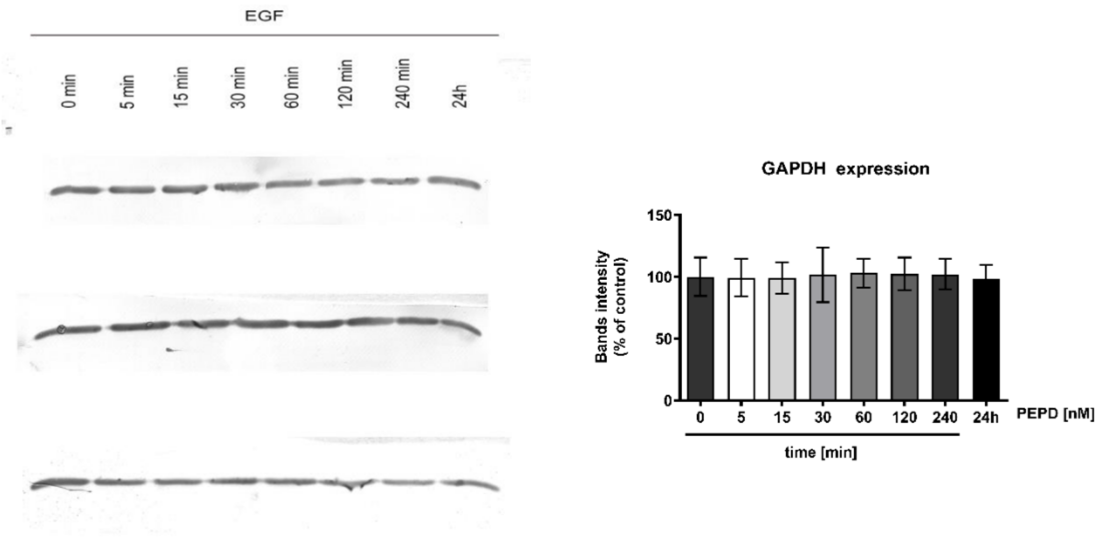

EGFR expression and its intensity in PEPD-treated HaCaT cells

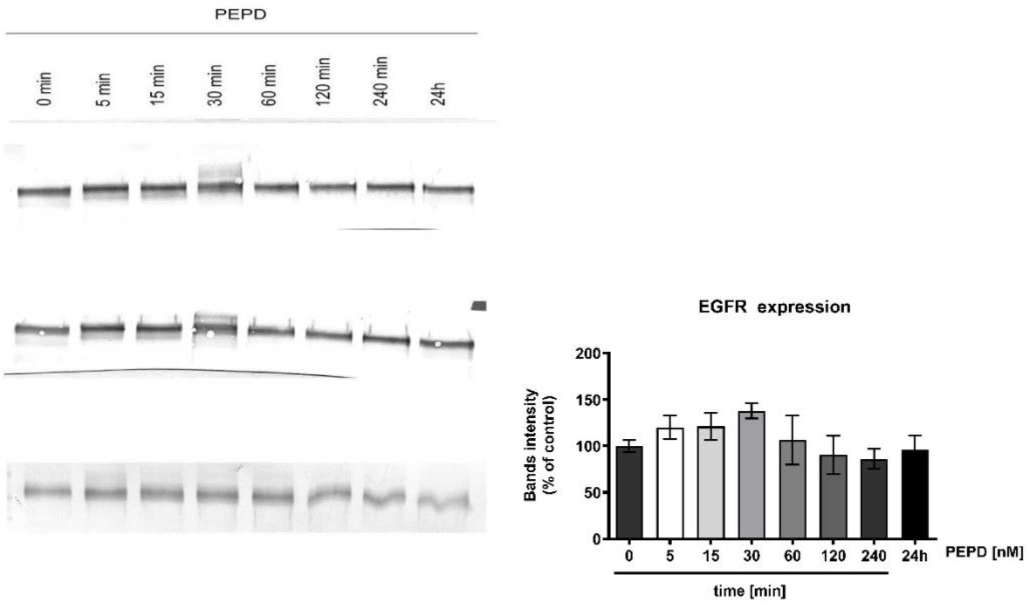

p-EGFR expression and its intensity in PEPD-treated HaCaT cells

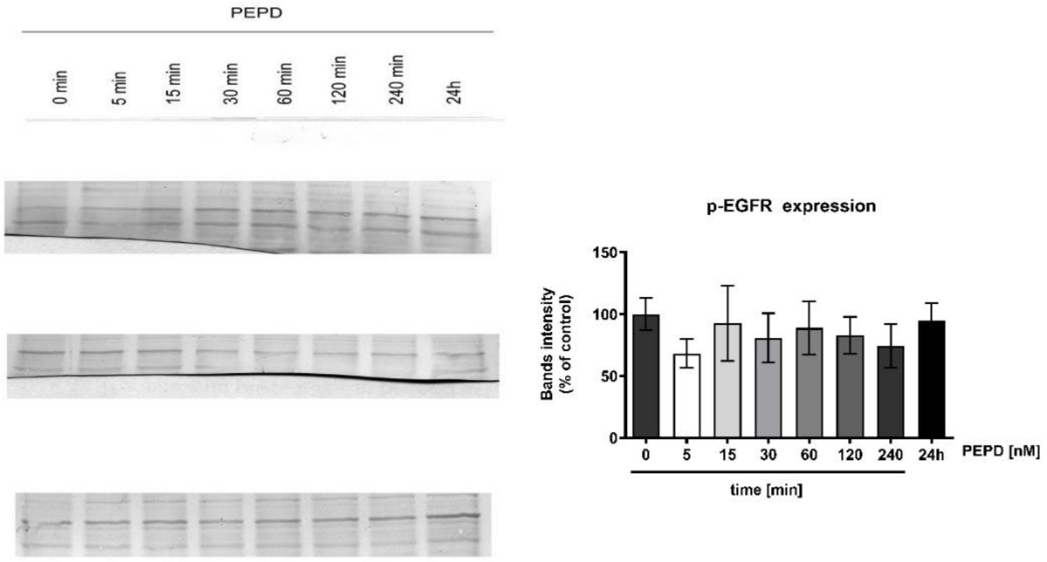

Akt expression and its intensity in PEPD-treated HaCaT cells

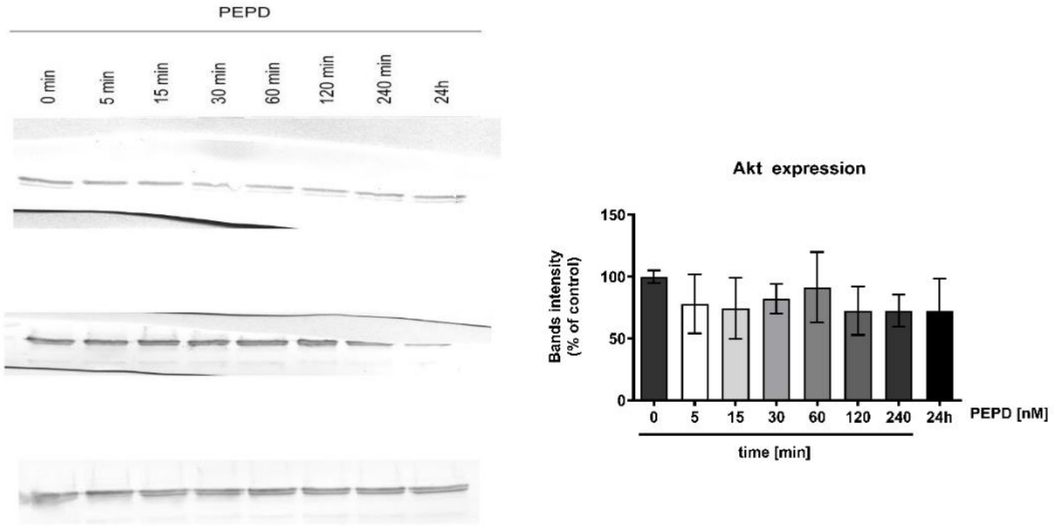

p-Akt expression and its intensity in PEPD-treated HaCaT cells

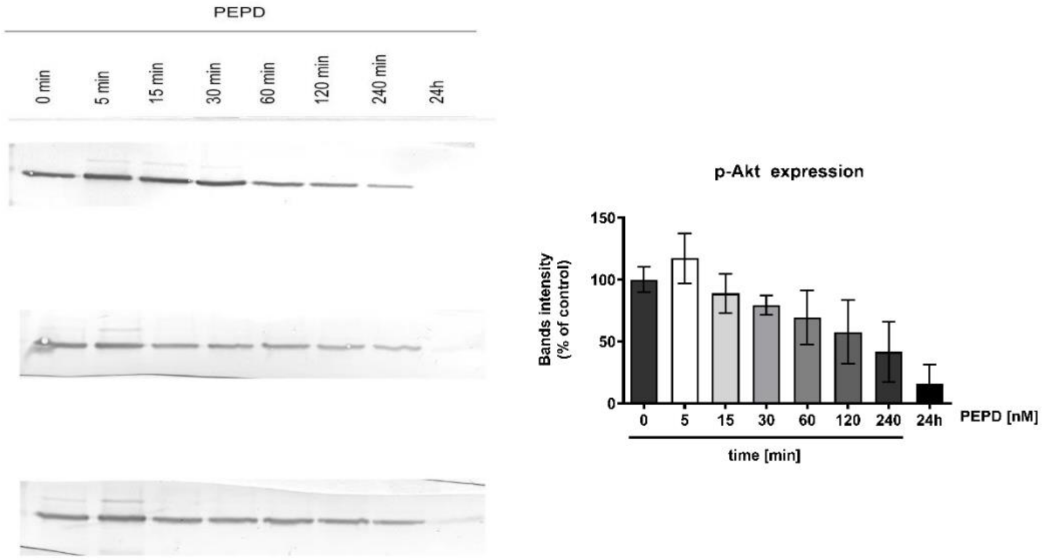

mTOR expression and its intensity in PEPD-treated HaCaT cells

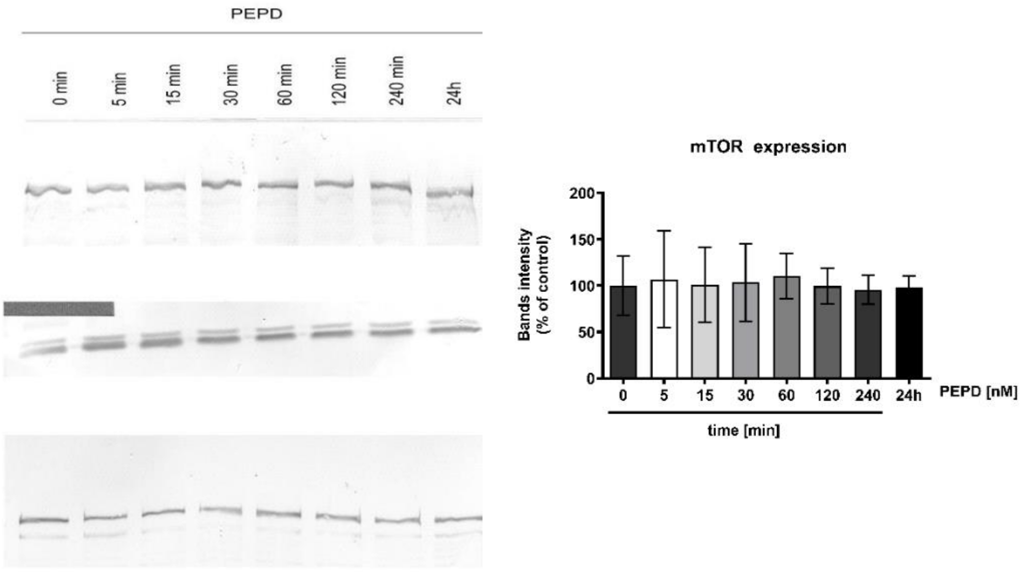

p-mTOR expression and its intensity after PEPD-treated

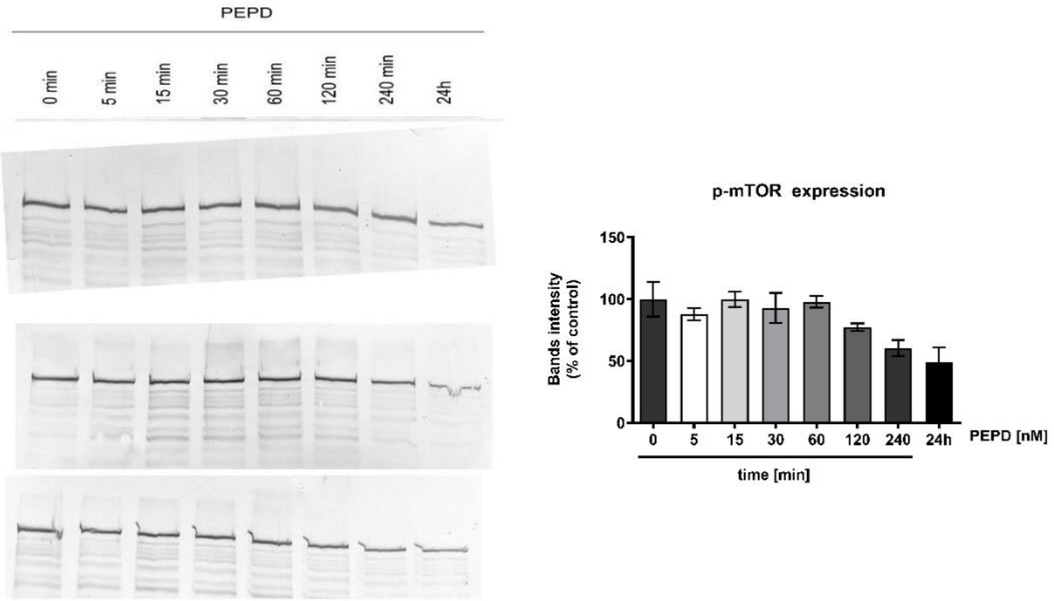

ERK1/2 expression and its intensity after PEPD-treated

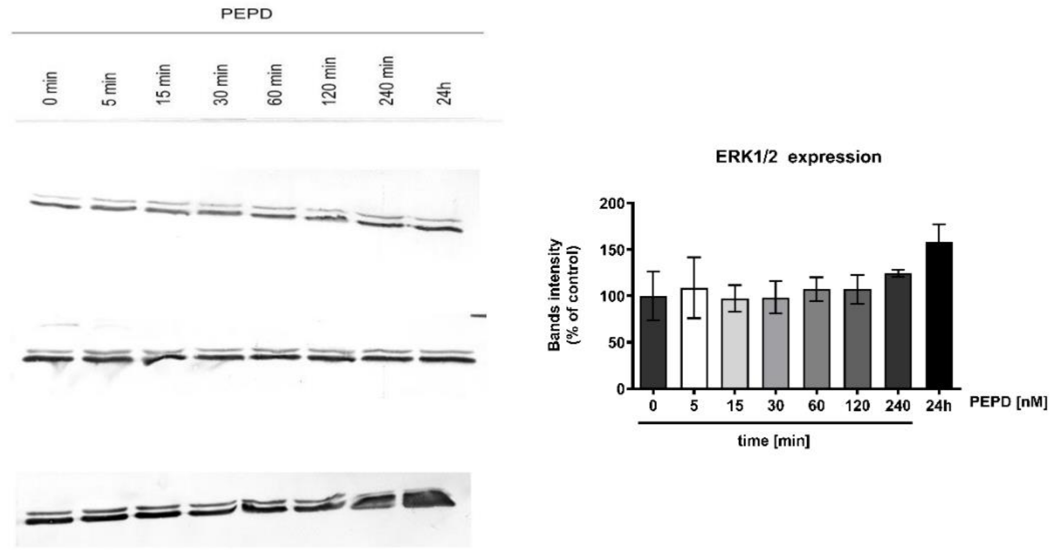

p-ERK1/2 expression and its intensity after PEPD-treated

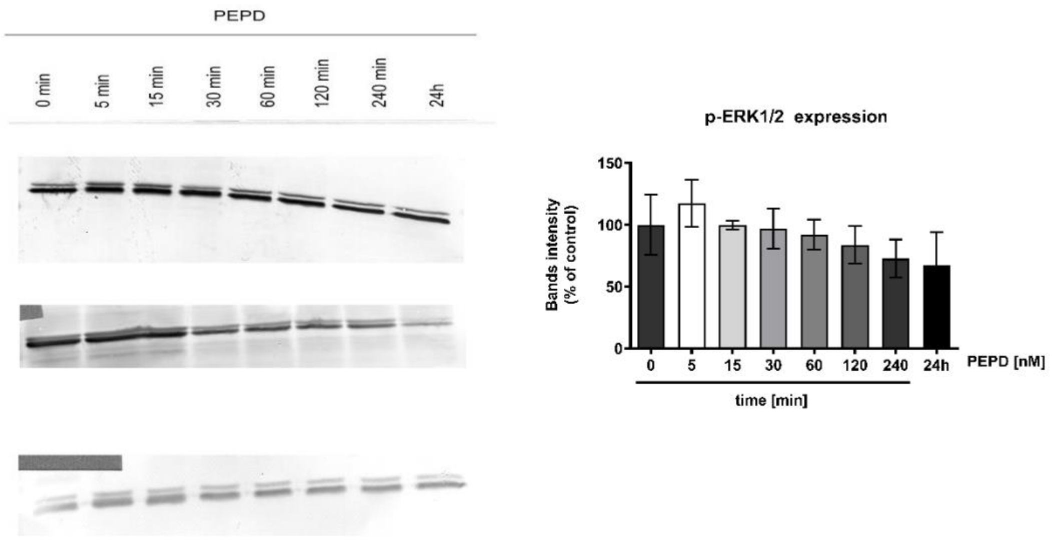

GAPDH expression and its intensity in PEPD-treated HaCaT cells 8

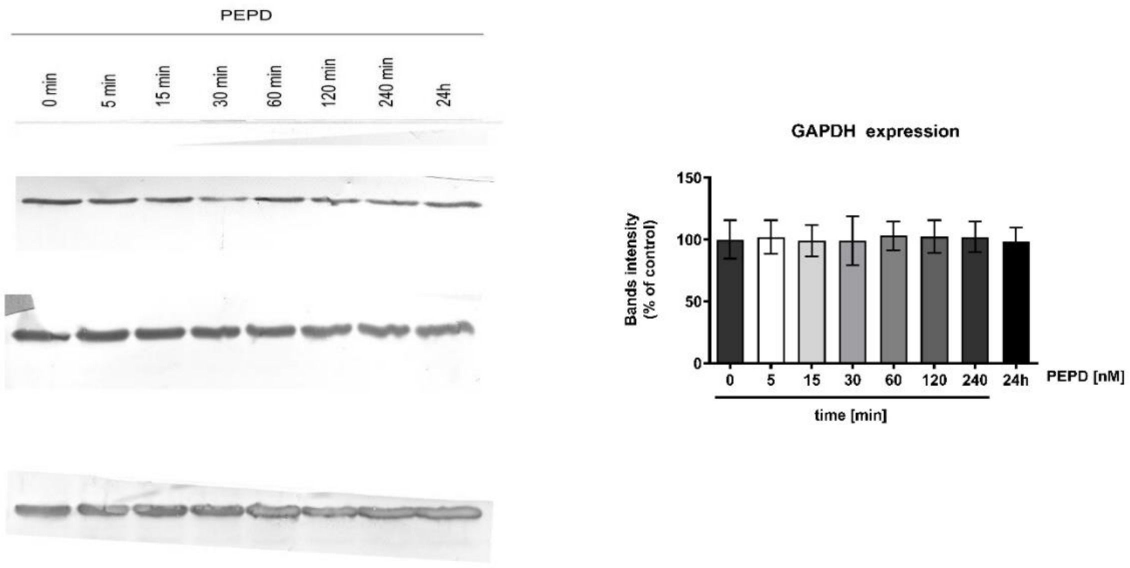

Figure S2. Representative blots from Western blotting analysis presented in Figure 3A and B.

EGFR expression and its intensity

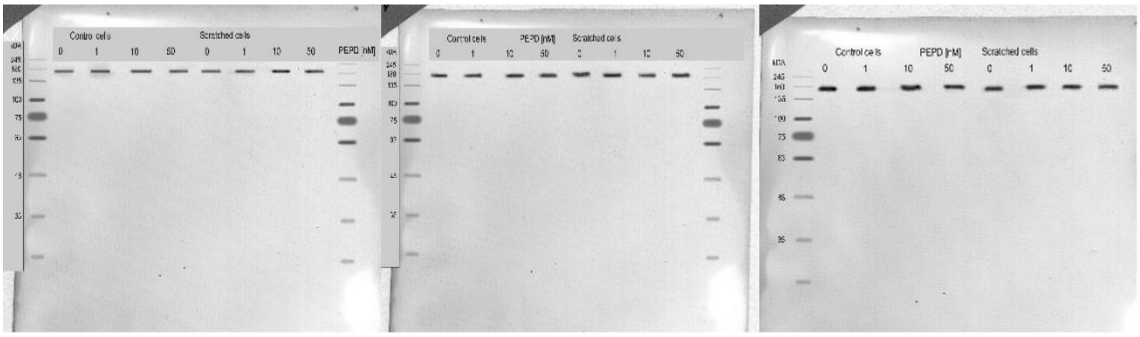

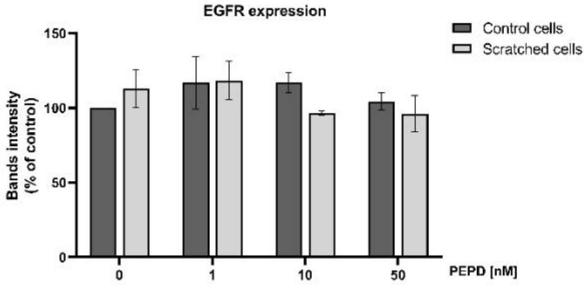

p-EGFR expression and its intensity

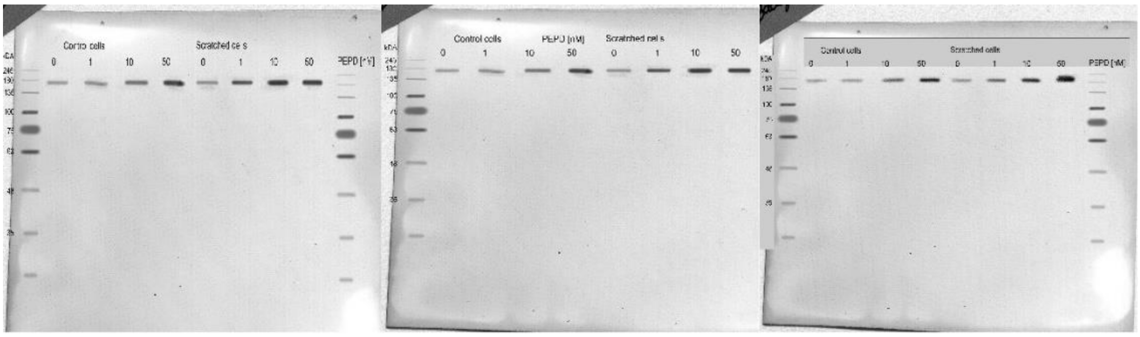

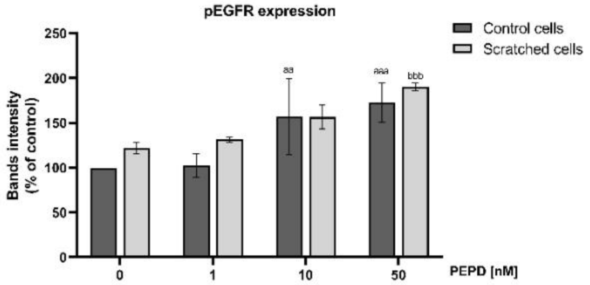

PI3K expression and its intensity

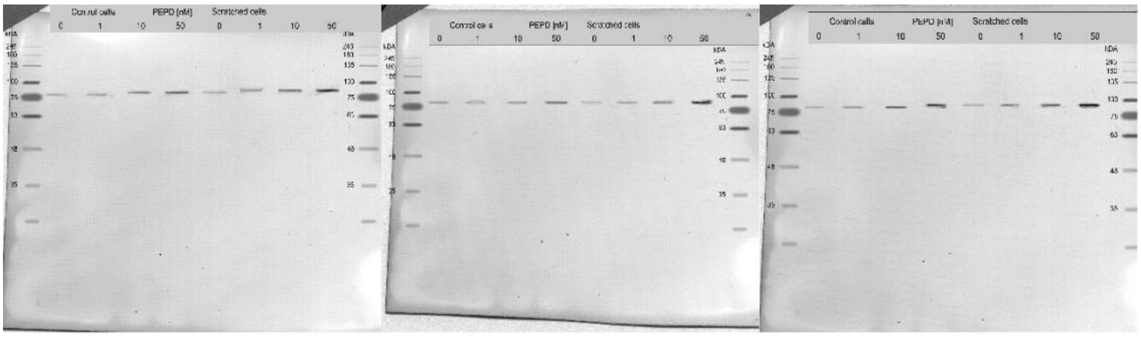

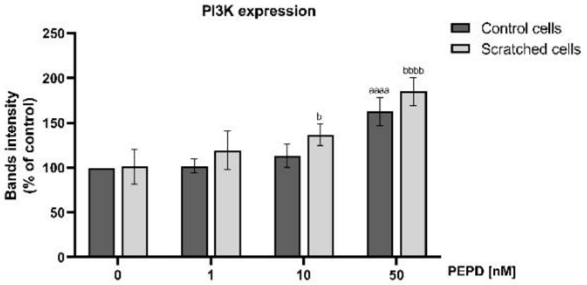

p-PI3K expression and its intensity

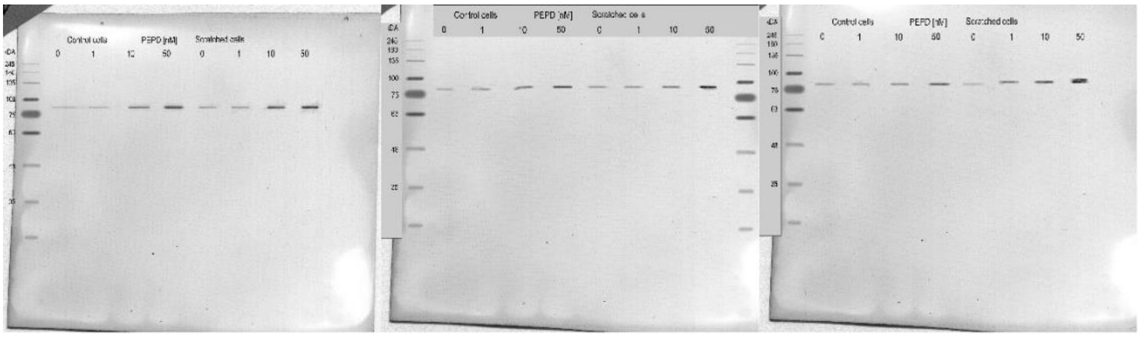

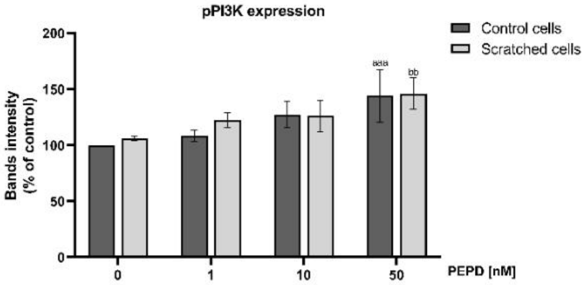

Akt expression and its intensity

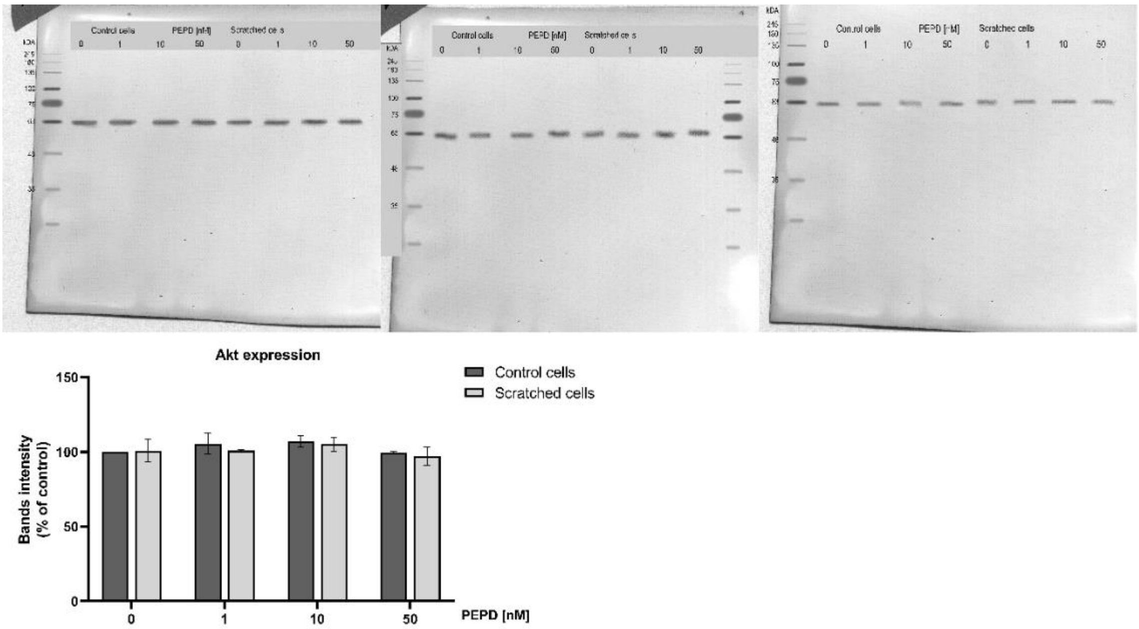

p-Akt expression and its intensity

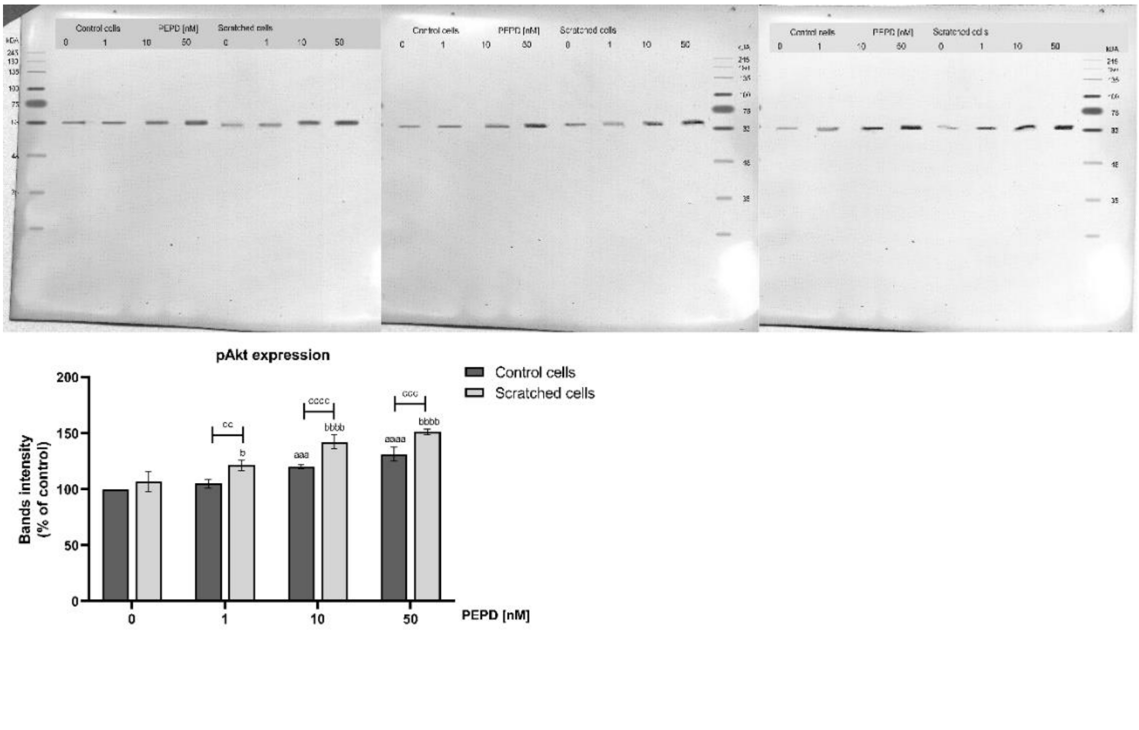

mTOR expression and its intensity

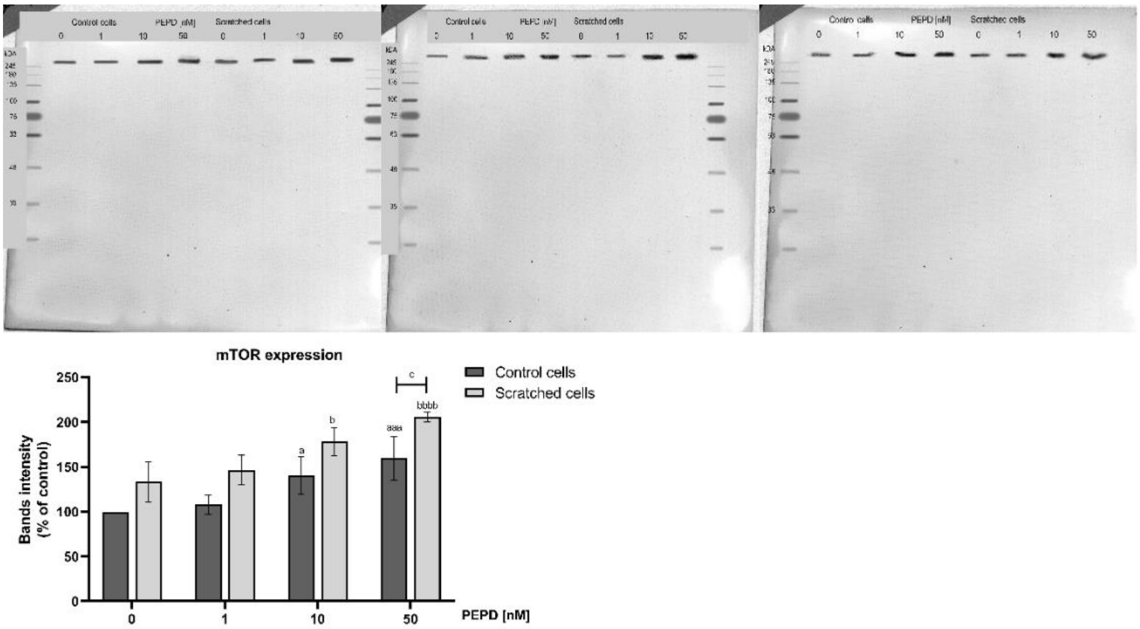

p-mTOR expression and its intensity

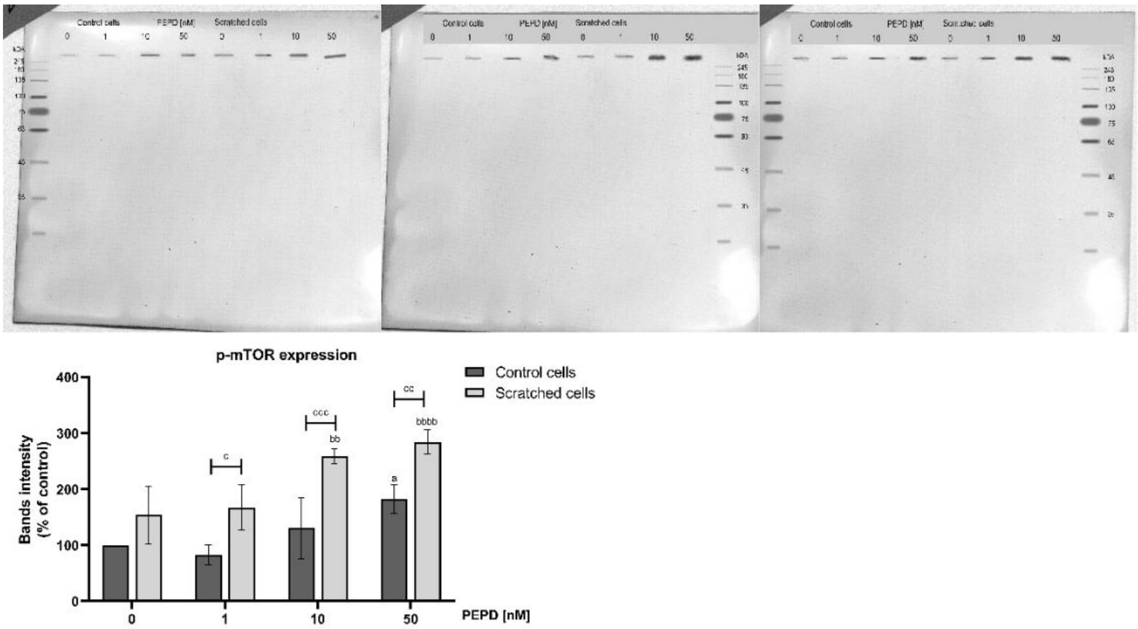

GAPDH expression and its intensity

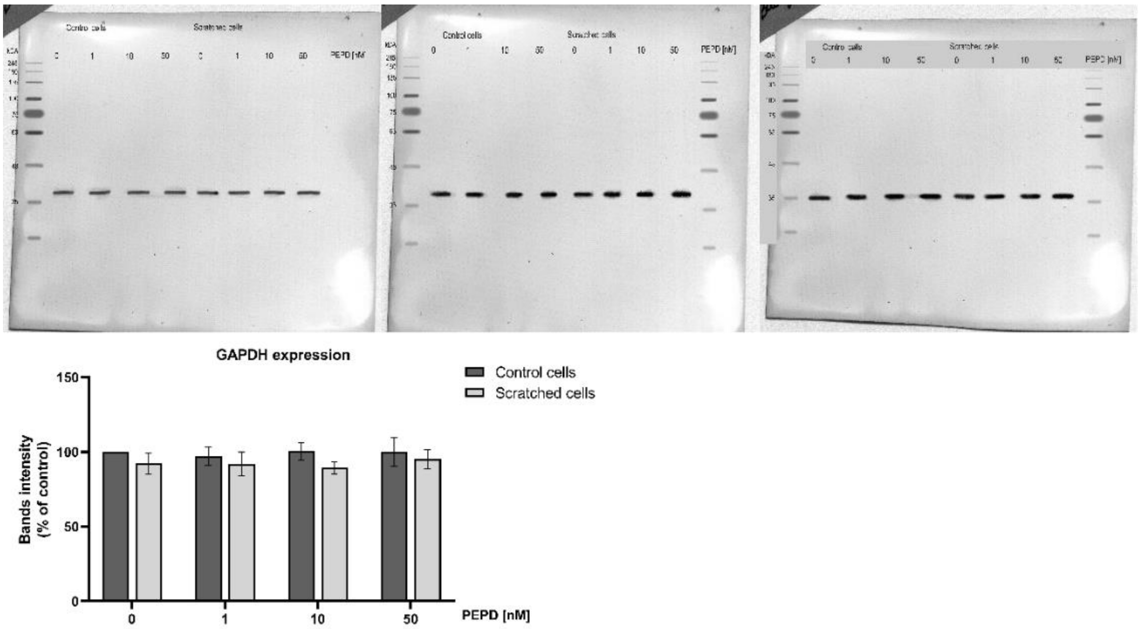

Figure S3. Representative blots from Western blotting analysis presented in Figure 4A.

EGFR expression and its intensity

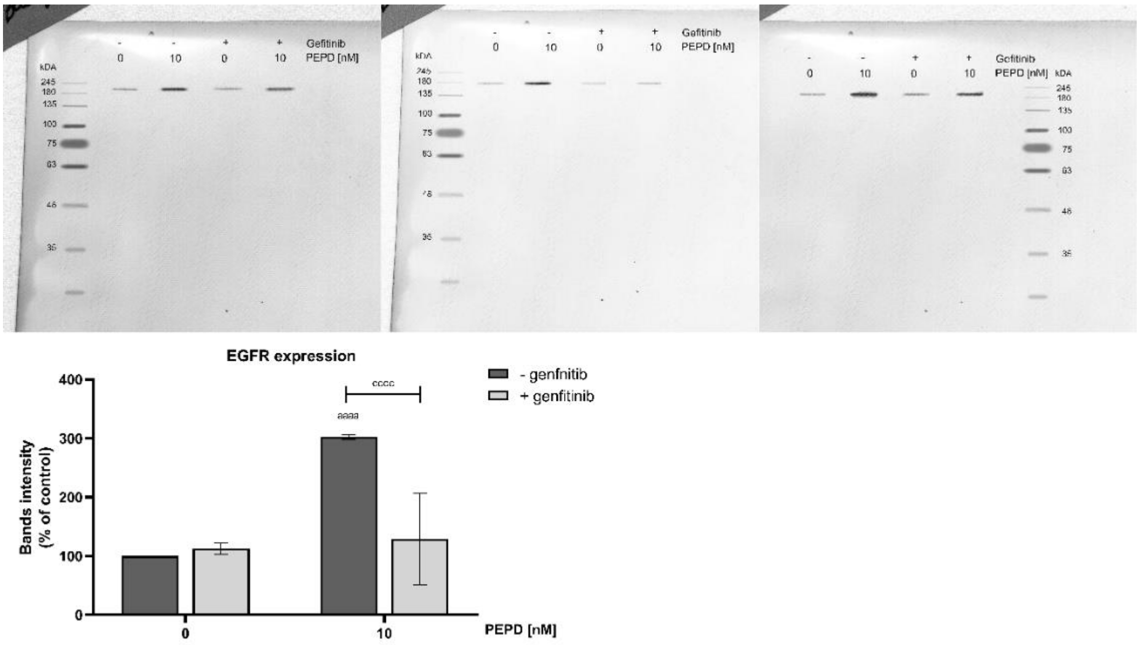

p-EGFR expression and its intensity

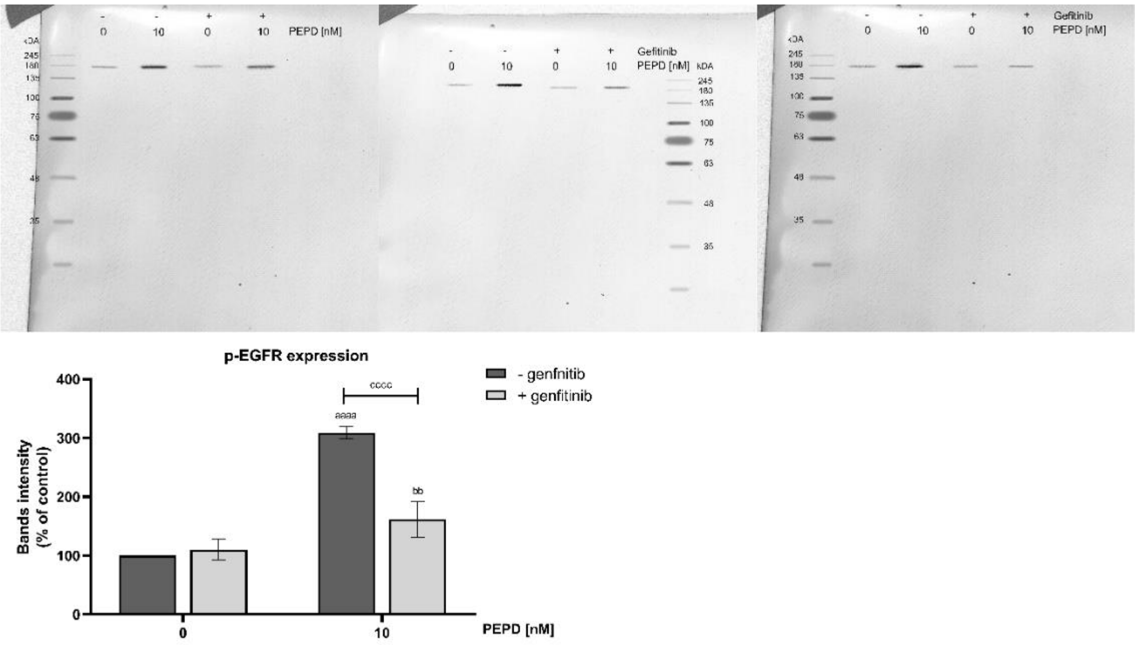

Akt expression and its intensity

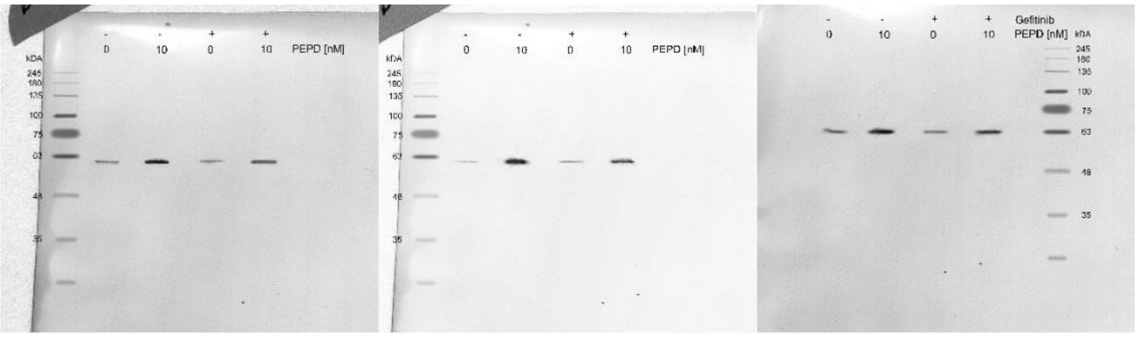

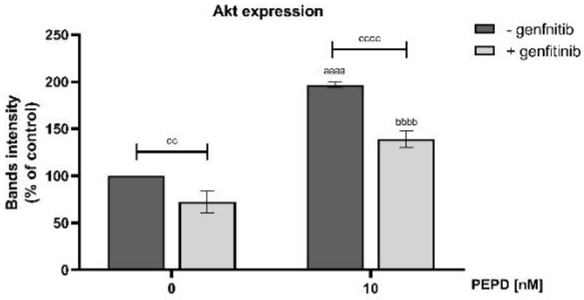

p-Akt expression and its intensity

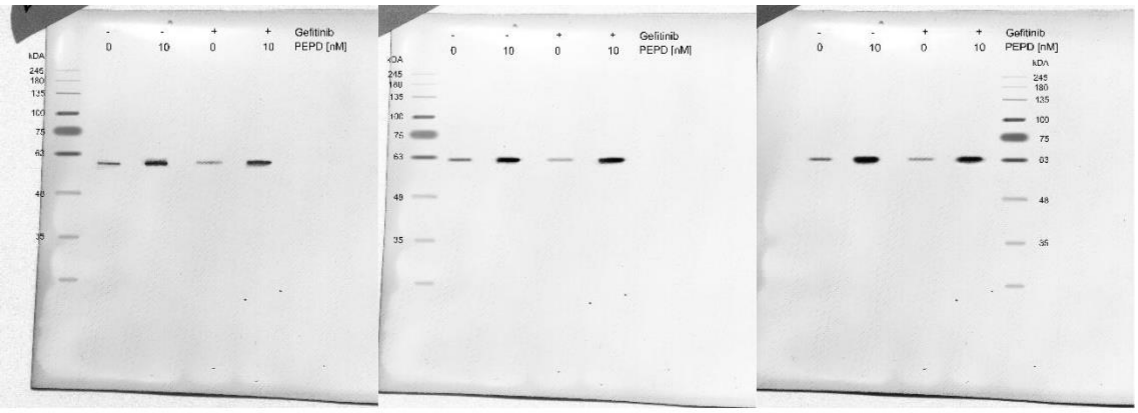

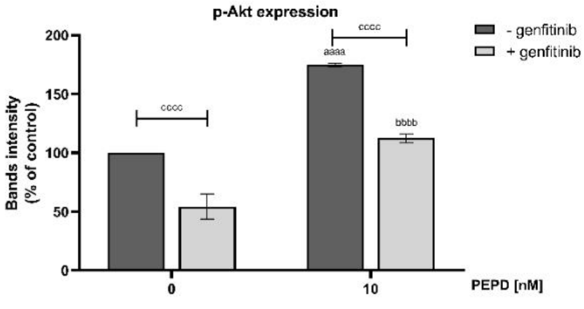

GAPDH expression and its intensity

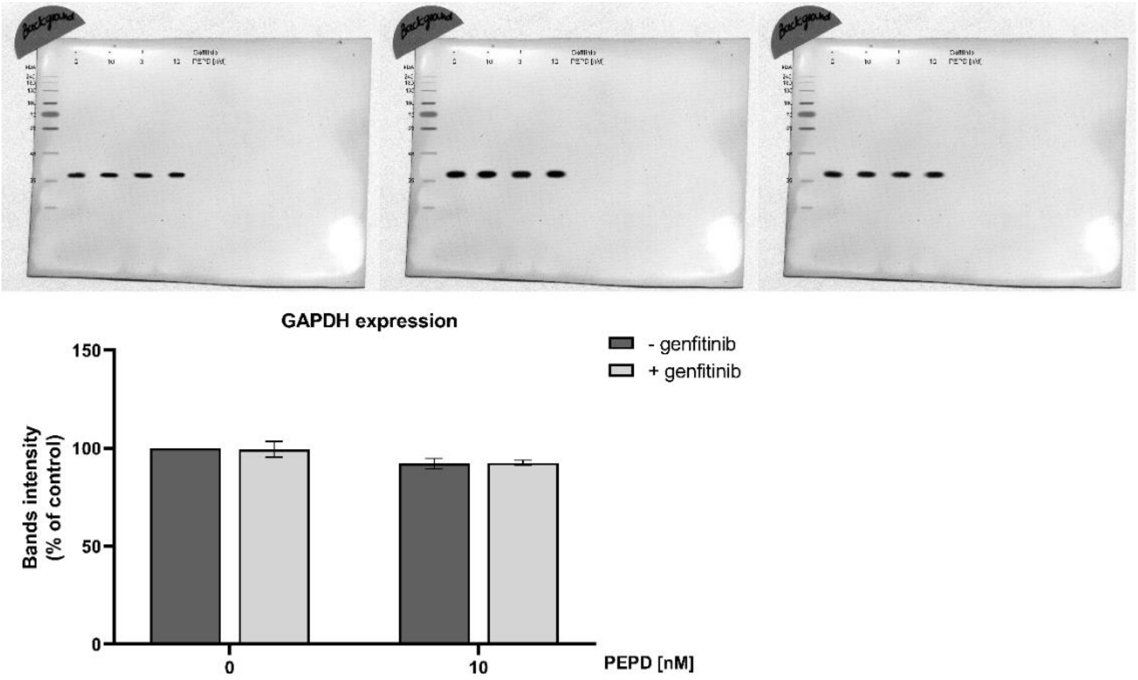

Figure S4. Representative blots from Western blotting analysis presented in Figure 5A.

Incubation time – 0 h

PEPD concentration – 0 nM

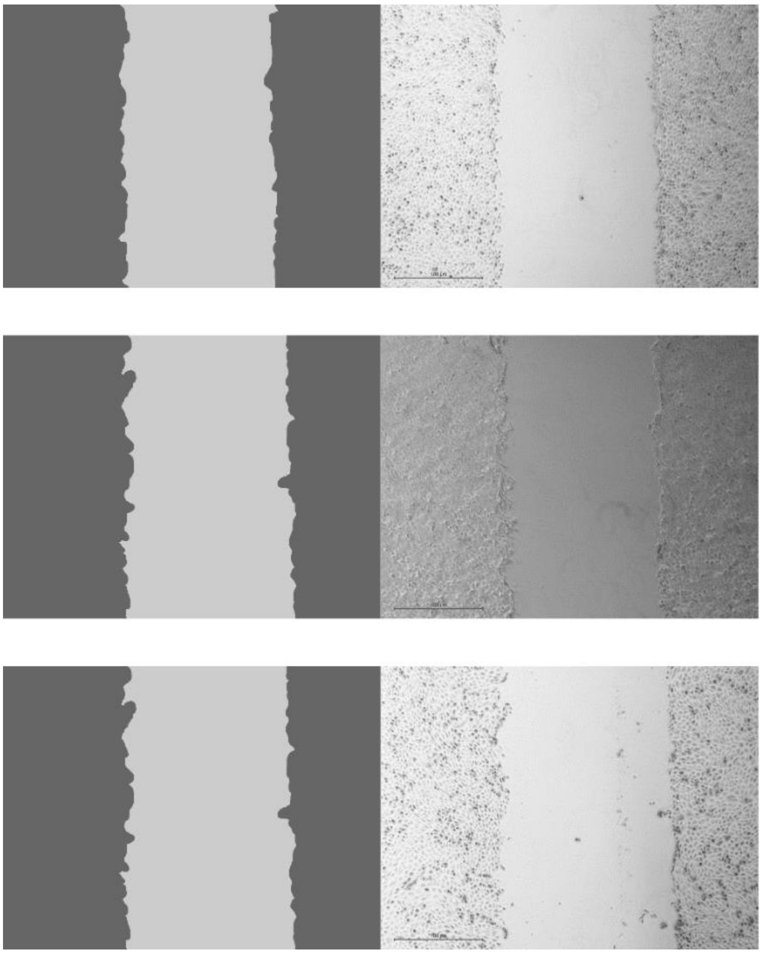

PEPD concentration – 10 nM

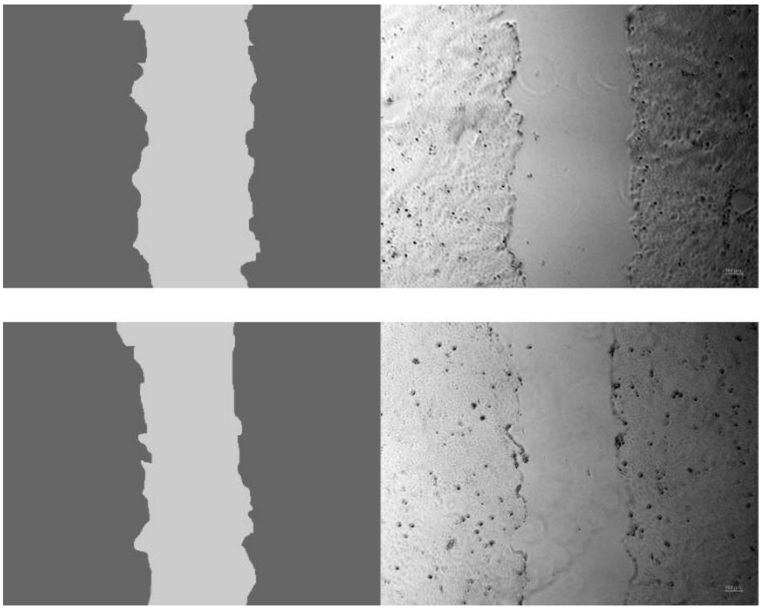

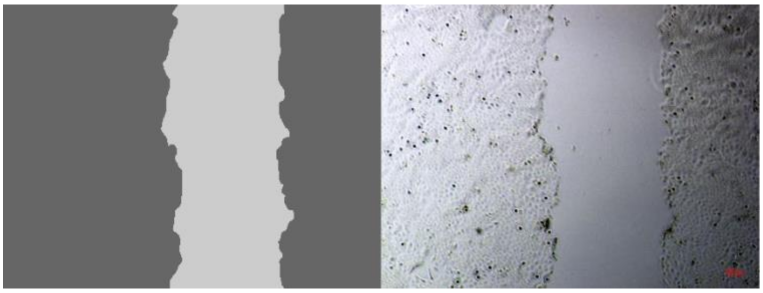

PEPD concentration – 0 nM + Gefinitib

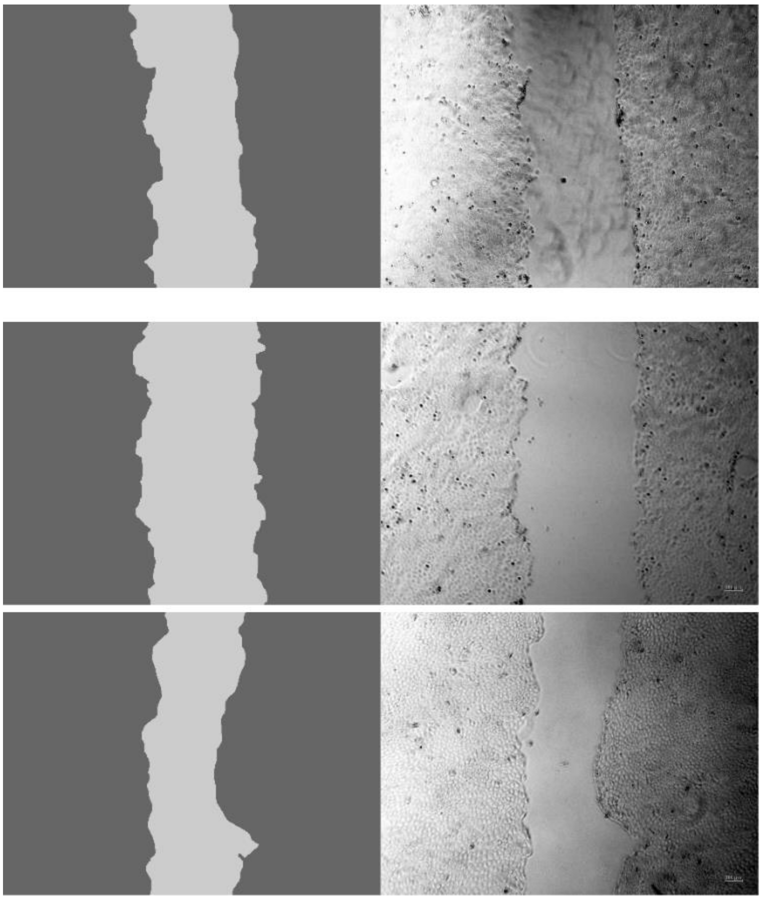

PEPD concentration – 10 nM + Gefinitib

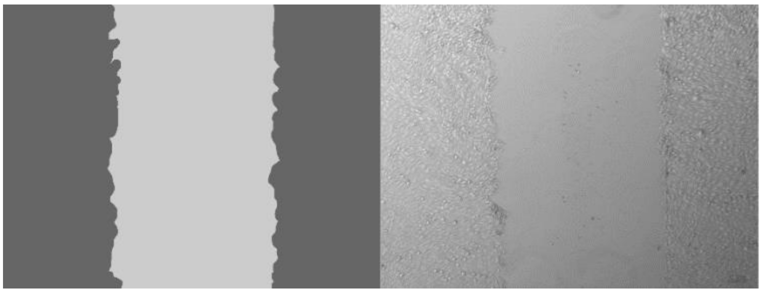

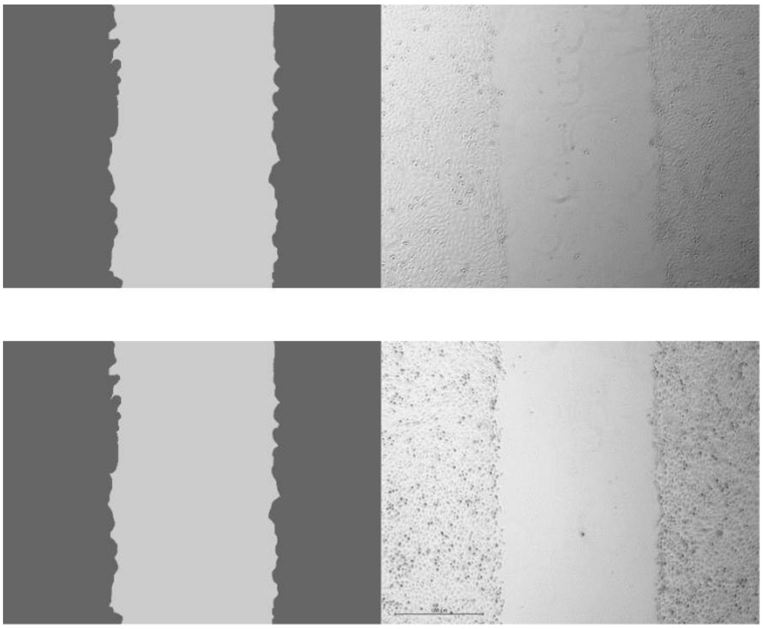

Incubation time – 24 h

PEPD concentration – 0 nM

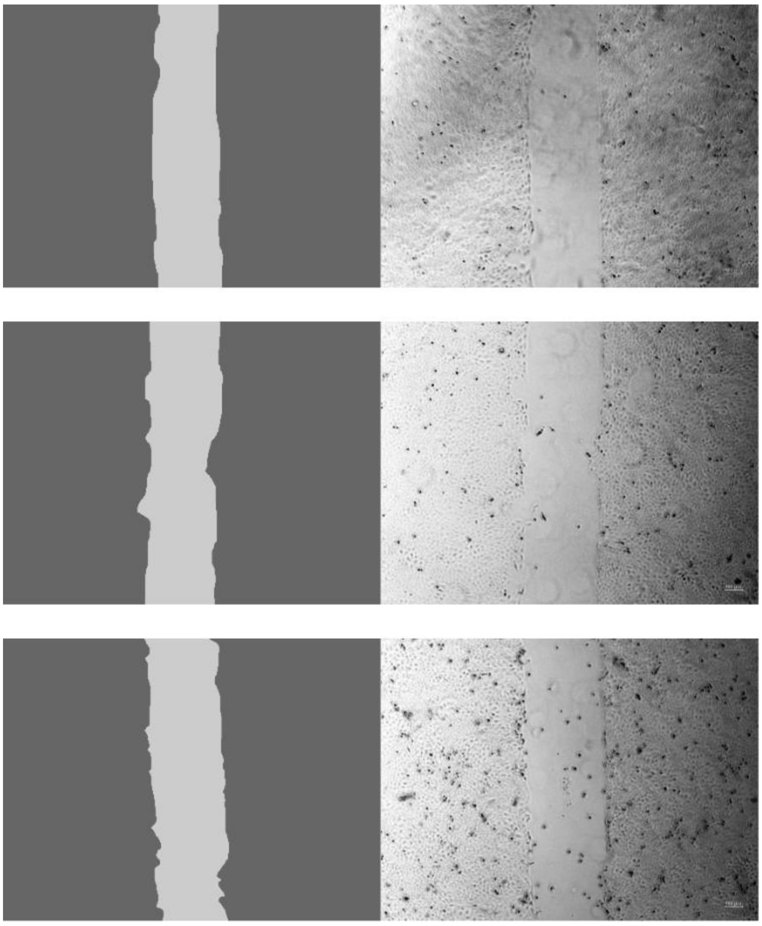

PEPD concentration – 10 nM

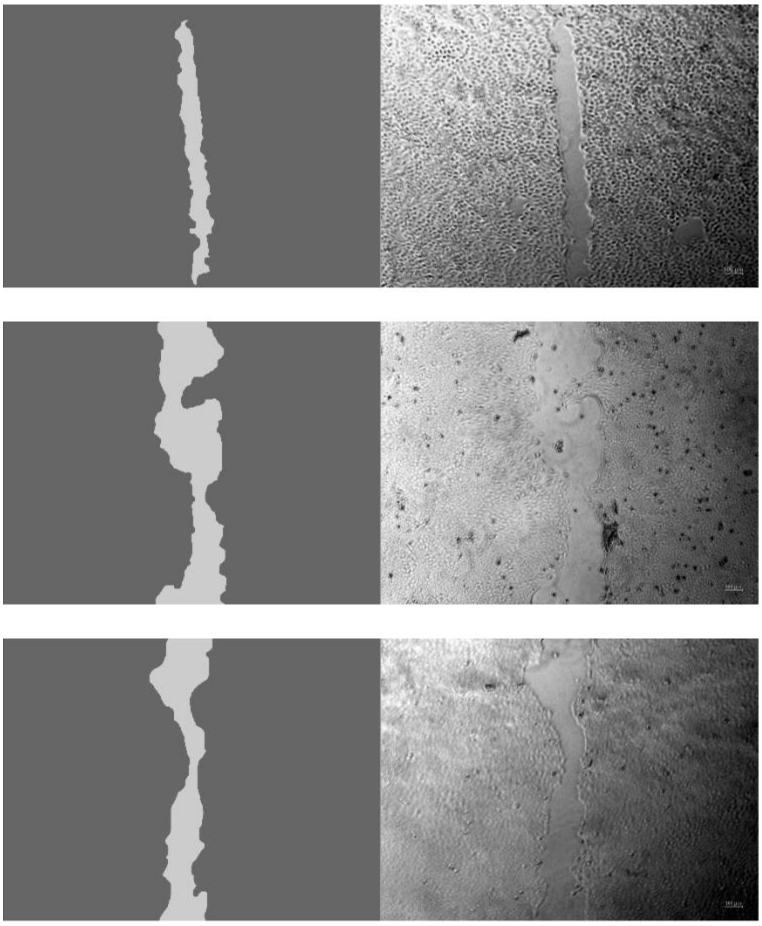

PEPD concentration – 0 nM + Gefitinib

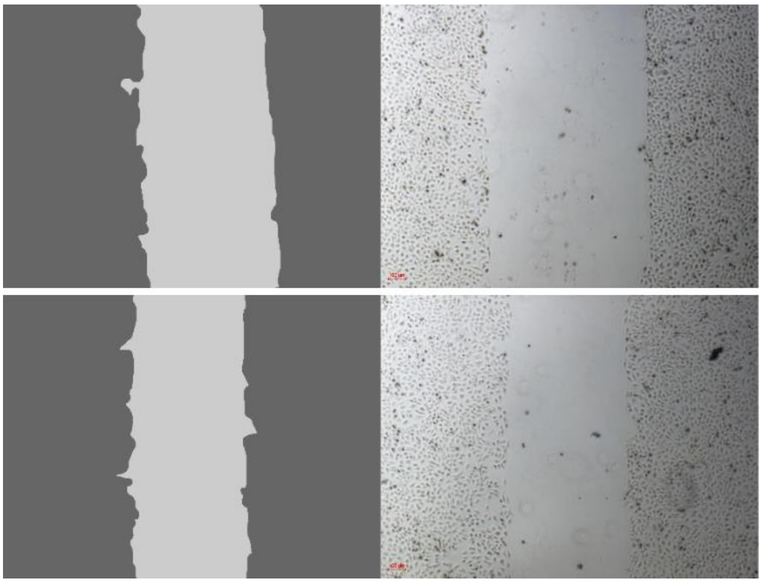

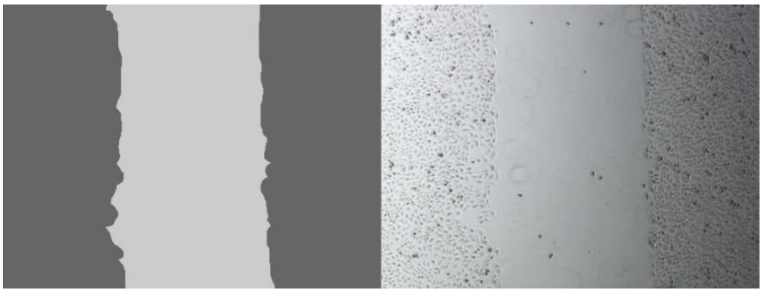

PEPD concentration – 10 nM + Gefinitib

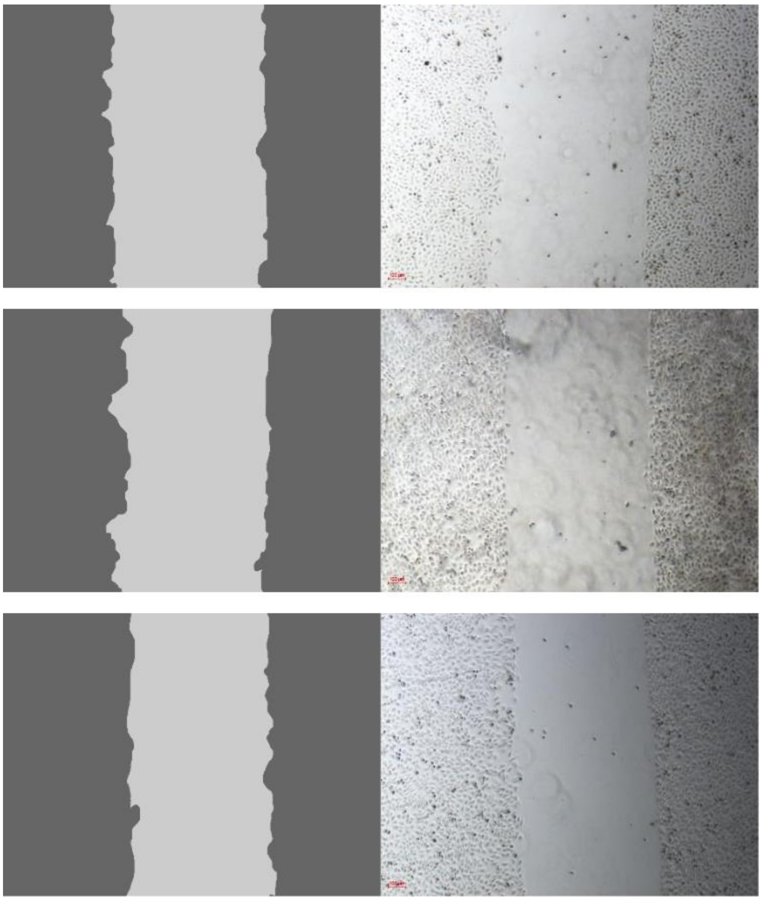

Figure S5. Representative images of HaCaT cell migration presented on Figure 5B.

EGFR expression and its intensity

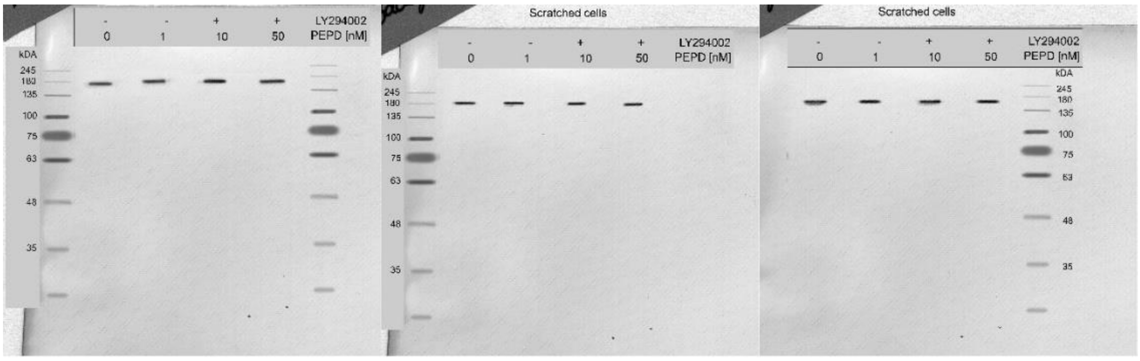

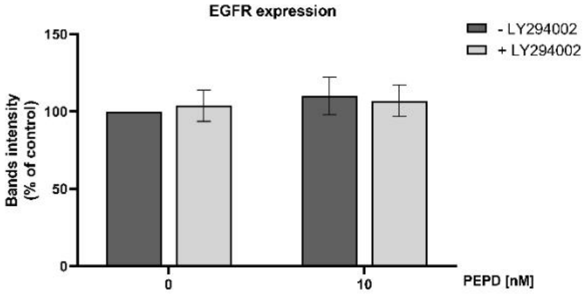

p-EGFR expression and its intensity

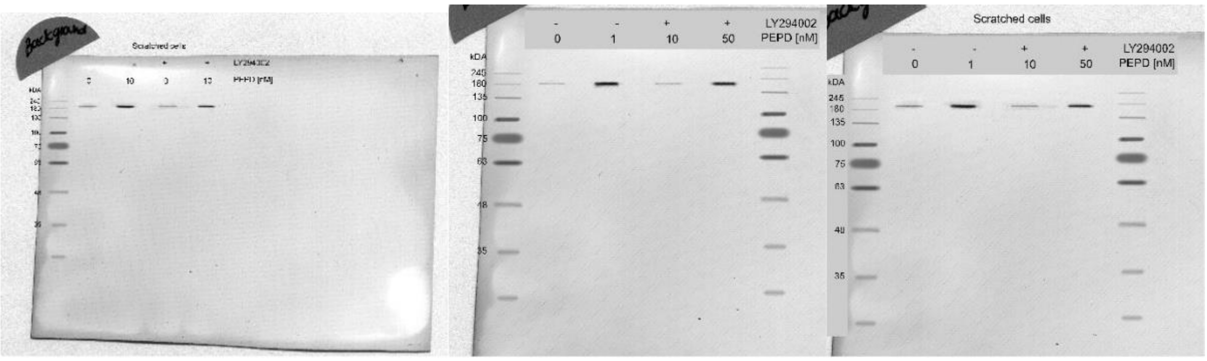

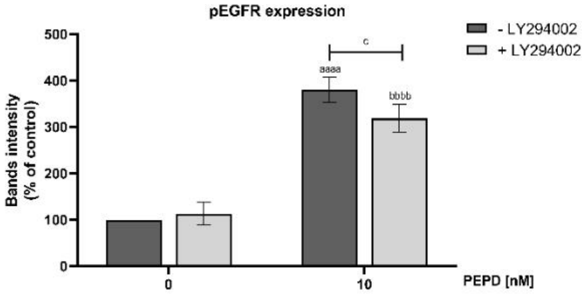

PI3K expression and its intensity

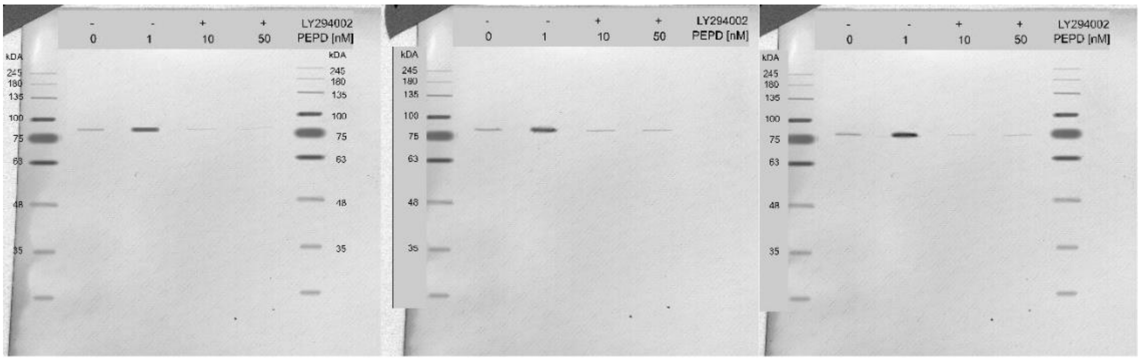

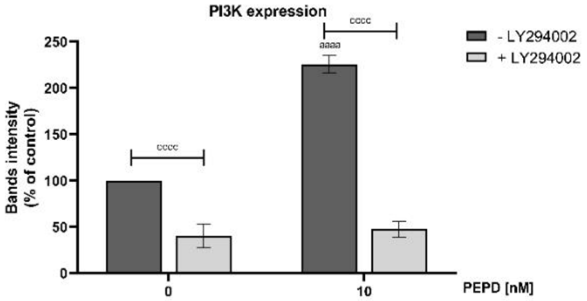

p-PI3K expression and its intensity

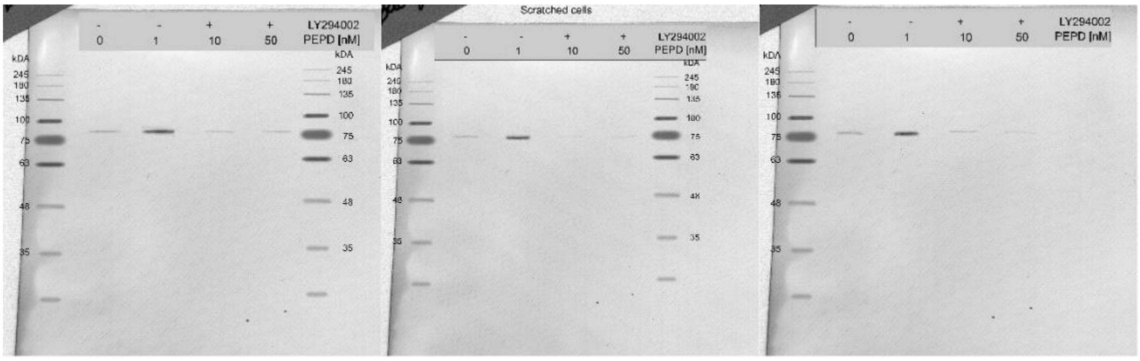

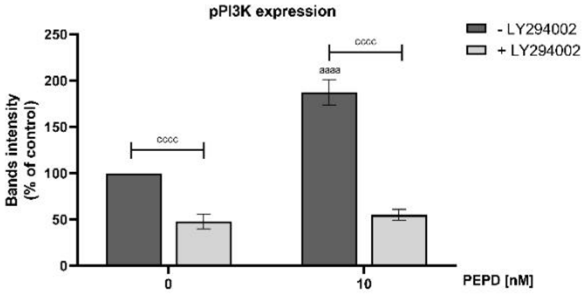

Akt expression and its intensity

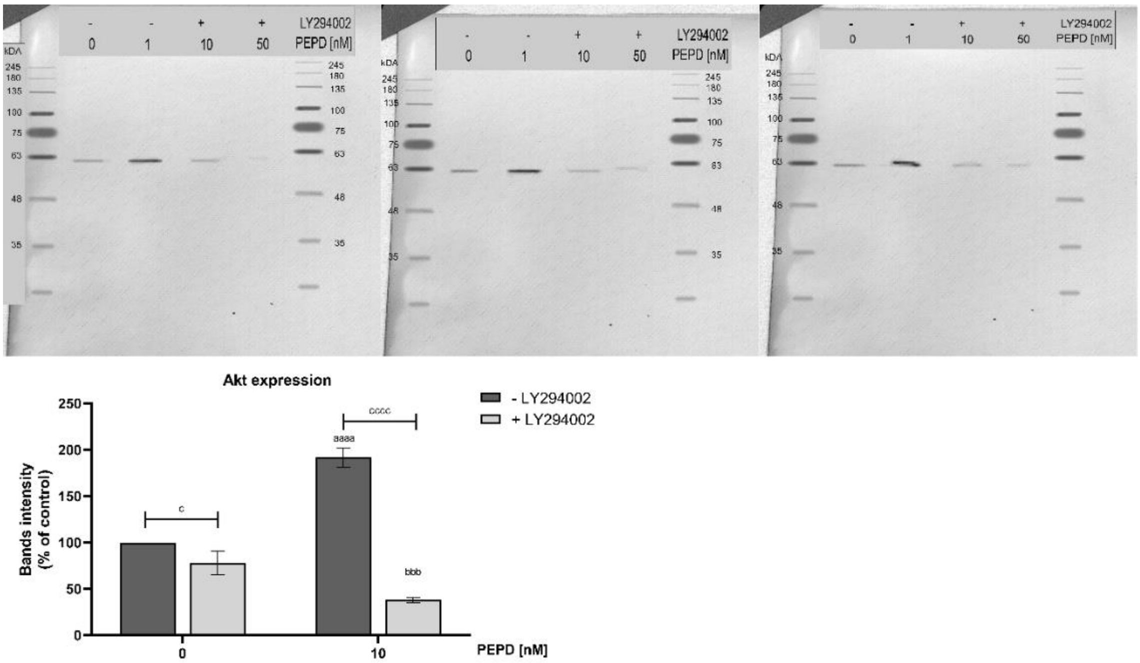

p-Akt expression and its intensity

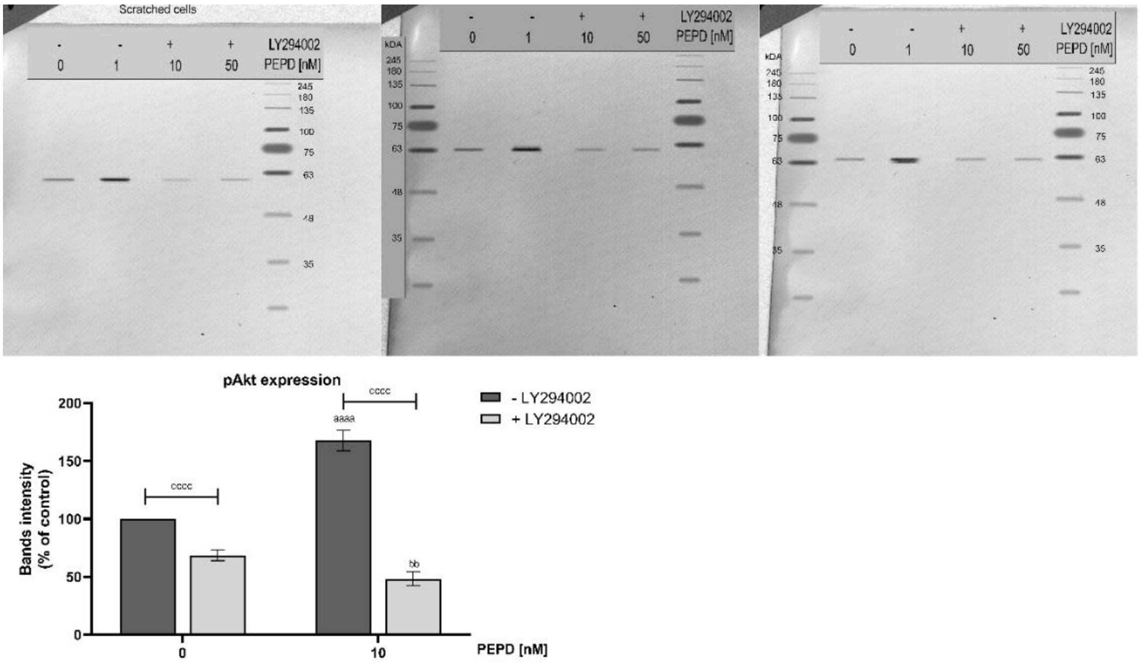

mTOR expression and its intensity

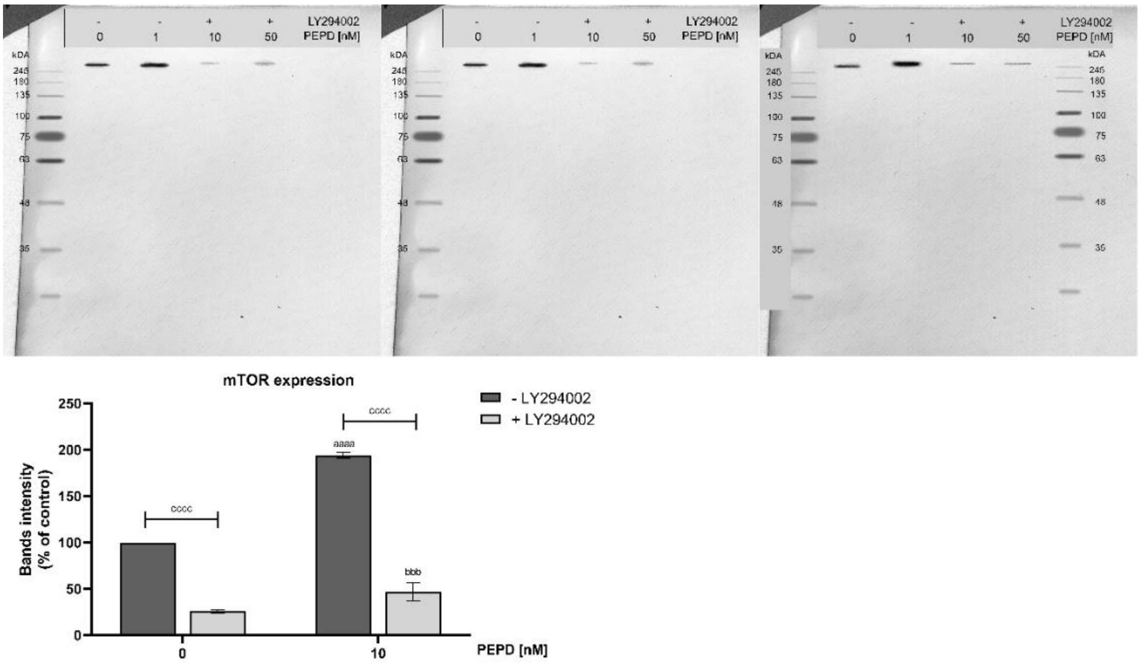

p-mTOR expression and its intensity

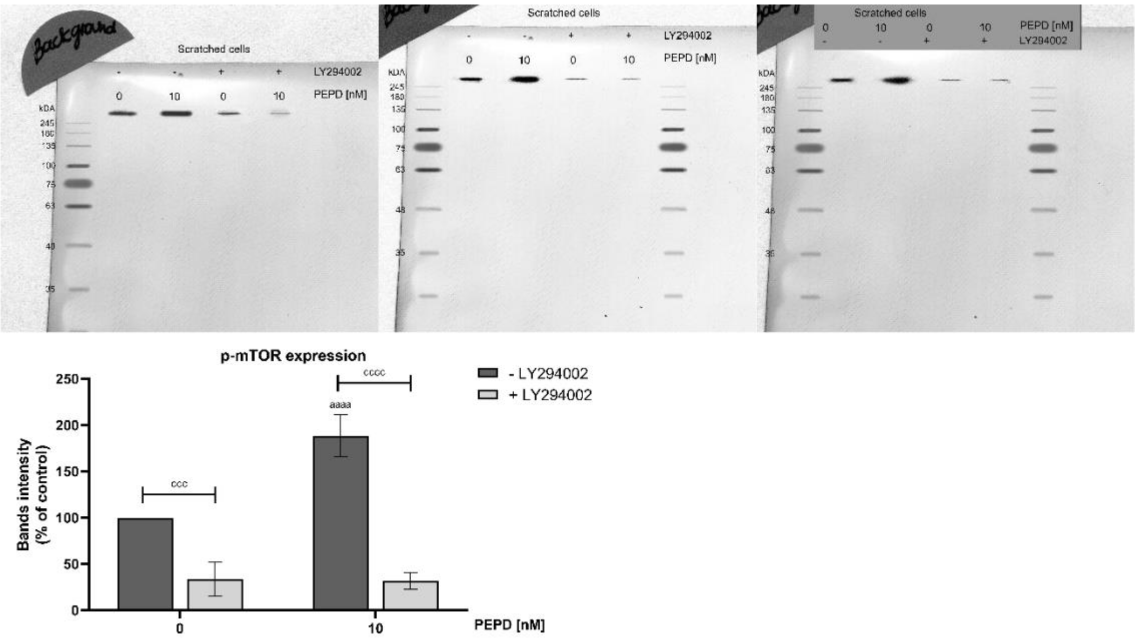

GAPDH expression and its intensity

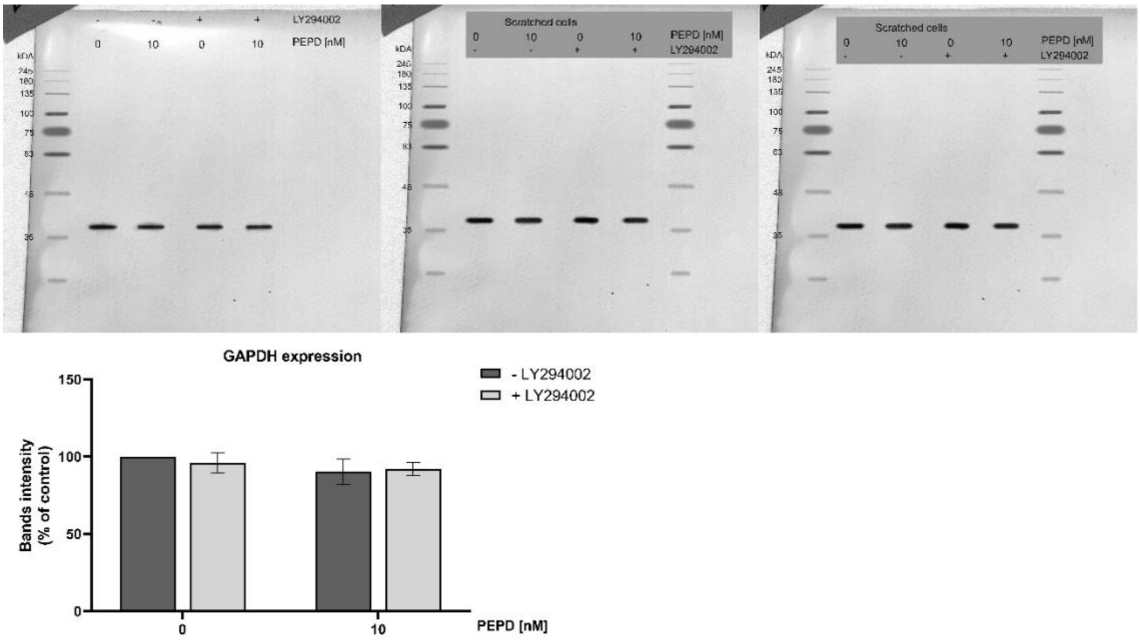

Figure S6. Representative blots from Western blotting analysis presented in Figure 6A.

Incubation time – 0 h

PEPD concentration – 0 nM

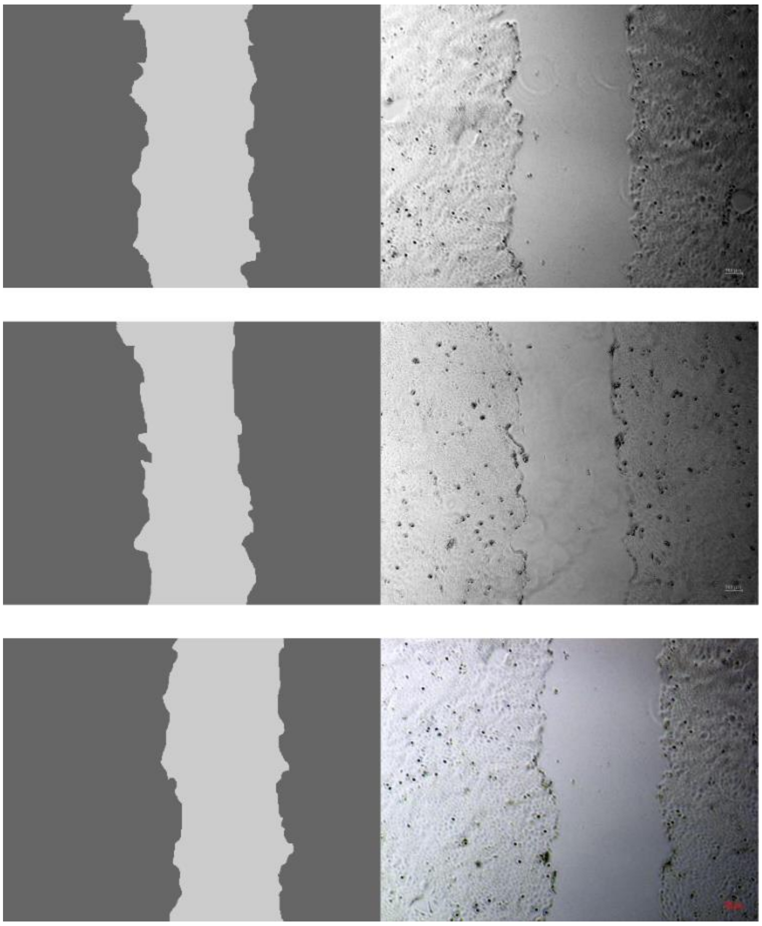

PEPD concentration – 10 nM

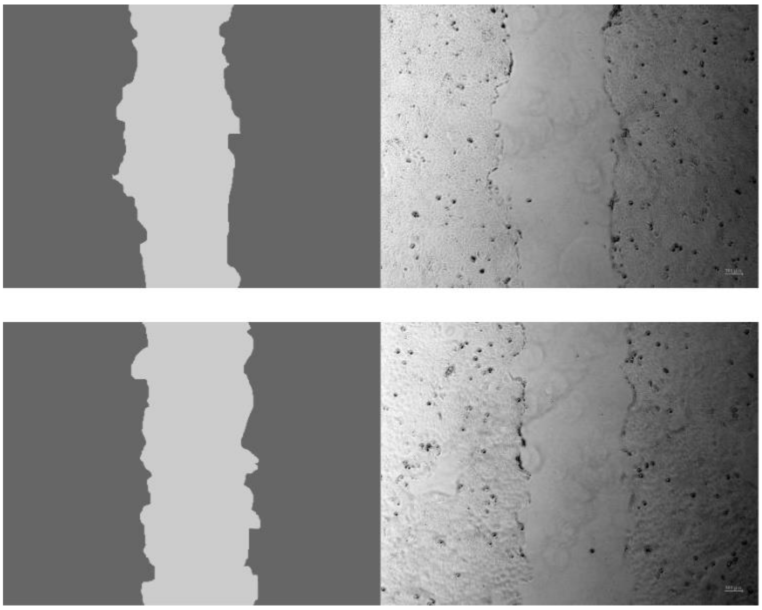

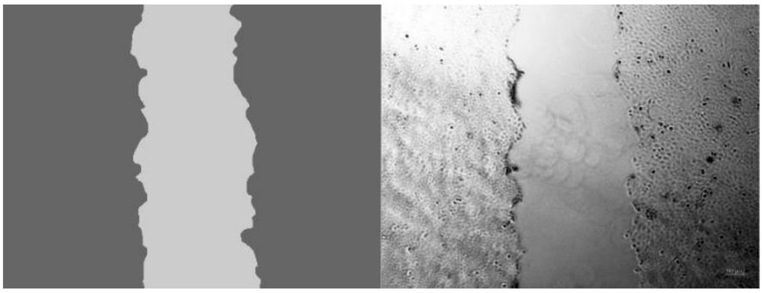

PEPD concentration – 0 nM + LY294002

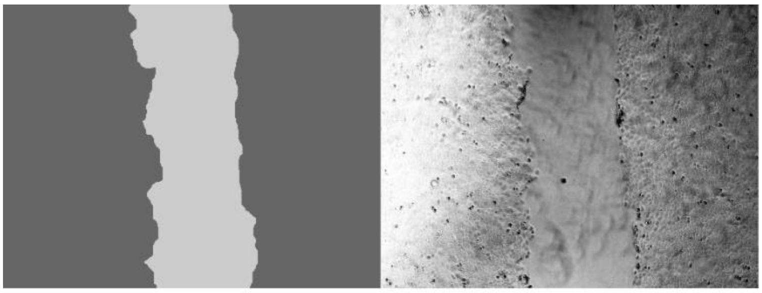

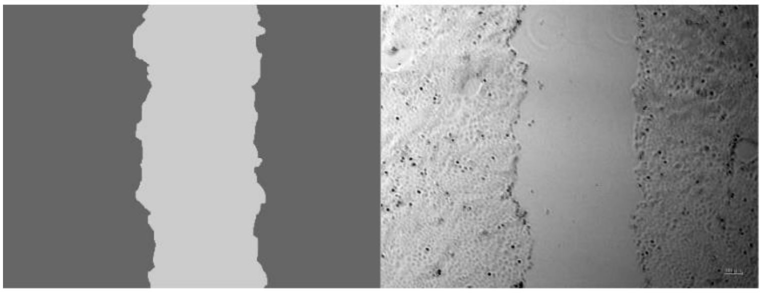

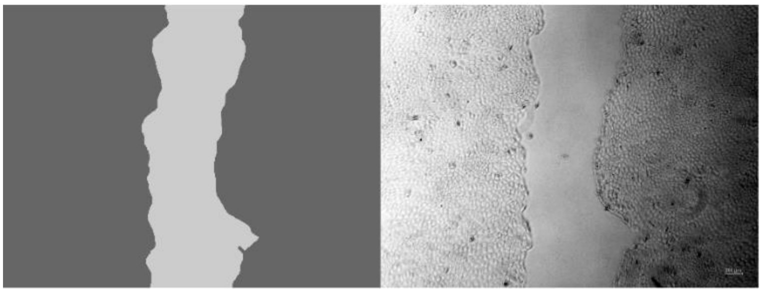

PEPD concentration – 10 nM + LY294002

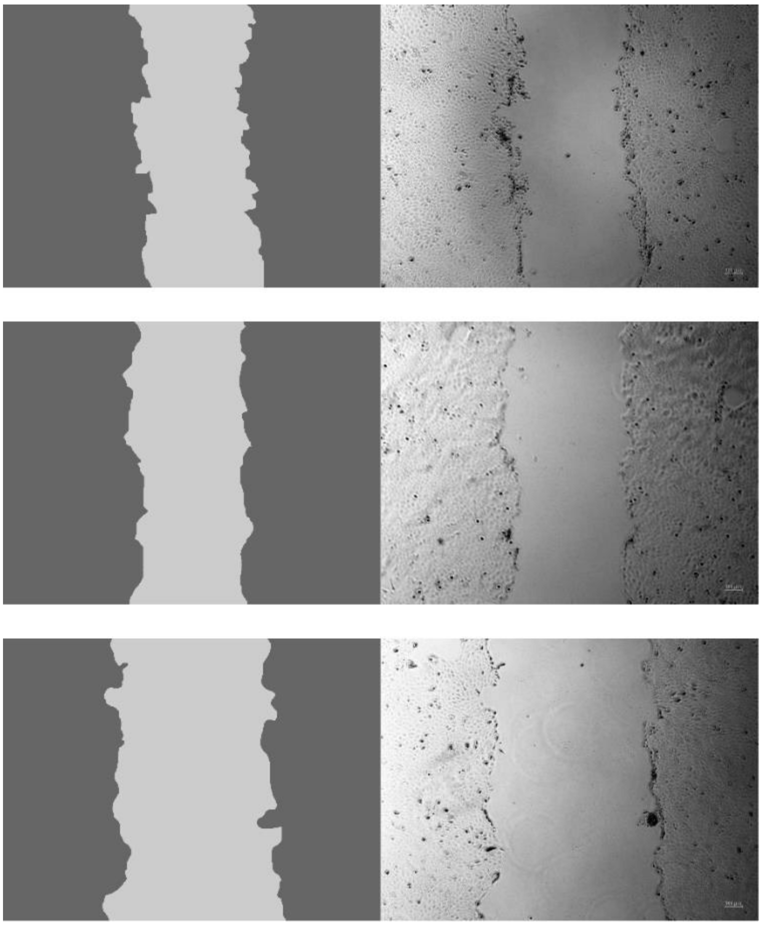

Incubation time – 24 h

PEPD concentration – 0 nM

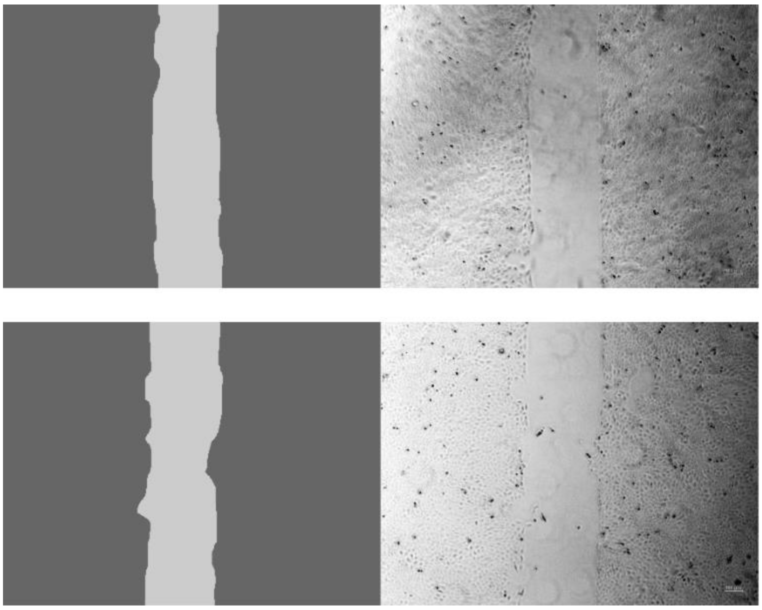

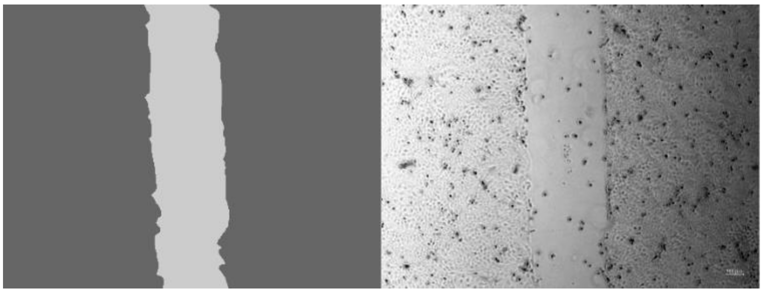

PEPD concentration – 10 nM

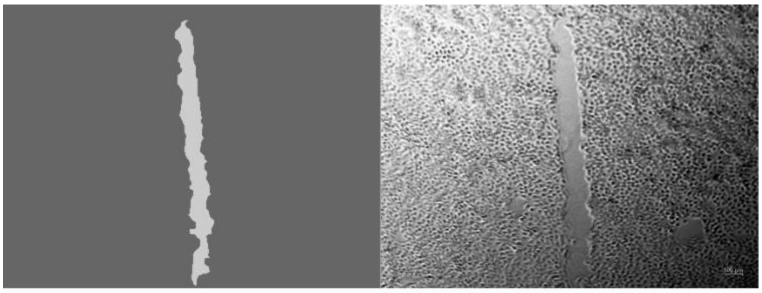

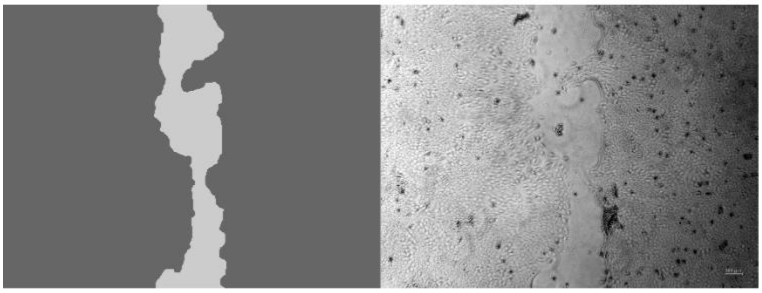

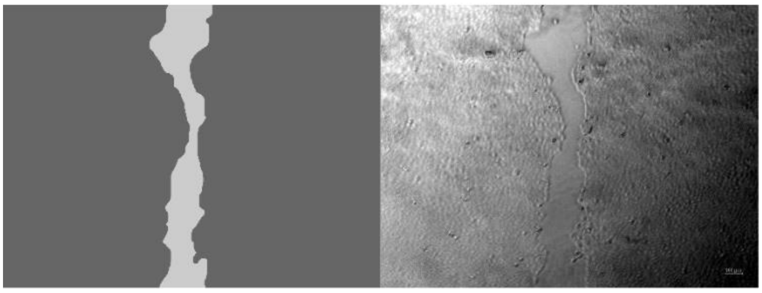

1.1.4.1.1. PEPD concentration – 0 nM + LY294002

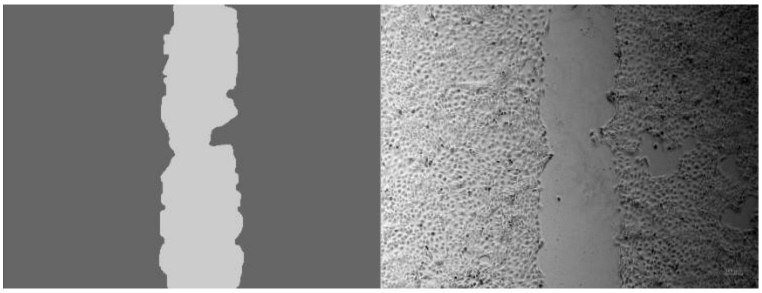

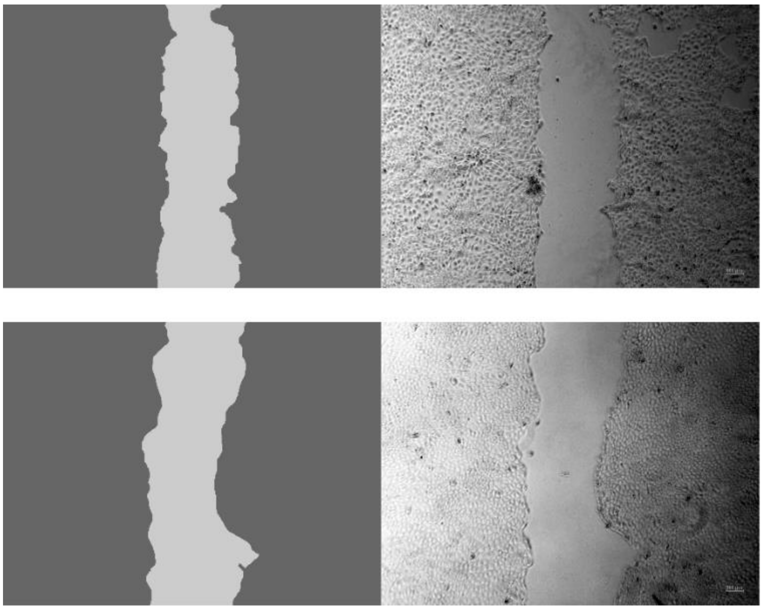

PEPD concentration – 10 nM + LY294002

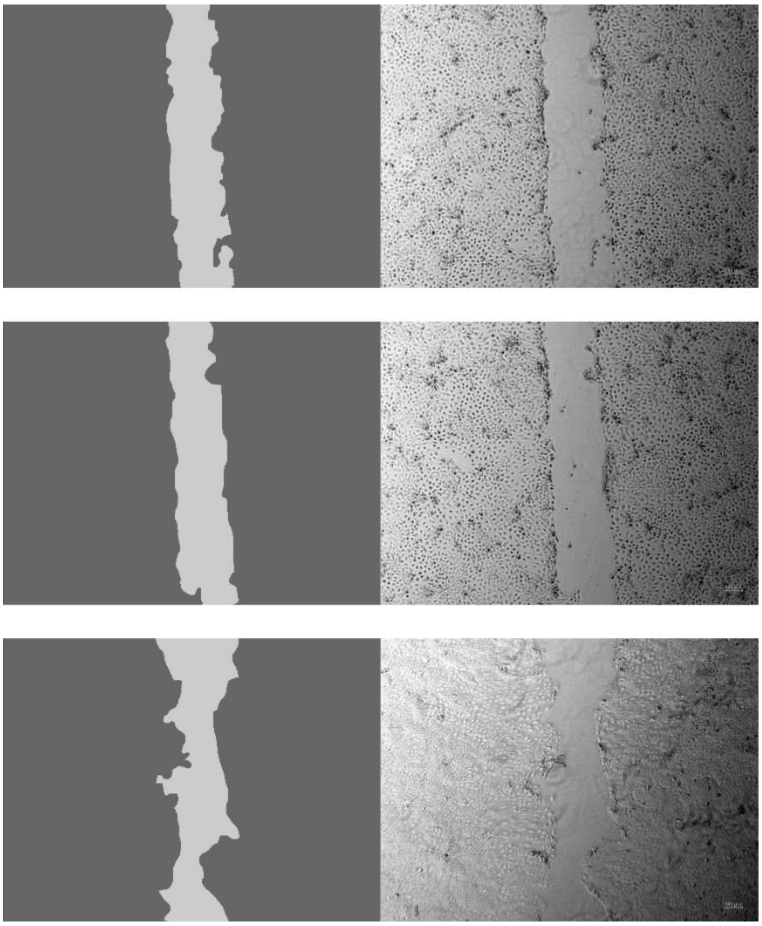

Figure S7. Representative images of HaCaT cell migration presented in Figure 6C.

IGF-1R expression and its intensity

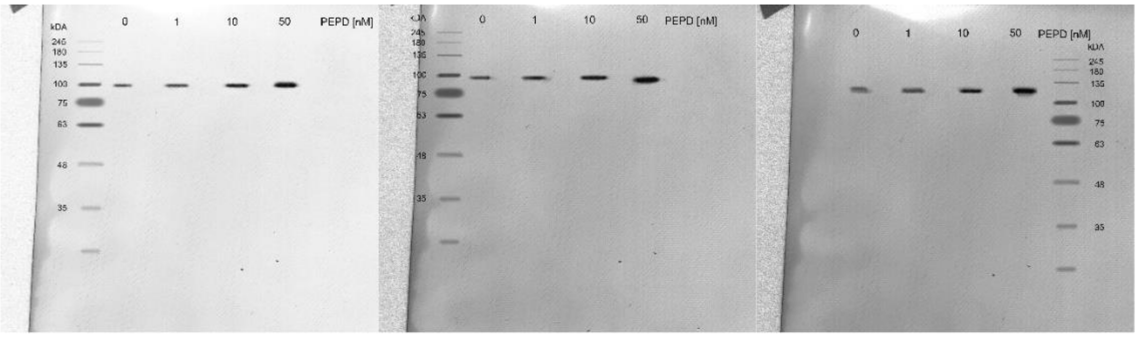

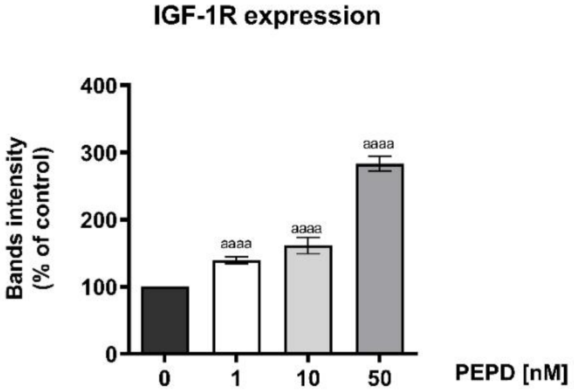

B$_1$-integrin receptor expression and its intensity

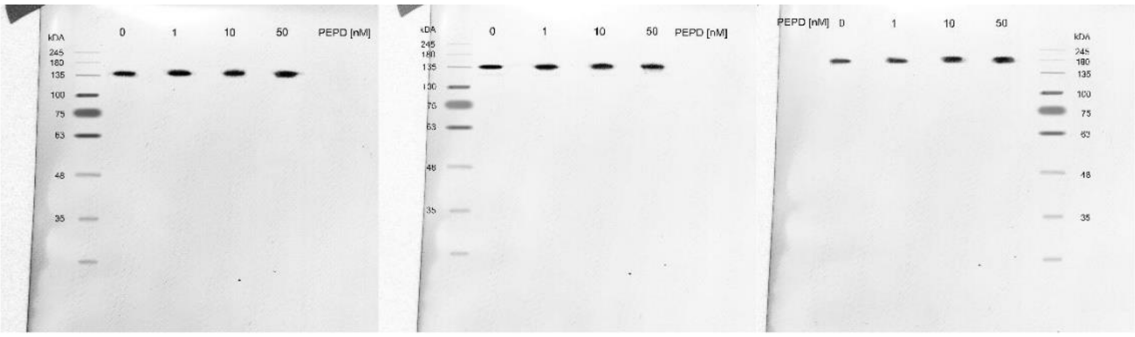

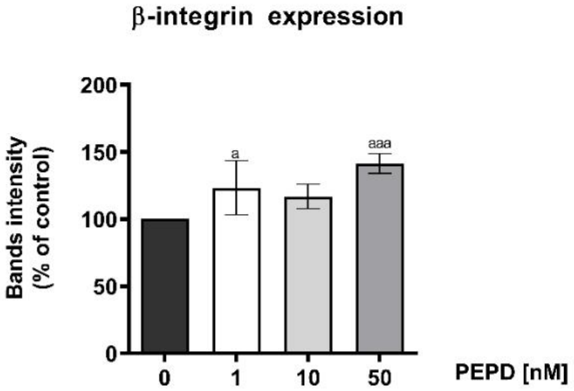

FAK expression and its intensity

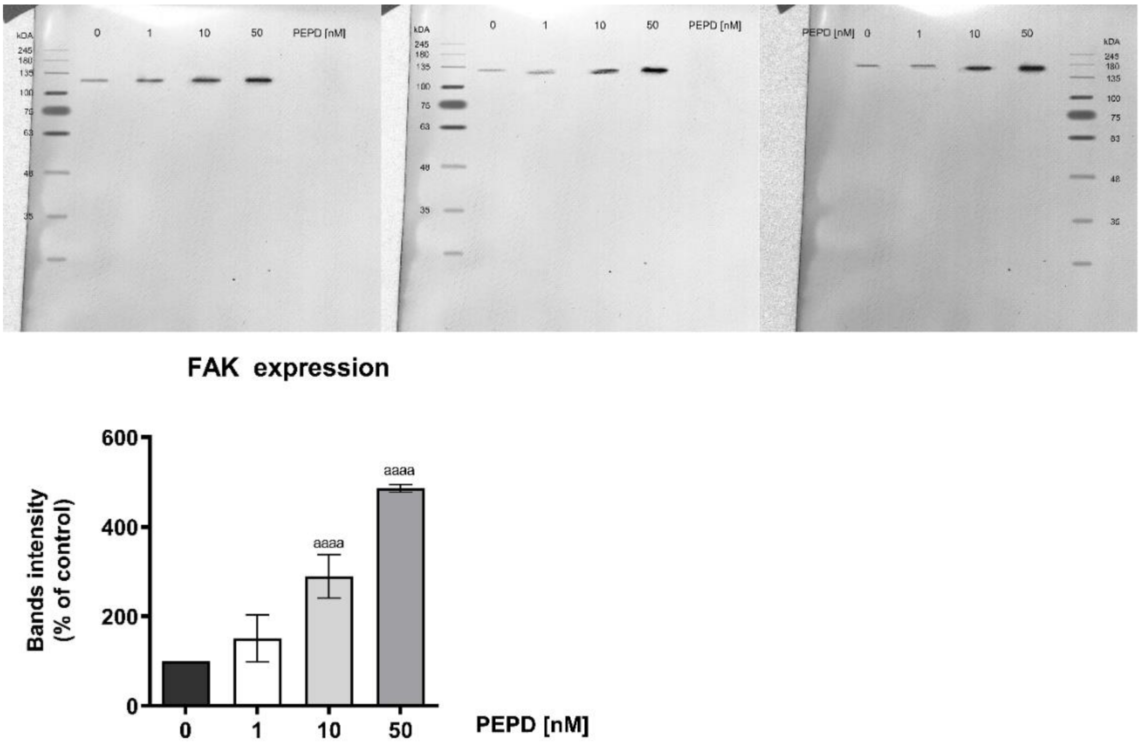

p-FAK expression and its intensity

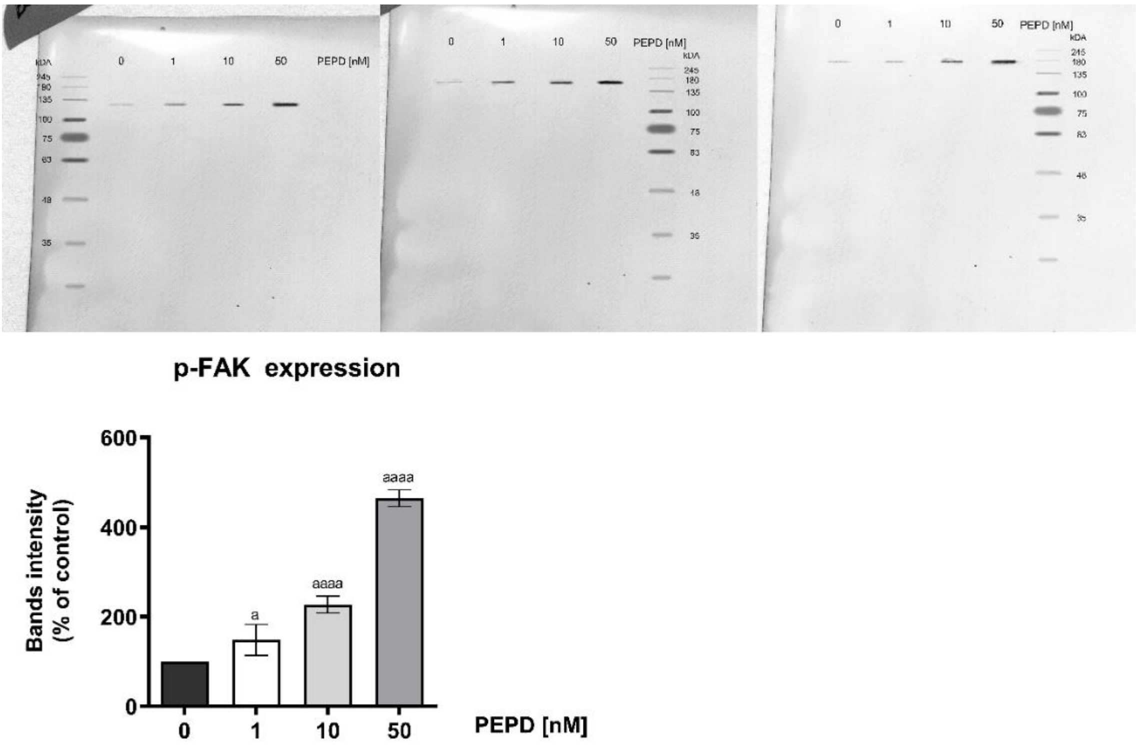

Grb2 expression and its intensity

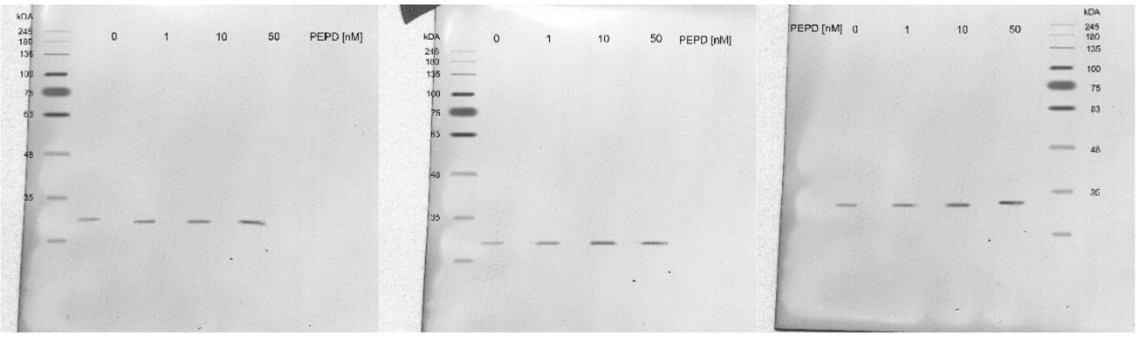

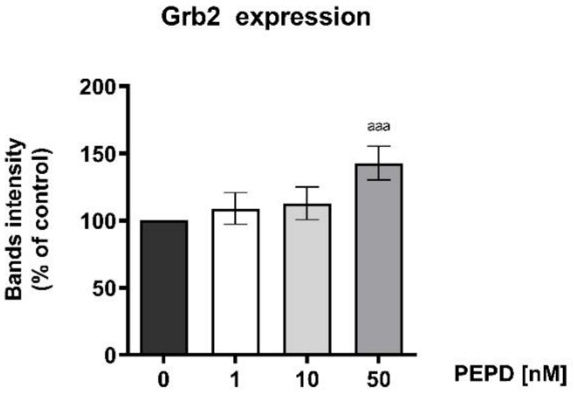

Sos1 expression and its intensity

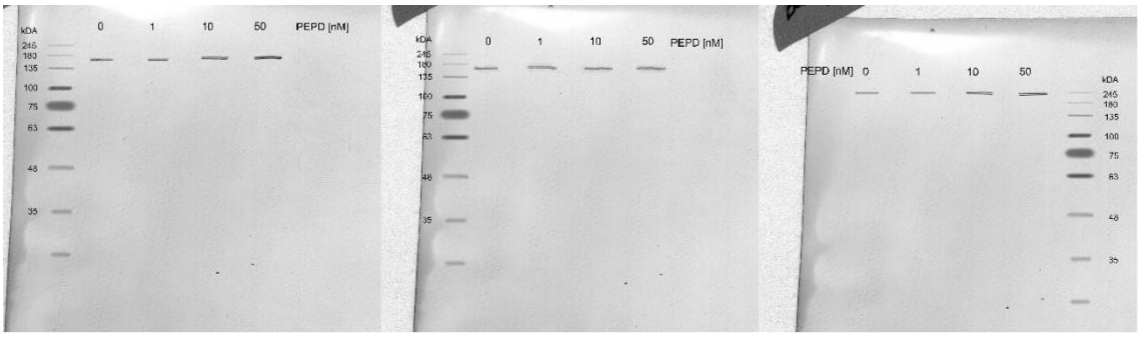

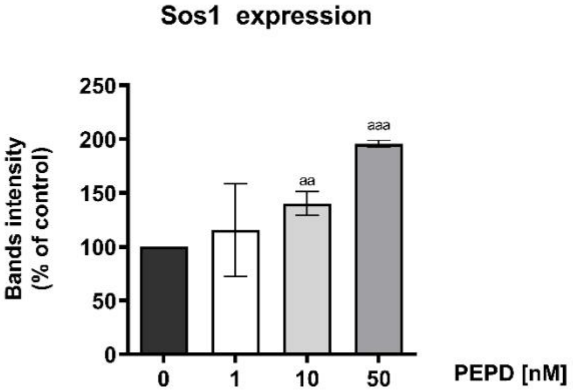

GAPDH expression and its intensity

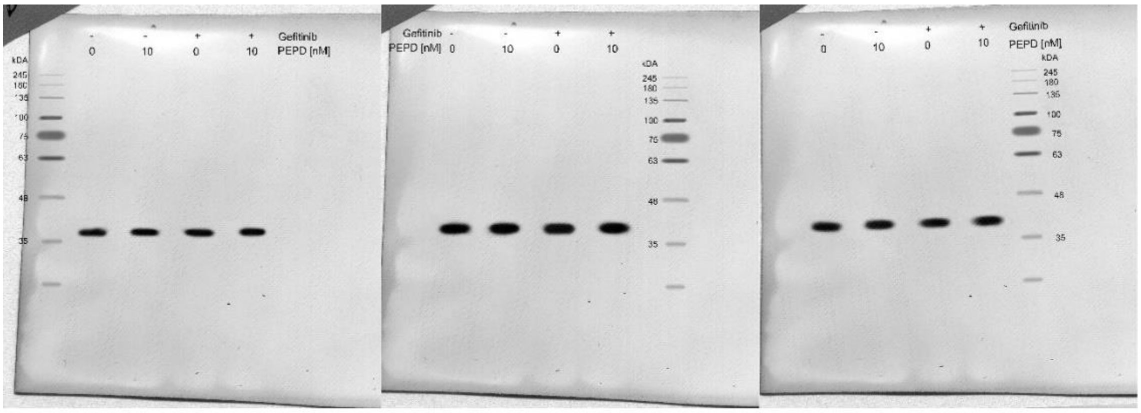

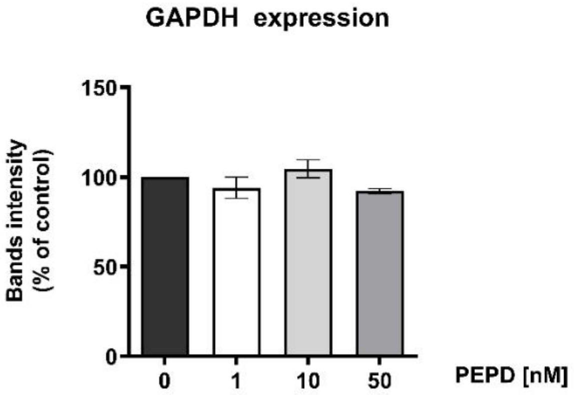

Figure S8. Representative blots from Western blotting analysis presented in Figure 7A.

E-cadherin expression and its intensity

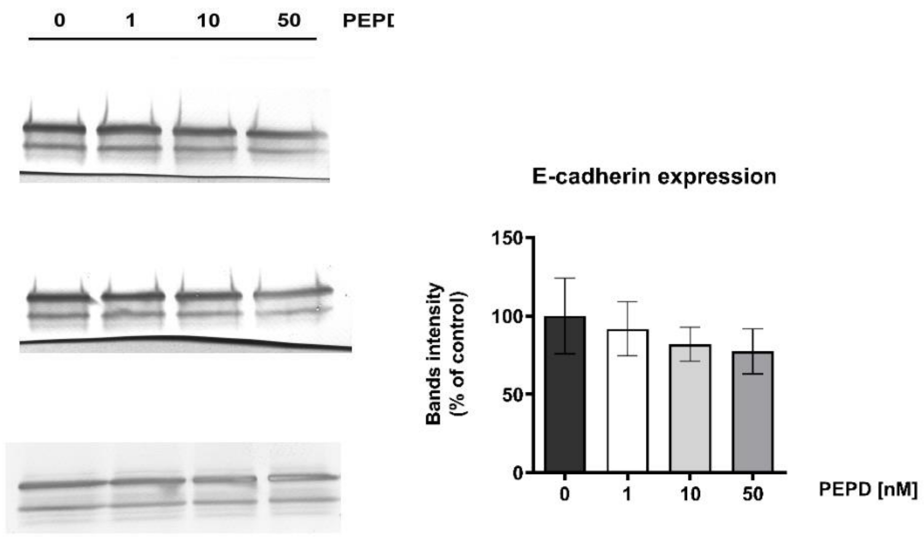

N-cadherin expression and its intensity

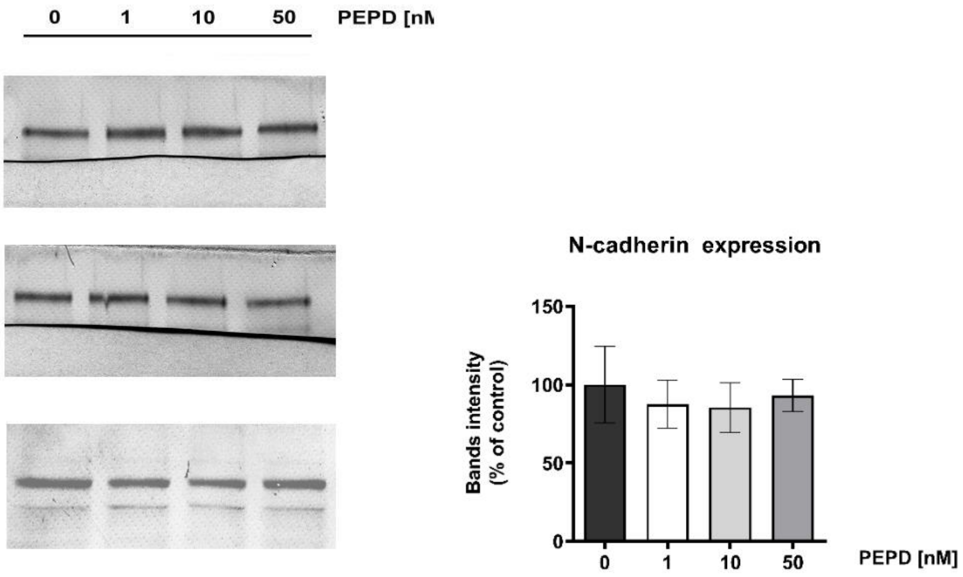

TGF β receptor expression and its intensity

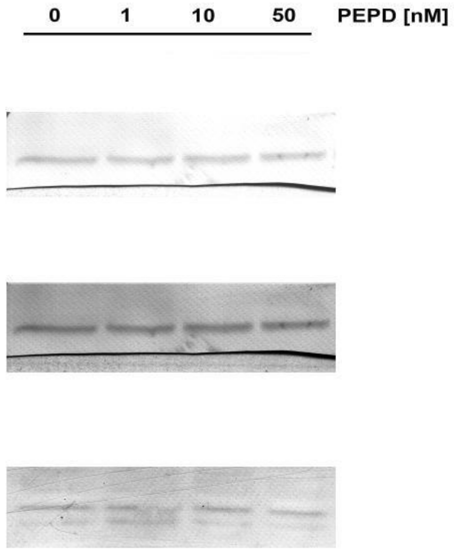

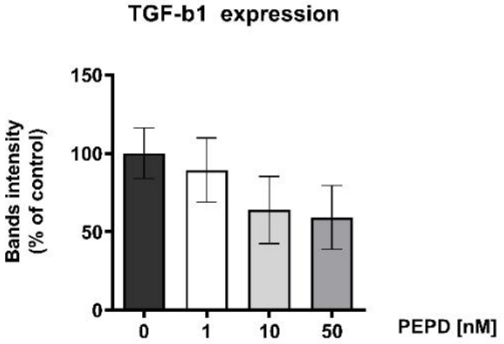

ERK 1/2 expression and its intensity

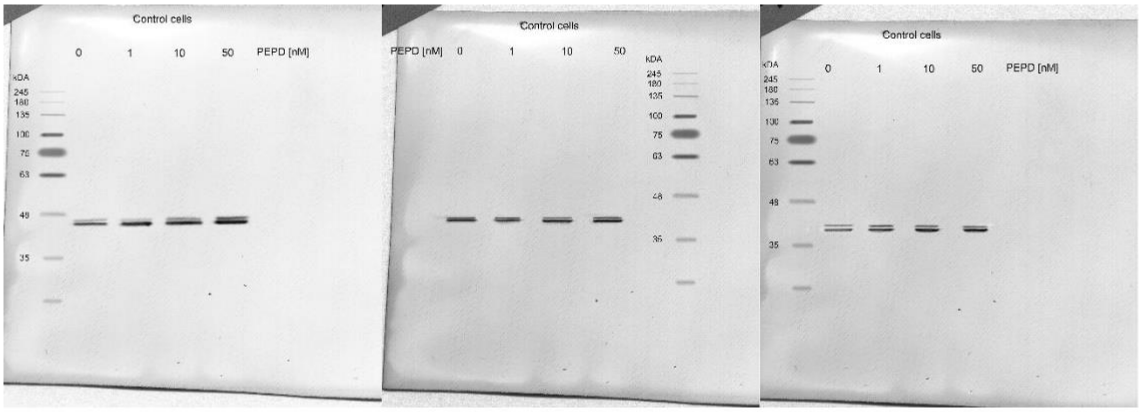

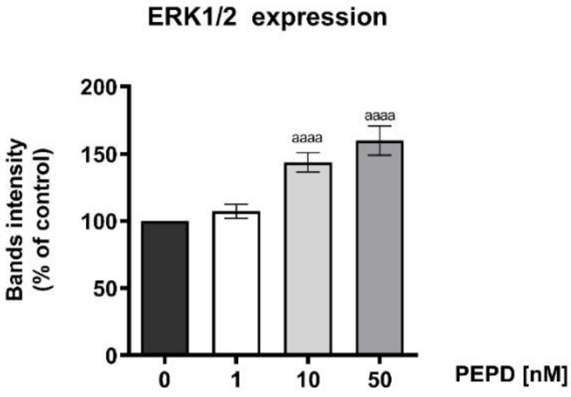

GAPDH expression and its intensity

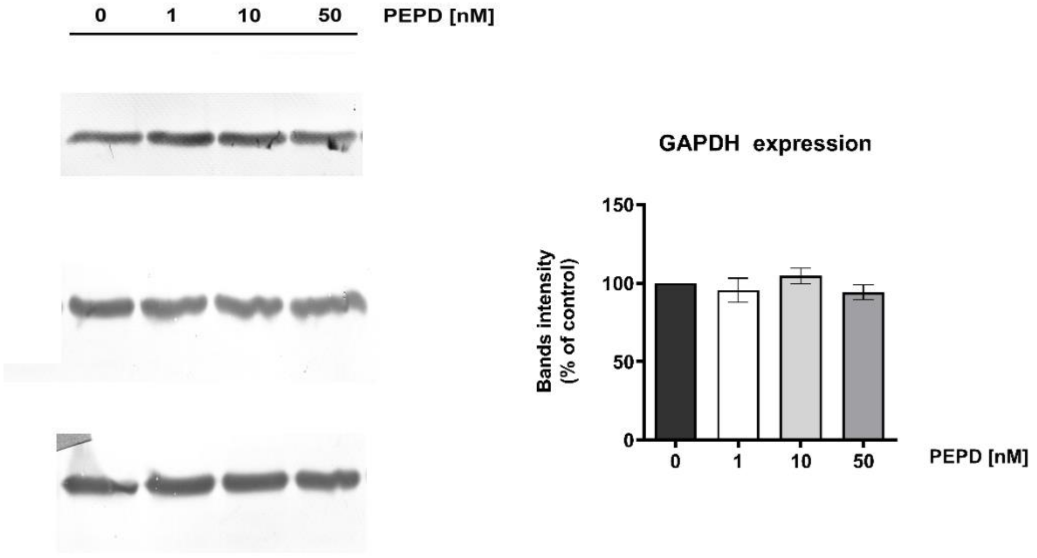

Figure S9. Representative blots from Western blotting analysis presented in Figure 8A.

NF-kb expression and its intensity

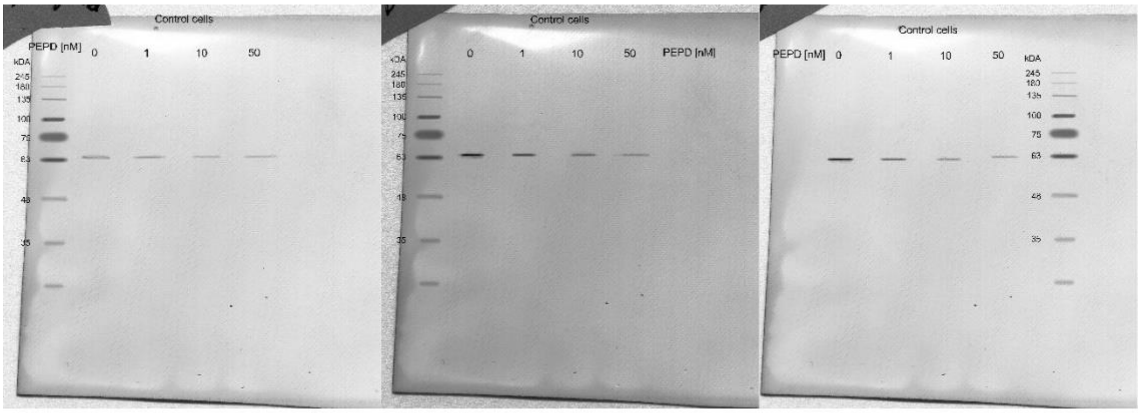

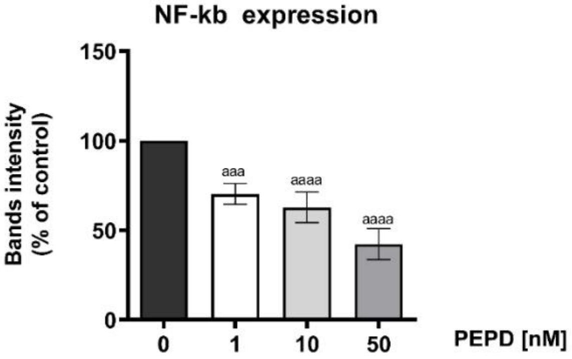

GAPDH expression and its intensity

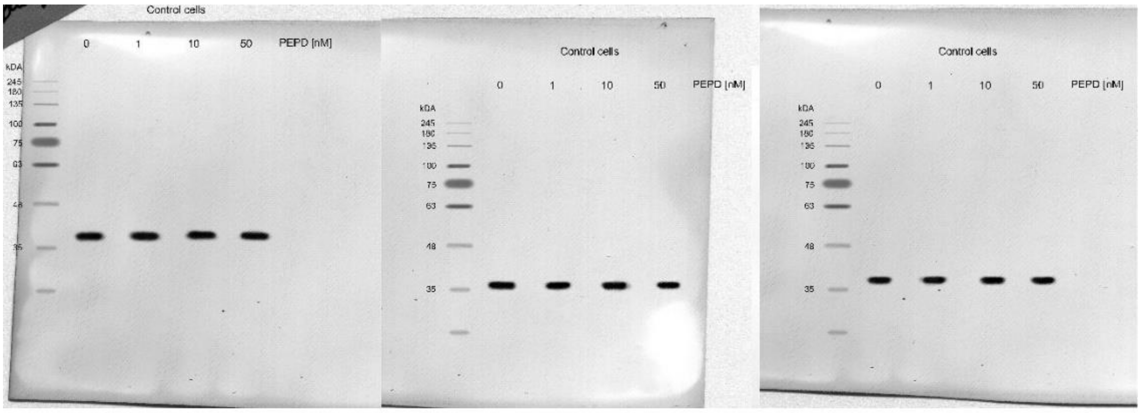

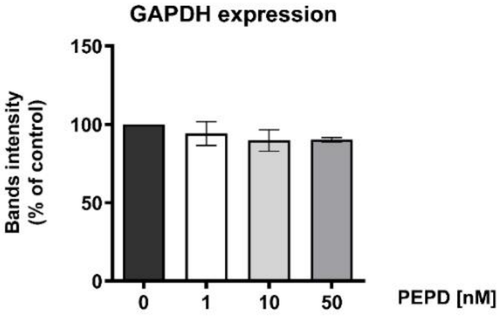

Figure S10. Representative blots from Western blotting analysis presented in Figure 9C.
